# The tremendous diversity of *Labiobaetis* Novikova & Kluge in Indonesia (Ephemeroptera, Baetidae)

**DOI:** 10.3897/zookeys.895.38576

**Published:** 2019-12-04

**Authors:** Thomas Kaltenbach, Jean-Luc Gattolliat

**Affiliations:** 1 Museum of Zoology, Palais de Rumine, Place Riponne 6, CH-1005 Lausanne, Switzerland Museum of Zoology Lausanne Switzerland; 2 University of Lausanne (UNIL), Department of Ecology and Evolution, CH-1015 Lausanne, Switzerland University of Lausanne Lausanne Switzerland

**Keywords:** COI, integrative taxonomy, mayflies, morphology, new species, Southeast Asia, species delimitation

## Abstract

Material collected between 2010 and 2014 on the Indonesian islands of Sumatra, Bali, Sumba, Sumbawa, Sulawesi, and Seram unveiled the enormous diversity of *Labiobaetis* Novikova & Kluge in this country. Five species were reported from Indonesia previously (*L. fulmeki* (Ulmer), *L. obscurum* (Ulmer), *L. necopinatum* (Müller-Liebenau), *L. ulmeri* (Müller-Liebenau), and *L. boettgeri* (Ulmer)); all were described from adults only and no species were previously known at larval stage. We identified 18 new species by integrative taxonomy using genetic distance (COI, Kimura-2-parameter) and morphology, and they are described and illustrated based on their larvae. Another species, *L. multus* (Müller-Liebenau) from Malaysia, was also found in Indonesia, increasing the total number of species in Indonesia to 24. Seven morpho-groups of species are proposed based on morphological characters and a key to the larvae of all species from Indonesia and adjacent countries is provided. The total number of *Labiobaetis* species worldwide is augmented to 123. The examination of the new species allowed us to slightly modify the generic attributes of the larvae. The interspecific K2P distances are usually between 11% and 24%, the intraspecific distances are usually between 0% and 3%. The remarkable richness of the genus in Indonesia is discussed.

## Introduction

The family Baetidae has the highest species diversity among mayflies, comprising 1,070 species in 110 genera (Sartori and Brittain 2015, Jacobus et al. 2019), which is approximately one quarter of all mayfly species worldwide (Gattolliat and Nieto 2009, Jacobus et al. 2019). They have a cosmopolitan distribution with the exception of Antarctica and New Zealand. Investigations of the molecular phylogeny of the Order Ephemeroptera revealed the relatively primitive status of the family (Ogden and Whiting 2005, Ogden et al. 2009).

The genus *Labiobaetis* Novikova & Kluge, 1987 is one of the richest genera of Baetidae with 105 previously described species (Barber-James et al. 2013, Webb 2013, Shi and Tong 2014, Kubendran et al. 2014, 2015, Gattolliat et al. 2018, Kaltenbach and Gattolliat 2018). The distribution of *Labiobaetis* is nearly worldwide, with the exception of the Neotropical realm and New Caledonia; it is widely diversified in the Afrotropical (28 species) and Oriental realms (23 species) as well as in New Guinea (32 species) (Lugo-Ortiz et al. 1999, Gattolliat and Nieto 2009, Kaltenbach and Gattolliat 2018). The status and validity of the genus has long been a subject of controversy, but today *Labiobaetis* is widely accepted as a valid genus (Lugo-Ortiz and McCafferty 1997, Lugo-Ortiz et al. 1999, Gattolliat 2001, Fujitani et al. 2003, Fujitani 2008, McCafferty et al. 2010, Gattolliat and Staniczek 2011, Kluge and Novikova 2011, 2014, 2016, Kluge 2012, Webb 2013, Kubendran et al. 2014, 2015, Shi and Tong 2014). The history and concept of the genus *Labiobaetis* were recently summarized in detail (Shi and Tong 2014, Kaltenbach and Gattolliat 2018). All Oriental species previously transferred to *Pseudocloeon* (Lugo-Ortiz et al. 1999) were formerly reassigned to *Labiobaetis* by Shi and Tong (2014). Molecular reconstructions indicated that the concept of *Labiobaetis* is probably at least diphyletic (Monaghan et al. 2005, Gattolliat et al. 2008).

Indonesia is an immense archipelago of more than 18,000 islands extending more than 5,000 km, from 95°E to 141°E and from 6°N to 11°S. It is one of the most biologically rich countries in the world. The high levels of species richness and endemism are mainly attributable to a complex geological history that brought together two different biological realms (Oriental realm and Australasian realm), separated by a transitional region (Wallacea) (Kingston 2010, Hall 2010). The main islands are Sumatra, Java, Borneo (partly, Kalimantan Province), Sulawesi, and New Guinea (partly, provinces West Papua and Papua). Furthermore, there are big island groups like the Lesser Sunda Islands, the Moluccas, and the Banda Islands.

The diversity of *Labiobaetis* in Indonesia was poorly known. Five species were reported from Indonesia (*L. fulmeki* (Ulmer), *L. obscurum* (Ulmer), *L. necopinatum* (Müller-Liebenau), *L. ulmeri* (Müller-Liebenau), and *L. boettgeri* (Ulmer)). All were described from adults only and no species were known at the larval stage (Ulmer 1913, 1924, 1939, Müller-Liebenau 1981). The generic attribution of these species is still controversial as *Labiobaetis* remains difficult to delimit at the imaginal stage. Here, we describe 18 new species of *Labiobaetis* based on larvae collected at different locations in Indonesia (Sumatra, Bali, Sumba, Sumbawa, Sulawesi, Seram; Fig. 53a) between 2010 and 2014; adults were not collected. We also report another species already known from Malaysia (*L. multus* (Müller-Liebenau)). Thereby, we consider *Labiobaetis* sensu lato, even if we presume that the genus is probably polyphyletic. We are currently still missing morphological characters and especially genetic evidence to split the genus into monophyletic lineages. Genetic studies on species from all realms involving nuclear genes are necessary to unveil the generic delimitation of *Labiobaetis* at a later point in time.

Material from the island of Borneo, including the Indonesian province Kalimantan as well as Brunei and the Malaysian province Sabah, will be treated in a separate paper (Kaltenbach and Gattolliat in press); material from the Papua Province of Indonesia was already treated in a recent paper (Kaltenbach and Gattolliat 2018).

## Materials and methods

The specimens were mainly collected by Michael Balke (Zoologische Staatssammlung München, ZSM, Germany). Further material was collected by Jean-Marc Elouard (France) and Morgan Gueuning (University of Neuchâtel, Switzerland; Gueuning et al. 2017).

The specimens were preserved in 70%–96% ethanol. The dissection of larvae was done in Cellosolve (2-Ethoxyethanol) with subsequent mounting on slides with Euparal liquid, using an Olympus SZX7 stereomicroscope.

The DNA of part of the specimens was extracted using non-destructive methods allowing subsequent morphological analysis (see Vuataz et al. 2011 for details). We amplified a 658 bp fragment of the mitochondrial gene cytochrome oxidase subunit 1 (COI) using the primers LCO 1490 (GGTCAACAAATCATAAAGATATTGG) and HCO 2198 (TAAACTTCAGGGTGACCAAAAAATCA) (Folmer et al. 1994). The polymerase chain reaction was conducted with an initial denaturation temperature of 98 °C for 30 sec followed by a total of 37 cycles with denaturation temperature of 98 °C for 10 sec, an annealing temperature of 50 °C for 30 sec and an extension at 72 °C for 30 sec, final extension at 72 °C for 2 min. Sequencing was done with Sanger’s method (Sanger et al. 1977). The genetic variability between specimens was estimated using Kimura-2-parameter distances (K2P, Kimura 1980), calculated with the program MEGA 7 (Kumar et al. 2016, http://www.megasoftware.net). The GenBank accession numbers are given in Table 1, nomenclature of gene sequences is according to Chakrabarty et al. (2013).

**Table 1.** Sequenced specimens.

**Species**	**Locality**	**Specimen catalog** #	**GenBank** #	**GenSeq**
			(COI)	Nomenclature
*L. batakorum* sp. nov.	Sumatra	GBIFCH 00529194	MN167343	genseq-1 COI
		GBIFCH 00592195	MN167352	genseq-2 COI
		GBIFCH 00592200	MN167351	genseq-4 COI
		GBIFCH 00592197	MN167350	genseq-4 COI
*L. sulawesiensis* sp. nov.	Sulawesi	GBIFCH 00235812	MN167327	genseq-1 COI
*L. sumbensis* sp. nov.	Sumba	GBIFCH 00592191	MN167342	genseq-2 COI
		GBIFCH 00235825	MN167325	genseq-2 COI
		GBIFCH 00657749	MN167322	genseq-1 COI
*L. weifangae* sp. nov.	Sumbawa	GBIFCH 00592228	MN167344	genseq-1 COI
		GBIFCH 00657740	MN167328	genseq-2 COI
		GBIFCH 00657743	MN167330	genseq-2 COI
	Sumba	GBIFCH 00592229	MN167346	genseq-2 COI
*L. itineris* sp. nov.	Sumbawa	GBIFCH 00592226	MN167340	genseq-2 COI
	Bali	GBIFCH 00592225	MN167341	genseq-1 COI
*L. lubu* sp. nov.	Sumatra	GBIFCH 00592217	MN167339	genseq-2 COI
*L. pakpak* sp. nov.	Sumatra	GBIFCH 00235851	MN167321	genseq-2 COI
		GBIFCH 00235781	MN167329	genseq-2 COI
*L. paradiffundus* sp. nov.	Sumatra	GBIFCH 00592213	MN167355	genseq-1 COI
		GBIFCH 00592214	MN167356	genseq-2 COI
*L. gueuningi* sp. nov.	Sumatra	GBIFCH 00465243	MN167353	genseq-2 COI
		GBIFCH 00465244	MN167354	genseq-2 COI
		GBIFCH 00422156	MN167331	genseq-2 COI
		GBIFCH 00422153	MN167332	genseq-1 COI
		GBIFCH 00422190	MN202466	genseq-2 COI
		GBIFCH 00422197	MN202467	genseq-2 COI
		GBIFCH 00422200	MN202468	genseq-2 COI
		GBIFCH 00422147	MN202469	genseq-2 COI
		GBIFCH 00422149	MN202470	genseq-2 COI
*L. minang* sp. nov.	Sumatra	GBIFCH 00422537	MN167335	genseq-2 COI
		GBIFCH 00422521	MN167334	genseq-2 COI
		GBIFCH 00422456	MN167336	genseq-2 COI
		GBIFCH 00422498	MN167333	genseq-1 COI
*L. paranumeratus* sp. nov.	Sumatra	GBIFCH 00592211	MN167349	genseq-2 COI
		GBIFCH 00592210	MN167348	genseq-1 COI
*L. pilosus* sp. nov.	Sulawesi	GBIFCH 00592203	MN167347	genseq-2 COI
		GBIFCH 00592201	MN167345	genseq-1 COI
		GBIFCH 00657738	MN167324	genseq-2 COI
		GBIFCH 00657734	MN167326	genseq-2 COI
*L. multus* (Müller-Liebenau)	Sumatra	GBIFCH 00235847	MN167323	genseq-4 COI
*L. jonasi* sp. nov.	Sumba	GBIFCH 00654966	MN167337	genseq-2 COI
		GBIFCH 00654967	MN167338	genseq-2 COI

Drawings were made using an Olympus BX43 microscope. Photographs of larvae were taken using a Canon EOS 6D camera and the Visionary Digital Passport imaging system (http://www.duninc.com) and processed with the programs Adobe Photoshop Lightroom (http://www.adobe.com) and Helicon Focus version 5.3 (http://www.heliconsoft.com). Photographs were subsequently enhanced with Adobe Photoshop Elements 13.

The distribution map was generated with the program QGIS (http://qgis.org), the program GEOLocate (http://www.museum.tulane.edu/geolocate/web/WebGeoref.aspx) and Google Earth (http://www.google.com/earth/download/ge/) were used to attribute approximate GPS coordinates to sample locations of Müller-Liebenau (1982, 1984a, b) and Soldan (1991).

The taxonomic descriptions and the key presented herein were generated with a DELTA (Dallwitz 1980, Dallwitz et al. 1999, Coleman et al. 2010) database containing the morphological states of characters of the *Labiobaetis* species of Indonesia and Southeast Asia.

The new species described in this study were all compared to paratypes (on slides) of the species already known from Southeast Asia (excluding Southern China; deposited in Zoologische Staatssammlung München, ZSM).

For the terminology we are referring to Hubbard (1995) and to Morihara and McCafferty (1979). The postero-lateral extension of the paraproct is termed cercotractor following Kluge (2004).

## Results

### New species descriptions

Abbreviations:

**MZL** Museum of Zoology Lausanne (Switzerland)

**ZSM** Zoologische Staatssammlung München (Germany)

**MZB** Museum Zoologicum Bogoriense (Indonesia)

### List of *Labiobaetis* species from Indonesia and adjacent countries (Malaysia, Vietnam, Philippines)

*batakorum* group

1. *L. batakorum* sp. nov.

2. *L. sulawesiensis* sp. nov.

3. *L. sumbensis* sp. nov.

*difficilis* group

4. *L. difficilis* (Müller-Liebenau, 1984) (Malaysia)

5. *L. roulade* sp. nov.

6. *L. weifangae* sp. nov.

*sumigarensis* group (Müller-Liebenau and Hubbard 1985)

7. *L. diffundus* (Müller-Liebenau, 1984) (Malaysia)

8. *L. molawinensis* (Müller-Liebenau, 1982) (Philippines)

9. *L. sumigarensis* (Müller-Liebenau, 1982) (Philippines)

10. *L. itineris* sp. nov.

11. *L. lubu* sp. nov.

12. *L. pakpak* sp. nov.

13. *L. paradiffundus* sp. nov.

14. *L. rimba* sp. nov.

*gueuningi* group

15. *L. gueuningi* sp. nov.

16. *L. minang* sp. nov.

*numeratus* group

17. *L. numeratus* (Müller-Liebenau, 1984) (Malaysia)

18. *L. paranumeratus* sp. nov.

19. *L. pilosus* sp. nov.

*operosus* group

20. *L. operosus* (Müller-Liebenau, 1984) (Malaysia)

21. *L. paraoperosus* sp. nov.

*seramensis* group

22. *L. seramensis* sp. nov.

23. *L. wahai* sp. nov.

not assigned to a group

24. *L. borneoensis* (Müller-Liebenau, 1984) (Malaysia)

25. *L. moriharai* (Müller-Liebenau, 1984) (Malaysia, Vietnam)

26. *L. multus* (Müller-Liebenau, 1984) (Malaysia, Indonesia)

27. *L. jonasi* sp. nov.

**Figure 1.**
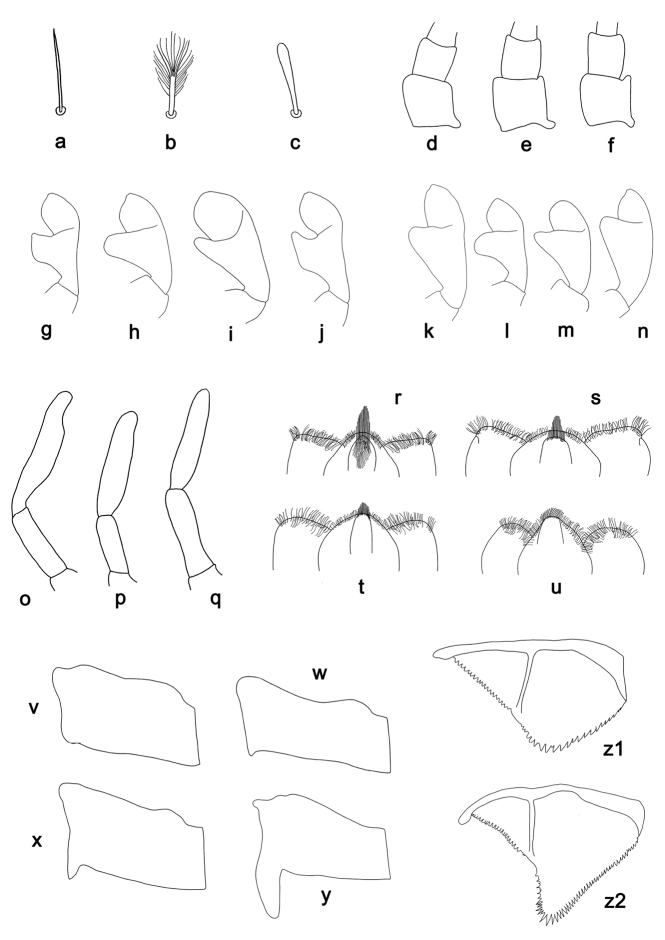
*Labiobaetis*, character states of selected characters: **a–c** setae of the submarginal arc on the dorsal surface of the labrum, **a** simple **b** feathered **c** clavate; **d–f** distolateral process at scape of antenna, **d** absent **e** poorly developed **f** well developed; **g–j** labial palp, distomedial protuberance of segment II, **g** lobed **h** thumb-like **i** slender thumb-like **j** hook-like; **k–n** labial palp, shape of segment III, **k** oblong, distally with small projection **l** slightly pentagonal, distally slightly pointed **m** approx. semicircular **n** conical, distally slightly truncate; **o–q** distolateral excavation at maxillary palp segment II, **o** well developed **p** poorly developed **q** absent; **r–u** hypopharynx, medial tuft of stout setae, **r** well developed, long **s** well developed, average length **t** well developed, short **u** poorly developed; **v–y** hindwing pads, **v** absent **w** minute **x** small **y** well developed; **z1, z2** paraproct, **z1** distally not expanded **z2** distally expanded.

### *Labiobaetis batakorum* group of species

With the following combination of characters: A) dorsal surface of labrum with submarginal arc of simple setae; B) labial palp segment II with large, lobed distomedial protuberance; C) left mandible without setae at apex of mola; D) lingua of hypopharynx with well developed, short medial tuft of setae; E) dorsal margin of femur with 9–11 setae; F) seven pairs of gills; G) paraproct distally not expanded; H) hindwing pads absent; I) distolateral process at scape well-developed.

#### 1. Labiobaetis batakorum sp. nov.

Taxon classificationAnimaliaEphemeropteraBaetidae

374D6453-1F18-5A93-8034-6BF8496FD4AF

http://zoobank.org/195D71DD-0C6E-4629-9D06-1D145E59EBEB

**Figure 2.**
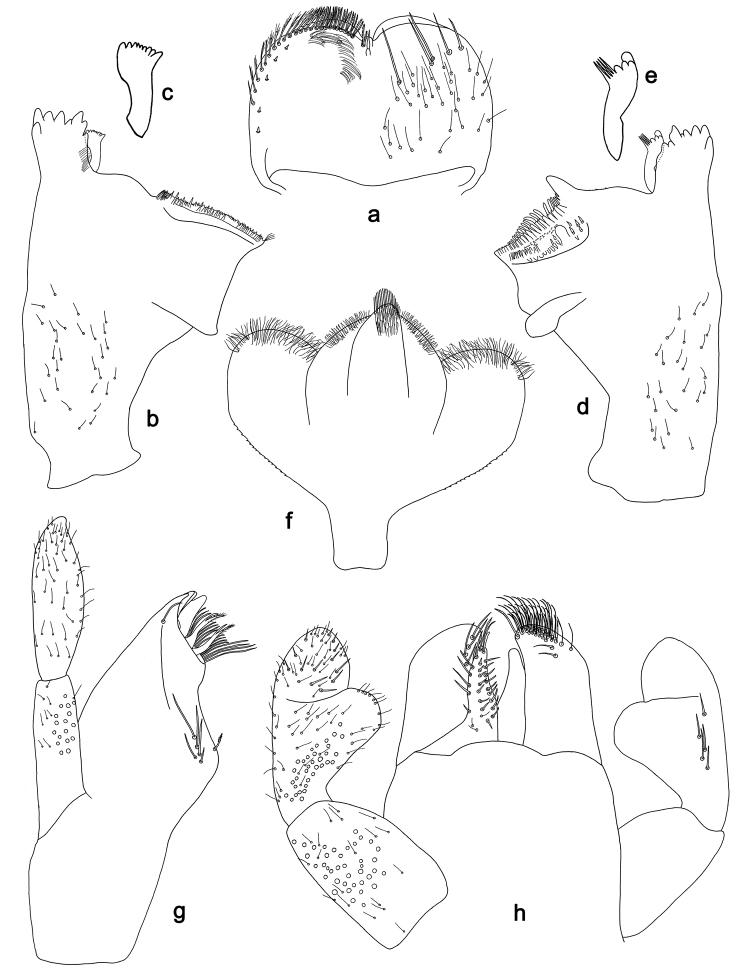
*Labiobaetis batakorum* sp. nov., larva morphology: **a** Labrum **b** Right mandible **c** Right prostheca **d** Left mandible **e** Left prostheca **f**Hypopharynx**g** Maxilla **h** Labium.

**Figure 3.**
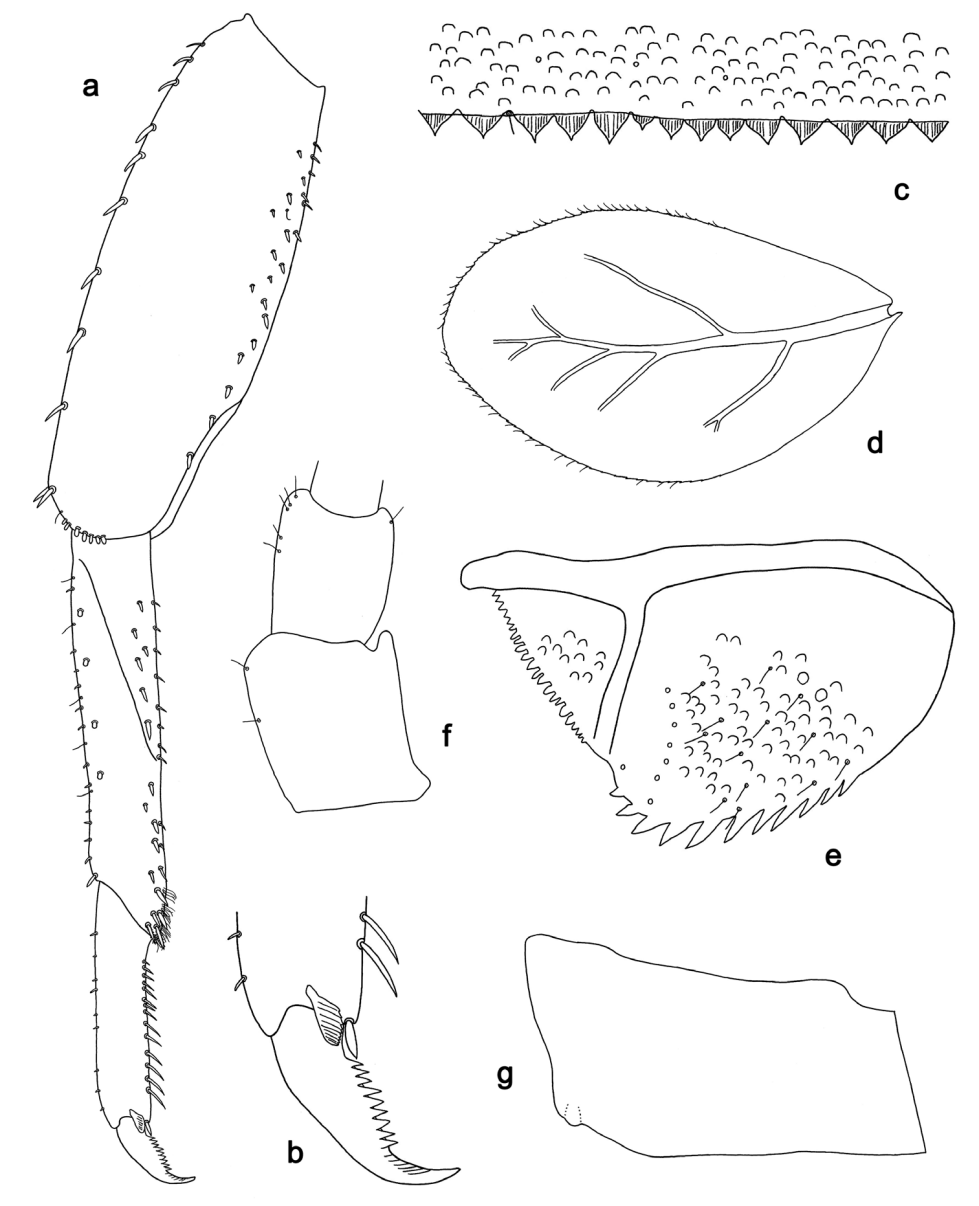
*Labiobaetis batakorum* sp. nov., larva morphology: **a** Foreleg **b** Fore claw **c** Tergum IV **d** Gill IV **e** Paraproct **f** Scape **g** Metanotum.

Figures 2 , 3 , 47a , 52a , 54a

##### Diagnosis.

**Larva.** Following combination of characters: A) dorsal surface of labrum with submarginal arc of 1 + 4 long, simple setae; B) labial palp segment II with a broad, thumb-like distomedial protuberance, segment III conical; C) fore femur rather broad, length ca. 3× maximum width, dorsal margin with a row of ca. nine curved, spine-like setae; D) fore claw with 9–12 denticles; E) paraproct distally not expanded, with 11–18 stout marginal spines.

##### Description.

**Larva** (Figs 2, 3, 47a, 52a). Body length 4.5–4.7 mm; antenna approximately 2.5× as long as head length.

*Colouration*. Head, thorax, and abdomen dorsally brown, head and thorax with bright, median, dorsal suture, thorax and abdomen with pattern as in Fig. 47a, forewing pads with brown striation. Head, thorax, and abdomen ventrally light brown, legs transparent, femur with a distomedial brown spot and apex brown, tibia and tarsus distally brown (Fig. 52a). Caudal filaments light brown with a dark brown band at ca. 1/2 of cerci.

*Antenna* with scape and pedicel subcylindrical, with well-developed distolateral process at scape (Fig. 3f); flagellum with broad, lanceolate spines and fine, simple setae on apex of each segment.

*Labrum* (Fig. 2a). Rectangular, length 0.7× maximum width. Distal margin with medial emargination and a small process. Dorsally with long, fine, simple setae scattered over surface; submarginal arc of setae composed of 1 + 4 long, simple setae, the first two setae after the central seta are closely together. Ventrally with marginal row of setae composed of lateral and anterolateral long, feathered setae and medial long, bifid, pectinate setae; ventral surface with five short, spine-like setae near lateral and anterolateral margin.

*Right mandible* (Fig. 2b, c). Incisors fused. Outer and inner sets of denticles with 4 + 3 denticles and one minute intermediate denticle. Inner margin of innermost denticle with a row of thin setae. Prostheca robust, apically denticulate. Margin between prostheca and mola slightly convex. Tuft of setae at apex of mola present.

*Left mandible* (Fig. 2d, e). Incisors fused. Outer and inner sets of denticles with 4 + 3 denticles and one minute intermediate denticle. Prostheca robust, apically with small denticles and comb-shaped structure. Margin between prostheca and mola slightly convex, with minute denticles toward subtriangular process. Subtriangular process long and slender, above level of area between prostheca and mola. Denticles of mola apically constricted. Tuft of setae at apex of mola absent.

Both mandibles with lateral margins almost straight. Basal half with fine, simple setae scattered over dorsal surface.

*Hypopharynx* (Fig. 2f). Lingua longer than superlingua. Lingua approx. as broad as long; medial tuft of stout setae well developed, short; distal half not expanded. Superlingua rounded; lateral margin rounded; fine, long, simple setae along distal margin.

*Maxilla* (Fig. 2g). Galea-lacinia with one simple, robust apical seta under crown. Inner dorsal row of setae with three denti-setae, distal denti-seta tooth-like, middle and proximal denti-setae slender, bifid and pectinate. Medially with one bipectinate, spine-like seta and 4–5 long, simple setae. Maxillary palp 1.3× as long as length of galea-lacinia; two segmented; palp segment II 1.2× length of segment I; setae on maxillary palp fine, simple, scattered over surface of segments I and II; apex of last segment rounded, with slight excavation at inner distolateral margin.

*Labium* (Fig. 2h). Glossa basally broad, narrowing toward apex; shorter than paraglossa; inner margin with 8–9 spine-like setae, distalmost seta much longer than other setae; apex with two long, robust, pectinate setae and one medium, robust seta; outer margin with 5–6 spine-like setae increasing in length distally; ventral surface with short, fine, simple and short, spine-like setae. Paraglossa sub-rectangular, curved inward; apex rounded; with three rows of long, robust, distally pectinate setae in apical area and two medium, simple setae in anteromedial area; dorsally with a row of four long, spine-like setae near inner margin. Labial palp with segment I 0.9× length of segments II and III combined. Segment I ventrally with short, fine, simple setae. Segment II with broad thumb-like distomedial protuberance; distomedial protuberance 0.5× width of base of segment III; inner and outer margin with short, fine, simple setae; dorsally with a row of 4–5 long, spine-like, simple setae near outer margin. Segment III conical; apex rounded; length 1.0× width; ventrally covered with short and medium spine-like, simple setae and short, fine, simple setae.

*Hind wing pads* absent (Fig. 3g).

*Foreleg* (Fig. 3a, b). Ratio of foreleg segments 1.4:1.0:0.6:0.2. *Femur*. Length ca. 3× maximum width. Dorsal margin with a row of ca. nine curved, spine-like setae; length of setae 0.13× maximum width of femur. Apex rounded; with one pair of spine-like setae and some short, stout setae. Many stout, lanceolate setae scattered along ventral margin; femoral patch absent. *Tibia.* Dorsal margin with a row of short, spine-like setae and fine, simple setae, near margin some stout, apically rounded setae; on apex one longer, spine-like, apically rounded seta. Ventral margin with a row of curved, spine-like setae, on apex some spine-like, partly bipectinate setae and a tuft of long, fine, simple setae. Anterior surface scattered with stout, lanceolate setae. Patellotibial suture present on basal 1/2. *Tarsus.* Dorsal margin with a row of short, stout setae. Ventral margin with a row of curved, spine-like setae. Tarsal claw with one row of 9–12 denticles; distally pointed; with 5–7 stripes; subapical setae absent.

*Tergum* (Fig. 3c). Surface with irregular rows of U-shaped scale bases and scattered micropores. Posterior margin of tergum IV with triangular spines, wider than long.

*Gills* (Fig. 3d). Present on segments I–VII. Margin with small denticles intercalating fine simple setae. Tracheae extending from main trunk to inner and outer margins. Gill I ca. 2/3 length of segment II. Gill IV as long as length of segments V and 2/3 VI combined. Gill VII as long as length of segments VIII and 1/3 IX combined.

*Paraproct* (Fig. 3e). Distally not expanded, with 11–18 stout marginal spines. Surface scattered with U-shaped scale bases, fine, simple setae and micropores. Cercotractor with numerous small marginal spines.

##### Etymology.

Dedicated to the indigenous Batak people from Sumatra.

##### Distribution.

Indonesia: Sumatra.

##### Biological aspects.

The specimens were collected at altitudes from sea level to 280 m.

##### Type-material.

**Holotype.** Larva (on slide, GBIFCH 00592194), Indonesia, Sumatra Barat, Sawahlunto, stream, 275 m, 10.XI.2011, 00°41.33'S, 100°46.72'E, M. Balke leg. (UN5). Temporary deposited in MZL before definitely housed in MZB. **Paratypes.** 11 larvae (2 on slides, GBIFCH 00592195, GBIFCH 00592196, 7 in alcohol, GBIFCH 00515349, deposited in MZL; 2 in alcohol, GBIFCH 00515347, deposited in ZSM), same data as holotype.

##### Other material.

2 larvae (on slides, GBIFCH 00592200, GBIFCH 00592199, deposited in MZL), Indonesia, Sumatra Barat, Barung-Barung, 01°06.03'S, 100°29.12'E, 75 m, 24.V.2010, J.-M. Elouard leg.; 4 larvae (2 on slides, GBIFCH 00592197, GBIFCH 00592198, 2 in alcohol, GBIFCH 00515365, deposited in MZL), Indonesia, Sumatra Barat, Tarusan, upstream Tarusan, 01°13.62'S, 100°29.83'E, 10 m, 24.V.2010, J.-M. Elouard leg.

#### 2. Labiobaetis sulawesiensis sp. nov.

Taxon classificationAnimaliaEphemeropteraBaetidae

3C78520A-79E6-5F2B-A95C-4B628C0E9388

http://zoobank.org/37E62B2D-87F6-42A7-A391-30F5A5674AC9

**Figure 4.**
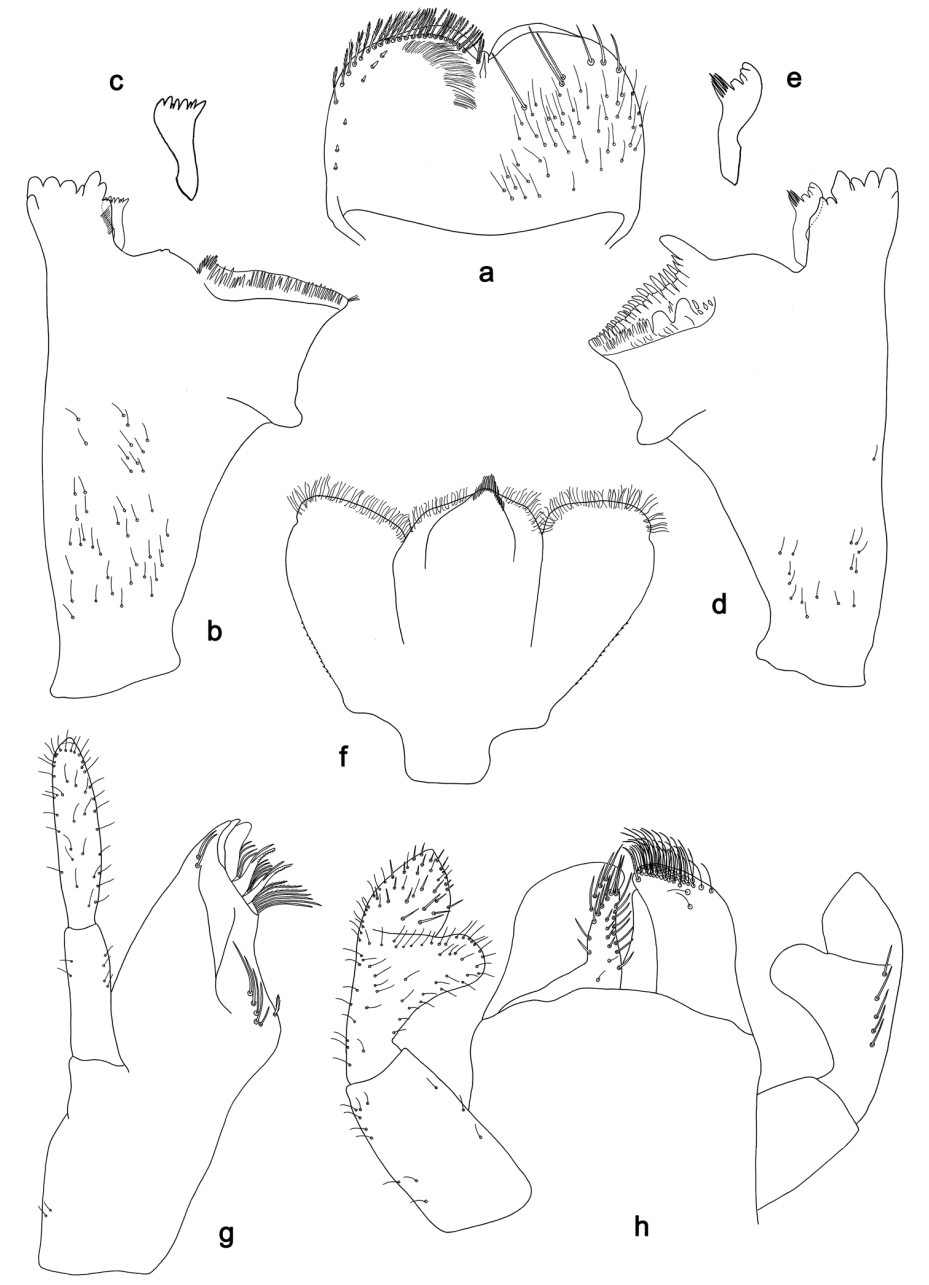
*Labiobaetis sulawesiensis* sp. nov., larva morphology: **a** Labrum **b** Right mandible **c** Right prostheca **d** Left mandible **e** Left prostheca **f**Hypopharynx**g** Maxilla **h** Labium.

**Figure 5.**
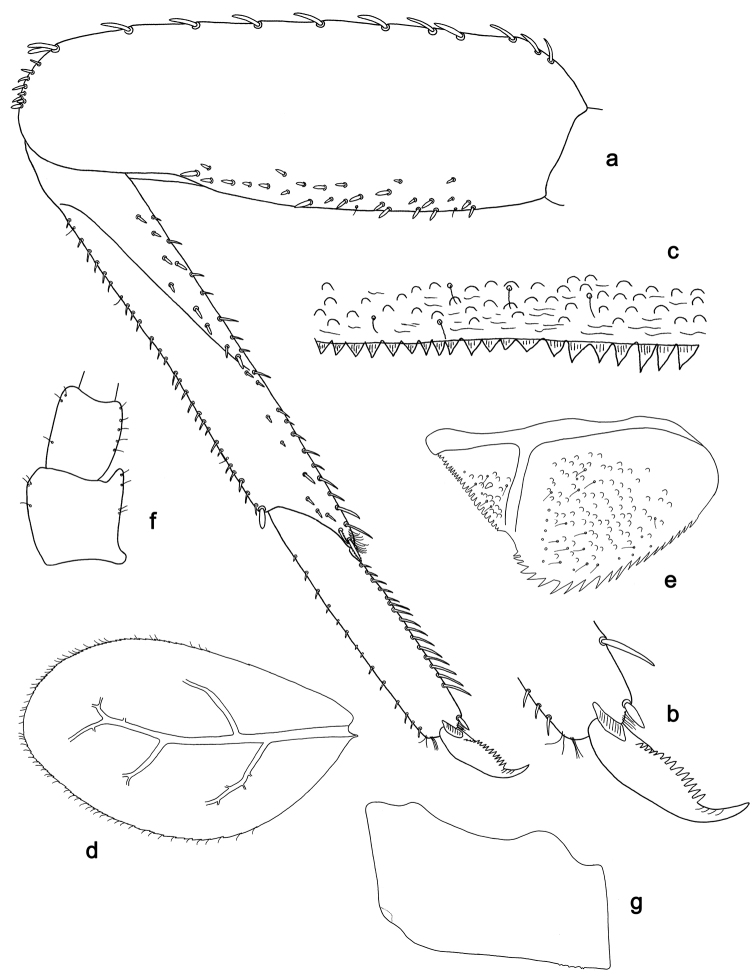
*Labiobaetis sulawesiensis* sp. nov., larva morphology: **a** Foreleg **b** Fore claw **c** Tergum IV **d** Gill IV **e** Paraproct **f** Scape **g** Metanotum.

Figures 4 , 5 , 47b , 54a

##### Diagnosis.

**Larva.** Following combination of characters: A) dorsal surface of labrum with submarginal arc of 1 + 5 long, simple setae; B) labial palp segment II with an elongated thumb-like distomedial protuberance, segment III conical; C) fore femur rather broad, length ca. 3× maximum width, dorsal margin with a row of ca. ten curved, spine-like setae; D) paraproct distally not expanded, with ca. 21 stout marginal spines.

##### Description.

**Larva** (Figs 4, 5, 47b). Body length 4.7 mm; antenna approximately twice as long as head length.

*Colouration*. Head, thorax, and abdomen dorsally brown, head and thorax with bright median, dorsal suture, thorax with pattern as in Fig. 47b, abdominal segments I, V, VI, IX, and X bright, segment IV partly bright. Head, thorax, and abdomen ventrally light brown, abdominal segments VII and VIII dark brown, legs light brown, femur with a distomedial brown spot, caudal filaments light brown, with a dark brown band at 2/3 of cerci.

*Antenna* with scape and pedicel subcylindrical, with well-developed distolateral process at scape; flagellum with lanceolate spines and fine, simple setae on apex of each segment.

*Labrum* (Fig. 4a). Rectangular, length 0.7× maximum width. Distal margin with medial emargination and a small process. Dorsally with medium, fine, simple setae scattered over surface; submarginal arc of setae composed of 1 + 5 long, simple setae. Ventrally with marginal row of setae composed of lateral and anterolateral long, feathered setae and medial long, bifid, pectinate setae; ventral surface with six short, spine-like setae near lateral and anterolateral margin.

*Right mandible* (Fig. 4b, c). Incisors fused. Outer and inner sets of denticles with 4 + 3 denticles and one minute intermediate denticle. Inner margin of innermost denticle with a row of thin setae. Prostheca robust, apically denticulate. Margin between prostheca and mola slightly convex, with minute denticles. Tuft of setae at apex of mola present.

*Left mandible* (Fig. 4d, e). Incisors fused. Outer and inner sets of denticles with 4 + 3 denticles and one minute intermediate denticle. Prostheca robust, apically with small denticles and comb-shaped structure. Margin between prostheca and mola slightly convex. Subtriangular process long and slender, above level of area between prostheca and mola. Denticles of mola apically constricted. Tuft of setae at apex of mola absent.

Both mandibles with lateral margins almost straight. Basal half with fine, simple setae scattered over dorsal surface.

*Hypopharynx* (Fig. 4f). Lingua approx. as long as superlingua. Lingua longer than broad; medial tuft of stout setae well developed, short; distal half not expanded. Superlingua straight; lateral margin rounded; fine, long, simple setae along distal margin.

*Maxilla* (Fig. 4g). Galea-lacinia with two simple, robust apical setae under crown. Inner dorsal row of setae with three denti-setae, distal denti-seta tooth-like, middle and proximal denti-setae slender, bifid and pectinate. Medially with one bipectinate, spine-like seta and five medium to long simple setae. Maxillary palp 1.3× as long as length of galea-lacinia; two segmented; palp segment II 1.4× length of segment I; setae on maxillary palp fine, simple, scattered over surface of segments I and II; apex of last segment constricted, without excavation at inner distolateral margin.

*Labium* (Fig. 4h). Glossa basally broad, narrowing toward apex; shorter than paraglossa; inner margin with eight spine-like setae increasing in length distally; apex with two long and one medium, robust, pectinate setae; outer margin with six spine-like setae; ventral surface with short, fine, simple and short, spine-like setae. Paraglossa sub-rectangular, curved inward; apex rounded; with three rows of long, robust, distally pectinate setae in apical area and two medium, simple setae in anteromedial area; dorsally with a row of four long, spine-like setae near inner margin. Labial palp with segment I 0.8× length of segments II and III combined. Segment I with fine, simple setae along margins. Segment II with elongated, thumb-like distomedial protuberance; distomedial protuberance 0.7× width of base of segment III; inner and outer margin with short, fine, simple setae; dorsally with a row of five long, spine-like, simple setae near outer margin. Segment III conical; apex slightly pointed; length 1.1× width; ventrally covered with short and medium spine-like, simple setae and short, fine, simple setae.

*Hind wing pads* absent (Fig. 5g).

*Foreleg* (Fig. 5a, b). Ratio of foreleg segments 1.2:1.0:0.6:0.2. *Femur*. Length ca. 3× maximum width. Dorsal margin with a row of ca. ten curved, spine-like setae; length of setae 0.16× maximum width of femur. Apex rounded; with one pair of curved, spine-like setae and some short, stout setae. Many stout, lanceolate setae scattered along the ventral margin; femoral patch absent. *Tibia*. Dorsal margin with a row of curved, spine-like setae; on apex one longer, spine-like, apically rounded seta. Ventral margin with a row of curved, spine-like setae, on apex several spine-like, bipectinate setae and a tuft of fine, simple setae. Anterior surface scattered with stout, lanceolate setae. Patellotibial suture present on basal 2/3. *Tarsus*. Dorsal margin with a row of short, curved, spine-like setae, on apex fine, simple setae. Ventral margin with a row of curved, spine-like setae. Tarsal claw with one row of 12–13 denticles; distally pointed; with 4–5 stripes; subapical setae absent.

*Tergum* (Fig. 5c). Surface with irregular rows of U-shaped scale bases and scattered fine, simple setae. Posterior margin of tergum IV with triangular spines, medially longer than wide, laterally shorter and approx. as long as wide.

*Gills* (Fig. 5d). Present on segments I–VII. Margin with small denticles intercalating fine simple setae. Tracheae extending from main trunk to inner and outer margins. Gill I ca. 1/2 length of segment II. Gill IV as long as length of segments V and 2/3 VI combined. Gill VII little longer than length of segment VIII.

*Paraproct* (Fig. 5e). Distally not expanded, with ca. 21 stout marginal spines. Surface scattered with U-shaped scale bases, fine, simple setae and micropores. Cercotractor with numerous small marginal spines.

##### Etymology.

Refers to the island Sulawesi, where the specimens were collected.

##### Distribution.

Indonesia: Sulawesi.

##### Biological aspects.

The specimens were collected at an altitude of 660 m.

##### Type-material.

**Holotype.** Larva (on slide, GBIFCH 00235812), Indonesia, Sulawesi Tengah, Palu-Lake Lore, stream, 660 m, 01.IX.2011, 01°11.75'S, 120°10.20'E, M. Balke leg. (SUL012). Temporary deposited in MZL before finally housed in MZB. **Paratypes.** 2 larvae (1 on slide, GBIFCH 00592206, deposited in MZL; 1 in alcohol, GBIFCH 00515354, deposited in ZSM), same data as holotype.

#### 3. Labiobaetis sumbensis sp. nov.

Taxon classificationAnimaliaEphemeropteraBaetidae

ADF259DE-F95B-59FC-8C4B-5368DFA97997

http://zoobank.org/A6C2AB9F-9EBC-434A-AD50-84D31BF4F74B

**Figure 6.**
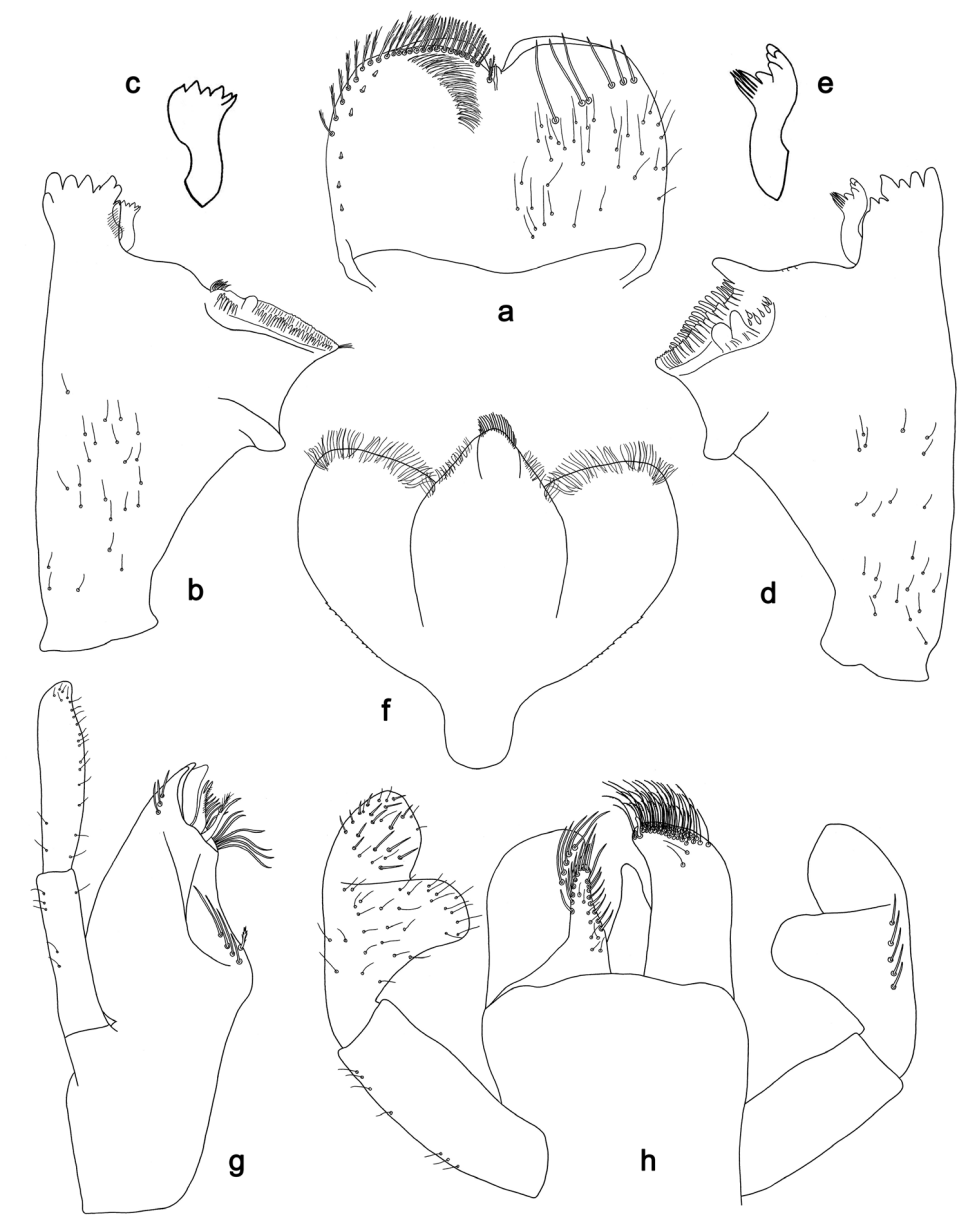
*Labiobaetis sumbensis* sp. nov., larva morphology: **a** Labrum **b** Right mandible **c** Right prostheca **d** Left mandible **e** Left prostheca **f**Hypopharynx**g** Maxilla **h** Labium.

**Figure 7.**
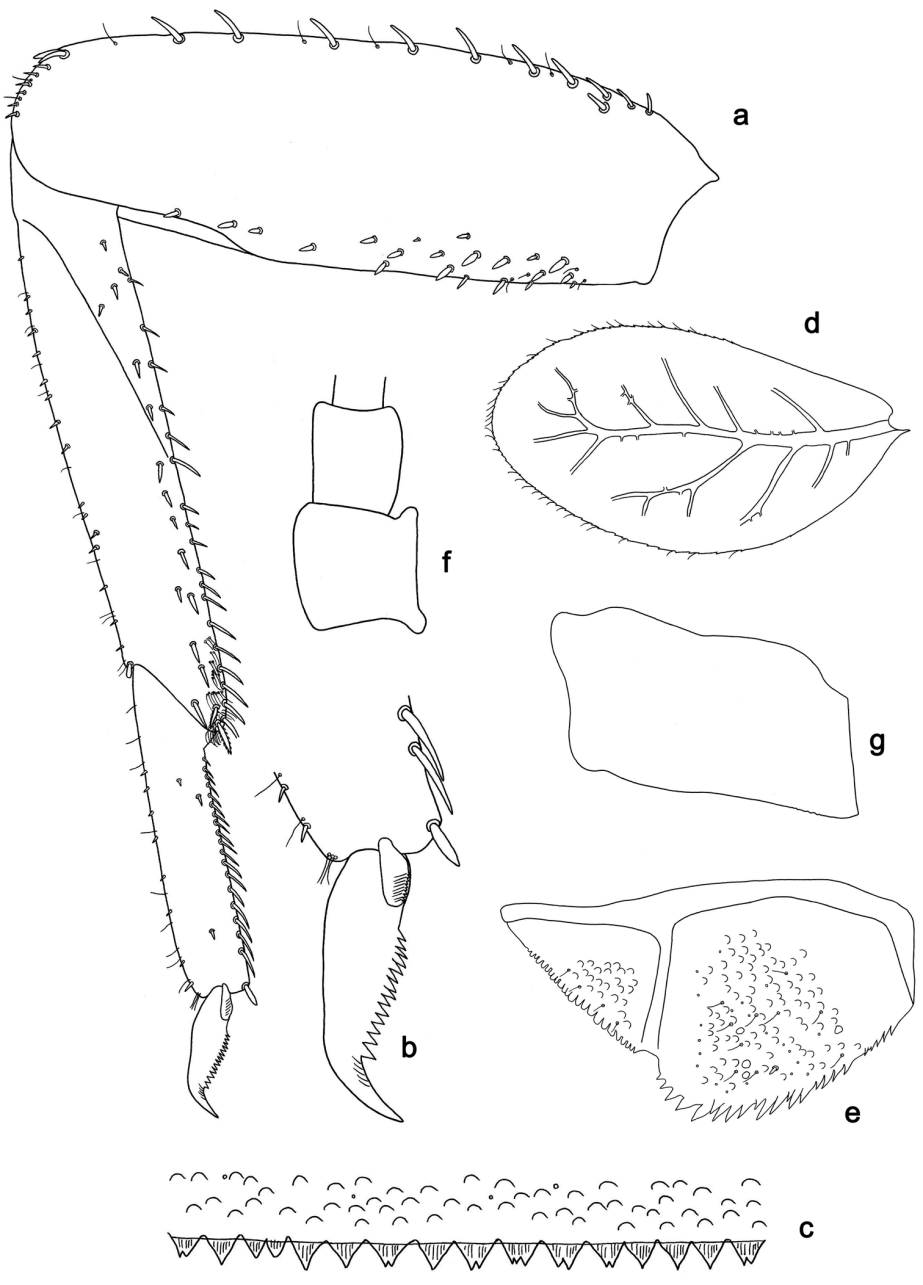
*Labiobaetis sumbensis* sp. nov., larva morphology: **a** Foreleg **b** Fore claw **c** Tergum IV **d** Gill IV **e** Paraproct **f** Scape **g** Metanotum.

Figures 6 , 7 , 47c , 52b , 54a

##### Diagnosis.

**Larva.** Following combination of characters: A) dorsal surface of labrum with submarginal arc of 1 + 5 long, simple setae; B) labial palp segment II with a large, lobed distomedial protuberance, segment III oblong; C) fore femur rather broad, length ca. 3× maximum width, dorsal margin with a row of ca. ten curved, spine-like setae; D) fore claw with 12–14 denticles; E) paraproct distally not expanded, with 19–24 stout marginal spines.

##### Description.

**Larva** (Figs 6, 7, 47c, 52b). Body length 4.4 mm; antenna approximately twice as long as head length.

*Colouration*. Head, thorax, and abdomen dorsally brown, head and thorax with bright median, dorsal suture, thorax and abdomen with pattern as in Fig. 47c, forewing pads with brown striation. Head, thorax, and abdomen ventrally brown, femur with dorsal and ventral brown streaks, proximally connected, distally brown and with a distomedial brown spot (Fig. 52b), tibia and tarsus light brown. Caudal filaments light brown, with a dark brown band at ca. 2/3 of cerci.

*Antenna* with scape and pedicel subcylindrical, with well-developed distolateral process at scape; flagellum with lanceolate spines and fine, simple setae on apex of each segment.

*Labrum* (Fig. 6a). Rectangular, length 0.7× maximum width. Distal margin with medial emargination and a small process. Dorsally with long, fine, simple setae scattered over surface; submarginal arc of setae composed of 1 + 5 long, simple setae, the first two setae after the central seta are closely together. Ventrally with marginal row of setae composed of lateral and anterolateral long, feathered setae and medial long, bifid, pectinate setae; ventral surface with six short, spine-like setae near lateral and anterolateral margin.

*Right mandible* (Fig. 6b, c). Incisors fused. Outer and inner sets of denticles with 4 + 3 denticles and one minute intermediate denticle. Inner margin of innermost denticle with a row of thin setae. Prostheca robust, apically denticulate. Margin between prostheca and mola slightly convex. Tuft of setae at apex of mola present.

*Left mandible* (Fig. 6d, e). Incisors fused. Outer and inner sets of denticles with 3 + 3 denticles. Prostheca robust, apically with small denticles and comb-shaped structure. Margin between prostheca and mola slightly convex, with minute denticles toward subtriangular process. Subtriangular process long and slender, above level of area between prostheca and mola. Denticles of mola apically constricted. Tuft of setae at apex of mola absent.

Both mandibles with lateral margins almost straight. Basal half with fine, simple setae scattered over dorsal surface.

*Hypopharynx* (Fig. 6f). Lingua approx. as long as superlingua. Lingua longer than broad; medial tuft of stout setae well developed, short; distal half not expanded. Superlingua rounded; lateral margin rounded; fine, long, simple setae along distal margin.

*Maxilla* (Fig. 6g). Galea-lacinia with three simple, robust apical setae under crown. Inner dorsal row of setae with three denti-setae, distal denti-seta tooth-like, middle and proximal denti-setae slender, bifid and pectinate. Medially with one bipectinate, spine-like seta and four long, simple setae. Maxillary palp 1.3× as long as length of galea-lacinia; two segmented; palp segment II 1.2× length of segment I; setae on maxillary palp fine, simple, scattered over surface of segments I and II; apex of last segment rounded, with slight excavation at inner distolateral margin.

*Labium* (Fig. 6h). Glossa basally broad, narrowing toward apex; shorter than paraglossa; inner margin with eight spine-like setae, distalmost seta much longer than other setae; apex with two long and one medium, robust, pectinate setae; outer margin with six spine-like setae; ventral surface with short, fine, simple setae. Paraglossa sub-rectangular, curved inward; apex rounded; with three rows of long, robust, distally pectinate setae in apical area and two medium, simple setae in anteromedial area; dorsally with a row of five long, spine-like setae near inner margin. Labial palp with segment I approx. as long as length of segments II and III combined. Segment I ventrally with short, fine, simple setae. Segment II with large, lobed distomedial protuberance; distomedial protuberance 0.7× width of base of segment III; inner and outer margin with short, fine, simple setae; dorsally with a row of five long, spine-like, simple setae near outer margin. Segment III oblong; apex rounded; length 1.1× width; ventrally covered with short and medium spine-like, simple setae and short, fine, simple setae.

*Hind wing pads* absent.

*Foreleg* (Fig. 7a, b). Ratio of foreleg segments 1.2:1.0:0.6:0.2. *Femur*. Length ca. 3× maximum width. Dorsal margin with a row of ca. ten curved, spine-like setae; length of setae 0.16× maximum width of femur. Apex rounded; with one pair of curved, spine-like setae and some short, stout setae. Many stout, lanceolate setae scattered along the ventral margin; femoral patch reduced to a few setae. *Tibia*. Dorsal margin with a row of short, stout setae and fine, simple setae; on apex one longer, spine-like, apically rounded seta. Ventral margin with a row of curved, spine-like setae, on apex several spine-like, partly bipectinate setae and a tuft of fine, simple setae. Anterior surface scattered with stout, lanceolate setae. Patellotibial suture present on basal 1/2. *Tarsus.* Dorsal margin with a row of short, stout setae and fine, simple setae. Ventral margin with a row of curved, spine-like setae. Tarsal claw with one row of 12–14 denticles; distally pointed; with five stripes; subapical setae absent.

*Tergum* (Fig. 7c). Surface with irregular rows of U-shaped scale bases and micropores. Posterior margin of tergum IV with triangular spines, approx. as long as wide.

*Gills* (Fig. 7d). Present on segments I–VII. Margin with small denticles intercalating fine simple setae. Tracheae extending from main trunk to inner and outer margins. Gill I ca. 3/4 length of segment II. Gill IV as long as length of segments V and VI combined. Gill VII as long as length of segments VIII and 1/2 IX combined.

*Paraproct* (Fig. 7e). Distally not expanded, with 19–24 stout marginal spines. Surface scattered with U-shaped scale bases, fine, simple setae and micropores. Cercotractor with numerous small marginal spines.

##### Etymology.

Refers to the island Sumba, where the specimens were collected.

##### Distribution.

Indonesia: Sumba.

##### Biological aspects.

The specimens were collected in streams at an altitude of 400 m and 470 m and in waterholes at an altitude of 150 m.

##### Type-material.

**Holotype.** Larva (on slide, GBIFCH 00657749), Indonesia, Sumba, Waikelo, stream, 400 m, 27.IX.2011, 09°35.75'S, 119°20.42'E, M. Balke leg. (SUA04). Temporary deposited in MZL before definitely housed in MZB. **Paratypes.** 1 larva (on slide, GBIFCH 00592193, deposited in MZL), same data as holotype; 3 larvae (2 on slides, GBIFCH 00592191, GBIFCH 00592192, deposited in MZL; 1 in alcohol, GBIFCH 00515346, deposited in ZSM), Indonesia, Sumba, forest stream, 470 m, 27.IX.2011, 09°38.62'S, 119°40.93'E, M. Balke leg. (SUA07); 1 larva (on slide, GBIFCH 00235825, deposited in MZL), Indonesia, Sumba, Waitabula env., waterholes nr. limestone well, 150 m, 26.IX.2011, 09°26.06'S, 119°18.53'E, M. Balke leg. (SUA01).

### *Labiobaetis difficilis* group of species

With the following combination of characters: A) dorsal surface of labrum with submarginal arc of feathered setae; B) labial palp segment II with large, thumb-like distomedial protuberance; C) seven pairs of gills; D) paraproct not expanded distally; E) hindwing pads absent.

#### 4. Labiobaetis difficilis

Taxon classificationAnimaliaEphemeropteraBaetidae

(Müller-Liebenau, 1984)

2F0A4A03-1F2D-511A-B3F3-EC3C06769C6A

Figures 8 , 53b

##### Diagnosis.

**Larva.** Following combination of characters: A) dorsal surface of labrum with submarginal arc of 1 + 7–8 feathered setae; B) labial palp segment II with thumb-like distomedial protuberance, segment III oblong, apically slightly truncate; C) maxillary palp approx. as long as galea-lacinia; D) fore femur rather broad, length 3.4× maximum width, dorsal margin with a row of ca. ten curved, spine-like setae; E) fore claw with 11–12 denticles; F) paraproct distally not expanded, with 30–35 stout marginal spines.

##### Examined material.

**Paratype.** 1 larva (on slide, no. 36), W. Malaysia, Gombak River, 4 ½ miles N of Kuala Lumpur, 6.II.[19]69, Bishop leg.

**Figure 8.**
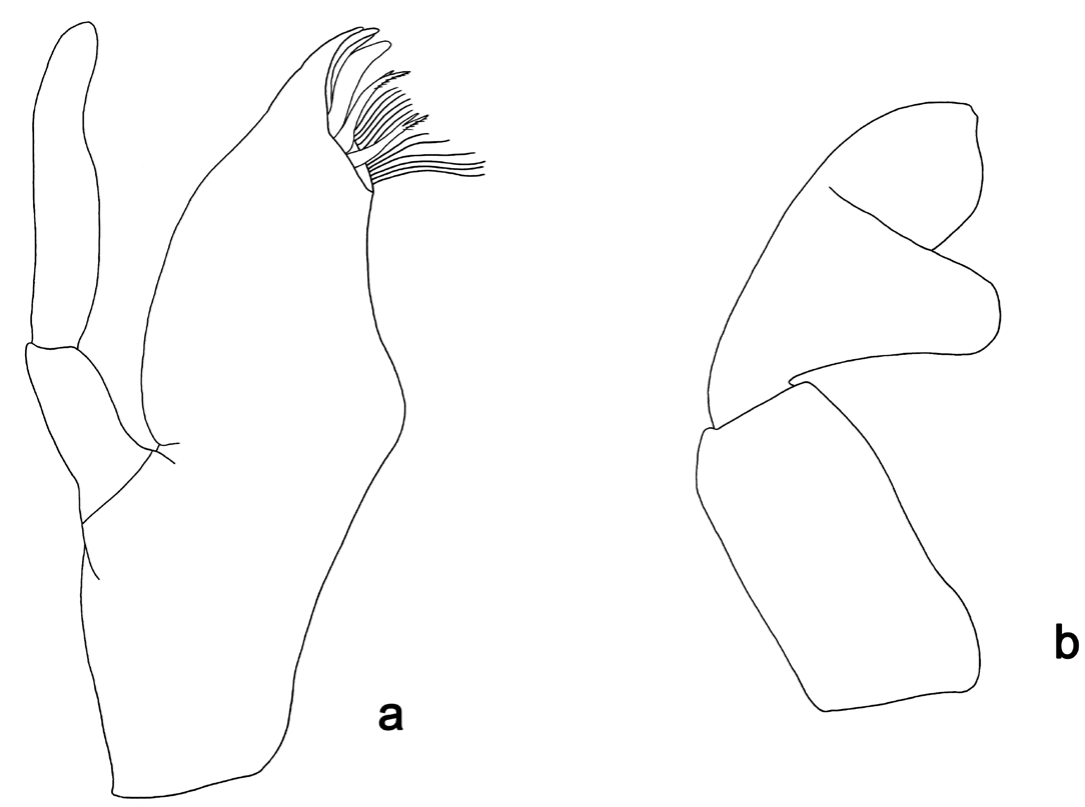
*Labiobaetis difficilis*, larva morphology: **a** Maxilla **b** Labial palp.

#### 5. Labiobaetis roulade sp. nov.

Taxon classificationAnimaliaEphemeropteraBaetidae

7BA57CE8-E1BF-50FD-A5B8-74973727F170

http://zoobank.org/7C7F4A79-E767-4EEE-8ADF-FE2CB67F0309

**Figure 9.**
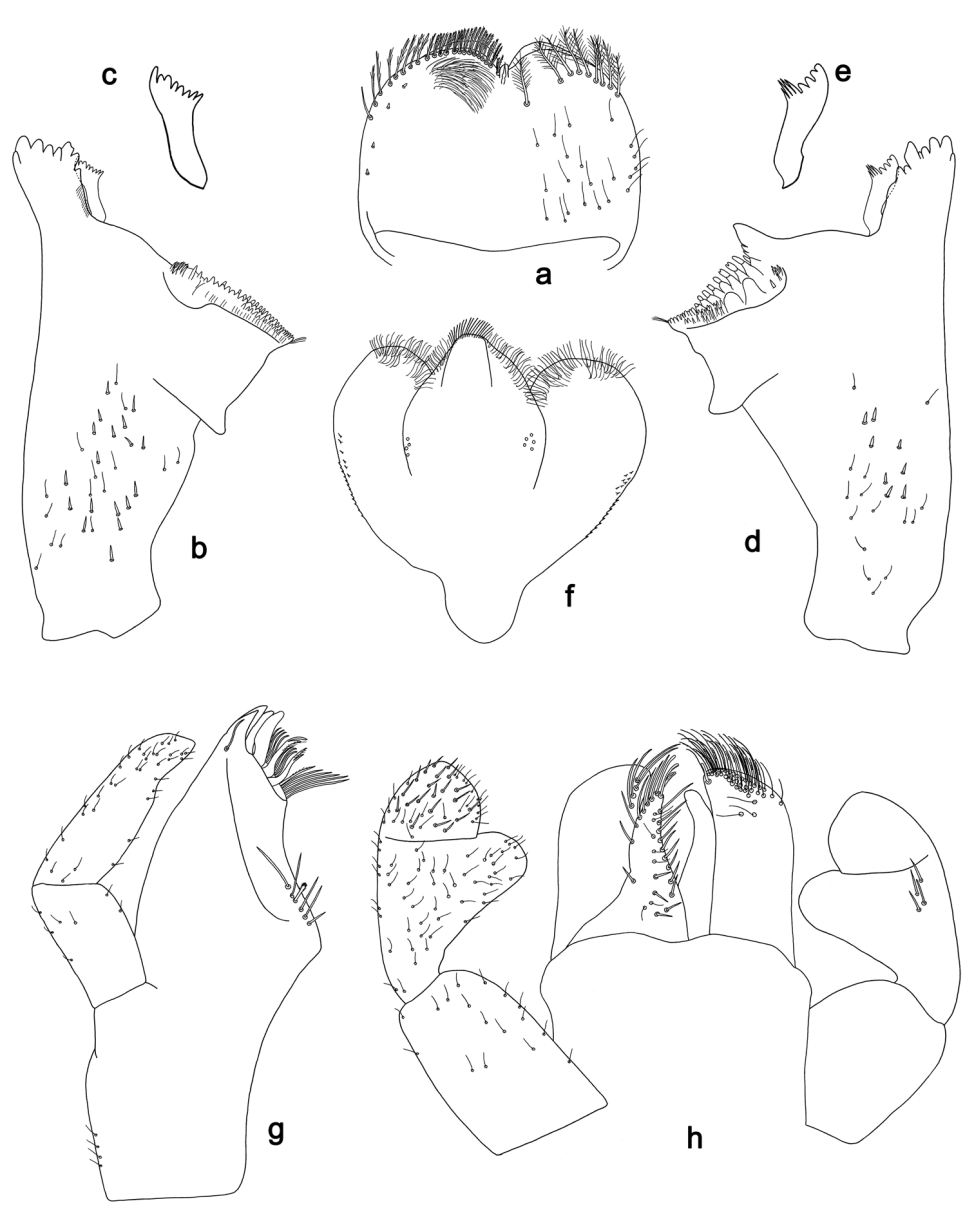
*Labiobaetis roulade* sp. nov., larva morphology: **a** Labrum **b** Right mandible **c** Right prostheca **d** Left mandible **e** Left prostheca **f**Hypopharynx**g** Maxilla **h** Labium.

**Figure 10.**
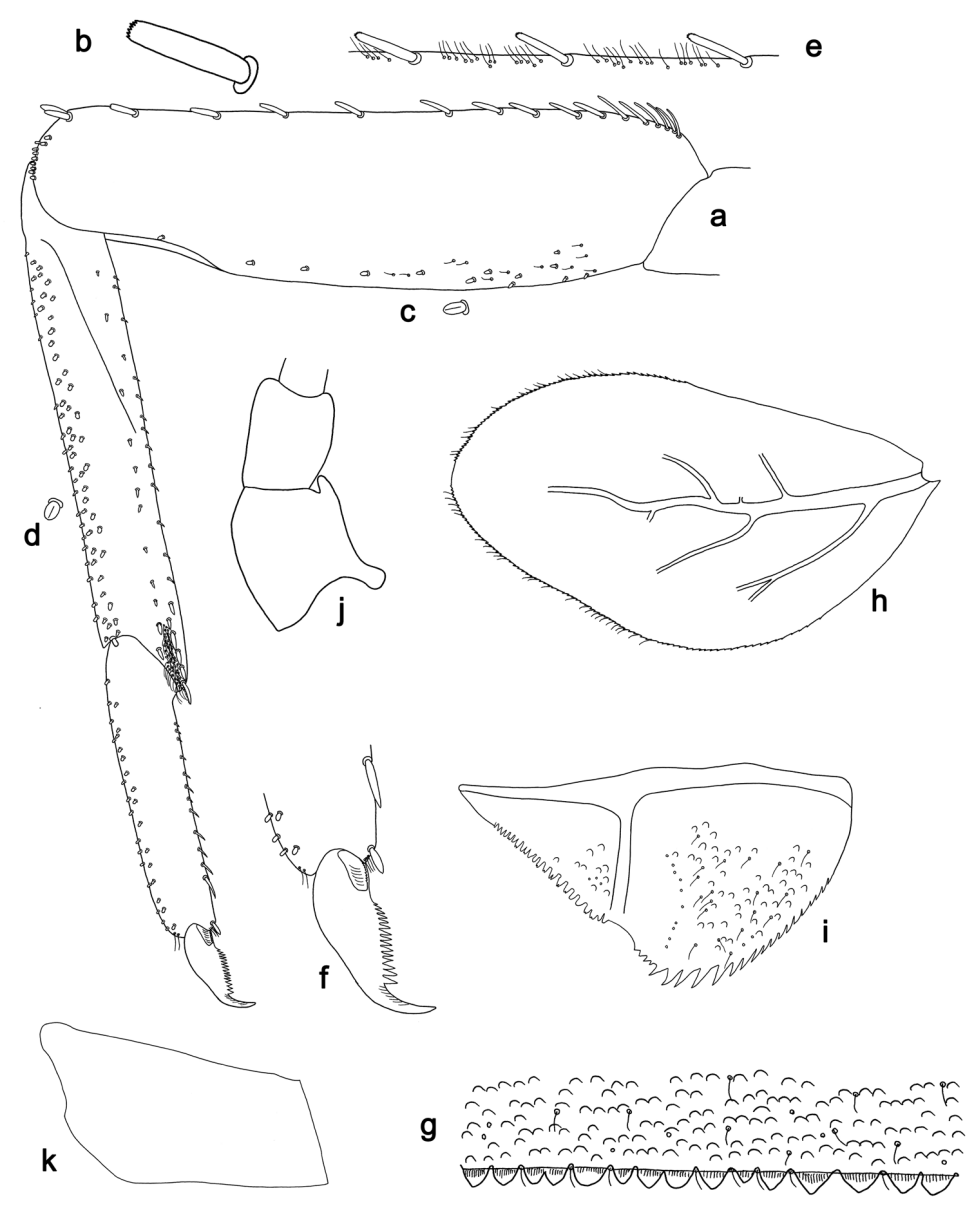
*Labiobaetis roulade* sp. nov., larva morphology: **a** Foreleg **b** Femur dorsal seta **c** Femur ventral seta **d** Tibia dorsal seta **e** Femur dorsal margin **f** Fore claw **g** Tergum IV **h** Gill IV **i** Paraproct **j** Scape **k** Metanotum.

Figures 9 , 10 , 47d , 52c , 55a

##### Diagnosis.

**Larva.** Following combination of characters: A) dorsal surface of labrum with submarginal arc of 1 + 8–9 feathered setae; B) maxillary palp approx. as long as galea-lacinia; C) labial palp segment II with broad thumb-like distomedial protuberance, segment III conical; D) fore femur rather slender, length 3.7× maximum width, dorsal margin with ca. 15 spine-like setae, apically rounded and with minute serration; E) paraproct distally not expanded, with ca. 21 stout marginal spines.

##### Description.

**Larva** (Figs 9, 10, 47d, 52c). Body length 5.2–6.2 mm. Cerci ca. 1/2 of body length. Antenna approximately 2.5× as long as head length.

*Colouration*. Head, thorax, and abdomen dorsally brown, head and thorax with bright median, dorsal suture, thorax and abdomen with bright pattern as in Fig. 47d, forewing pads with bright striation. Head, thorax, and abdomen ventrally brown, legs transparent, femur ventrodistomedially and distally with brown spot and dorsally with brown streak along margin (Fig. 52c), caudal filaments transparent with brown band at 1/3 of cerci.

*Antenna* with scape and pedicel subcylindrical, with well-developed distolateral process at scape; flagellum with broad, apically blunt spines and fine, simple setae on apex of each segment.

*Labrum* (Fig. 9a). Rectangular, length 0.8× maximum width. Distal margin with medial emargination and a small process. Dorsally with medium, fine, simple setae scattered over surface; submarginal arc of setae composed of 1 + 8–9 feathered setae. Ventrally with marginal row of setae composed of lateral and anterolateral long, feathered setae and medial long, bifid, pectinate setae; ventral surface with four short, spine-like setae near lateral and anterolateral margin.

*Right mandible* (Fig. 9b, c). Incisors fused. Outer and inner sets of denticles with 4 + 4 denticles. Inner margin of innermost denticle with a row of thin setae. Prostheca robust, apically denticulate. Margin between prostheca and mola slightly convex. Tuft of setae at apex of mola present.

*Left mandible* (Fig. 9d, e). Incisors fused. Outer and inner sets of denticles with 4 + 3 denticles and one minute intermediate denticle. Prostheca robust, apically with small denticles and comb-shaped structure. Margin between prostheca and mola slightly convex. Subtriangular process long and slender, above level of area between prostheca and mola. Denticles of mola apically constricted. Tuft of setae at apex of mola present.

Both mandibles with lateral margins almost straight. Basal half with fine, simple setae and slightly lanceolate setae scattered over surface.

*Hypopharynx* (Fig. 9f). Lingua longer than superlingua. Lingua longer than broad; medial tuft of stout setae poorly developed; distal half laterally expanded. Superlingua rounded; lateral margin rounded; fine, long, simple setae along distal margin.

*Maxilla* (Fig. 9g). Galea-lacinia with one simple, robust apical seta under crown. Inner dorsal row of setae with three denti-setae, distal denti-seta tooth-like, middle and proximal denti-setae slender, bifid, and pectinate. Medially with one bipectinate, spine-like seta and five long, simple setae. Maxillary palp approx. as long as length of galea-lacinia; two segmented; palp segment II 1.6× length of segment I; setae on maxillary palp fine, simple, scattered over surface of segments I and II; apex of last segment slightly pointed, with excavation at inner distolateral margin.

*Labium* (Fig. 9h). Glossa basally broad, narrowing toward apex; shorter than paraglossa; inner margin with nine spine-like setae increasing in length distally; apex with two long and one medium, robust, pectinate setae; outer margin with seven spine-like setae increasing in length distally; ventral surface with short, fine, simple and short, spine-like setae. Paraglossa sub-rectangular, curved inward; apex rounded; with three rows of long, robust, distally pectinate setae in apical area and 2–3 medium, simple setae in anteromedial area; dorsally with a row of four long, spine-like setae near inner margin. Labial palp with segment I 0.8× length of segments II and III combined. Segment I ventrally with short, fine, simple setae. Segment II with broad thumb-like distomedial protuberance; distomedial protuberance 0.5× width of base of segment III; inner and outer margin with short, fine, simple setae; dorsally with a row of four long, spine-like, simple setae near outer margin. Segment III conical; apex slightly pointed; length 0.8× width; ventrally covered with short, spine-like, simple setae and short, fine, simple setae.

*Hind wing pads* absent.

*Foreleg* (Fig. 10a–f). Ratio of foreleg segments 1.3:1.0:0.6:0.2. *Femur*. Length ca. 4× maximum width. Dorsal margin with a row of ca. 15 curved, spine-like setae, apically rounded and with minute serration, and many fine, simple setae; length of setae 0.17× maximum width of femur. Apex rounded; with one pair of curved, spine-like setae, apically rounded and with minute serration, and some short, stout setae. Short, stout, lanceolate setae scattered along the ventral margin; femoral patch reduced to a few setae. *Tibia.* Dorsal margin with a row of short, lanceolate, apically rounded setae and many short, lanceolate, apically rounded setae scattered along the dorsal margin. Ventral margin with a row of short, curved, spine-like setae, on apex some spine-like, partly bipectinate setae and a tuft of fine, simple setae. Anterior surface scattered with stout, lanceolate setae. Patellotibial suture present on basal 1/2. *Tarsus.* Dorsal margin with a row of short, lanceolate, apically rounded setae and many short, lanceolate, apically rounded setae scattered along the dorsal margin. Ventral margin with a row of curved, spine-like setae. Tarsal claw with one row of 15 denticles; distally pointed; with 7–8 stripes; subapical setae absent.

*Tergum* (Fig. 10g). Surface with irregular rows of U-shaped scale bases and scattered fine, simple setae and micropores. Posterior margin of tergum IV with rounded or triangular spines, wider than long.

*Gills* (Fig. 10h). Present on segments I–VII. Margin with small denticles intercalating fine simple setae. Tracheae partly extending from main trunk towards outer and inner margins. Gill I ca. 2/3 length of segment II. Gill IV as long as length of segments V and 2/3 VI combined. Gill VII as long as length of segments VIII and 1/3 IX combined.

*Paraproct* (Fig. 10i). Distally not expanded, with ca. 21 stout marginal spines. Surface scattered with U-shaped scale bases, fine, simple setae and micropores. Cercotractor with numerous small marginal spines.

##### Etymology.

Dedicated to Jean-Marc Elouard, the collector of the specimens and well-known ephemerologist. The name is an anagram of Elouard.

##### Distribution.

Indonesia: Sumatra.

##### Biological aspects.

The specimens were collected at an altitude of 530 m.

##### Type-material.

**Holotype.** Larva (on slide, GBIFCH 00592234), Indonesia, Sumatra Barat, Payakumbuh, south of Payakumbuh, 00°16.55'S, 100°36.28'E, 530 m, 28.V.2010, J.-M. Elouard leg. Temporary deposited in MZL before definitely housed in MZB. **Paratypes.** 9 larvae (2 on slides, GBIFCH 00592235, GBIFCH 00592250, 4 in alcohol, GBIFCH 00515364, deposited in MZL; 3 in alcohol, GBIFCH 00515363, GBIFCH 00657757, GBIFCH 00657756, deposited in ZSM), same data as holotype.

#### 6. Labiobaetis weifangae sp. nov.

Taxon classificationAnimaliaEphemeropteraBaetidae

63F46433-626B-5A94-A2EA-ACB23C6EAD08

http://zoobank.org/19916328-4EF6-4798-9D8B-61B654813858

**Figure 11.**
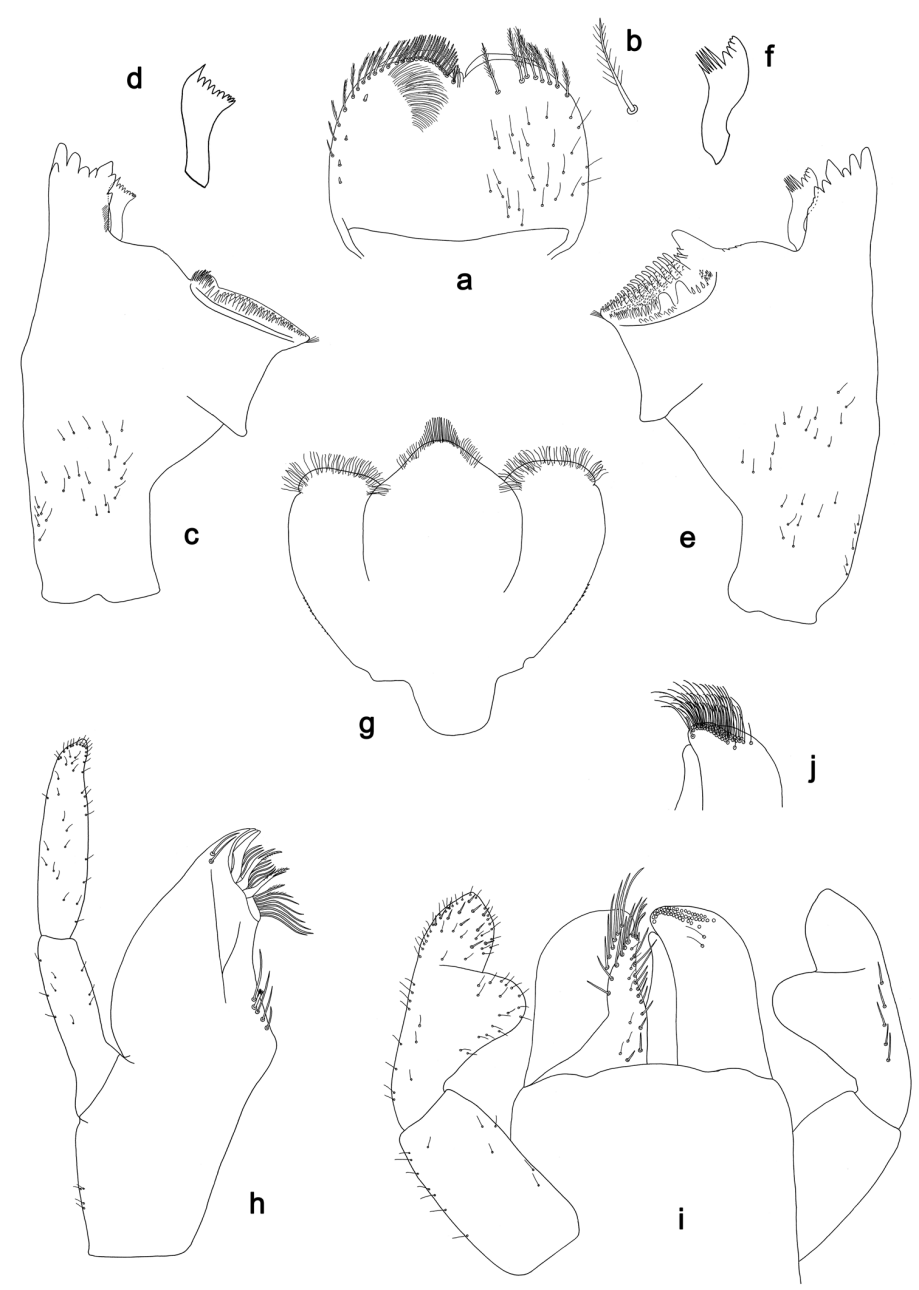
*Labiobaetis weifangae* sp. nov., larva morphology: **a** Labrum **b** Seta of the submarginal arc on the dorsal surface of the labrum **c** Right mandible **d** Right prostheca **e** Left mandible **f** Left prostheca **g**Hypopharynx**h** Maxilla **i** Labium **j** Apex of paraglossa.

**Figure 12.**
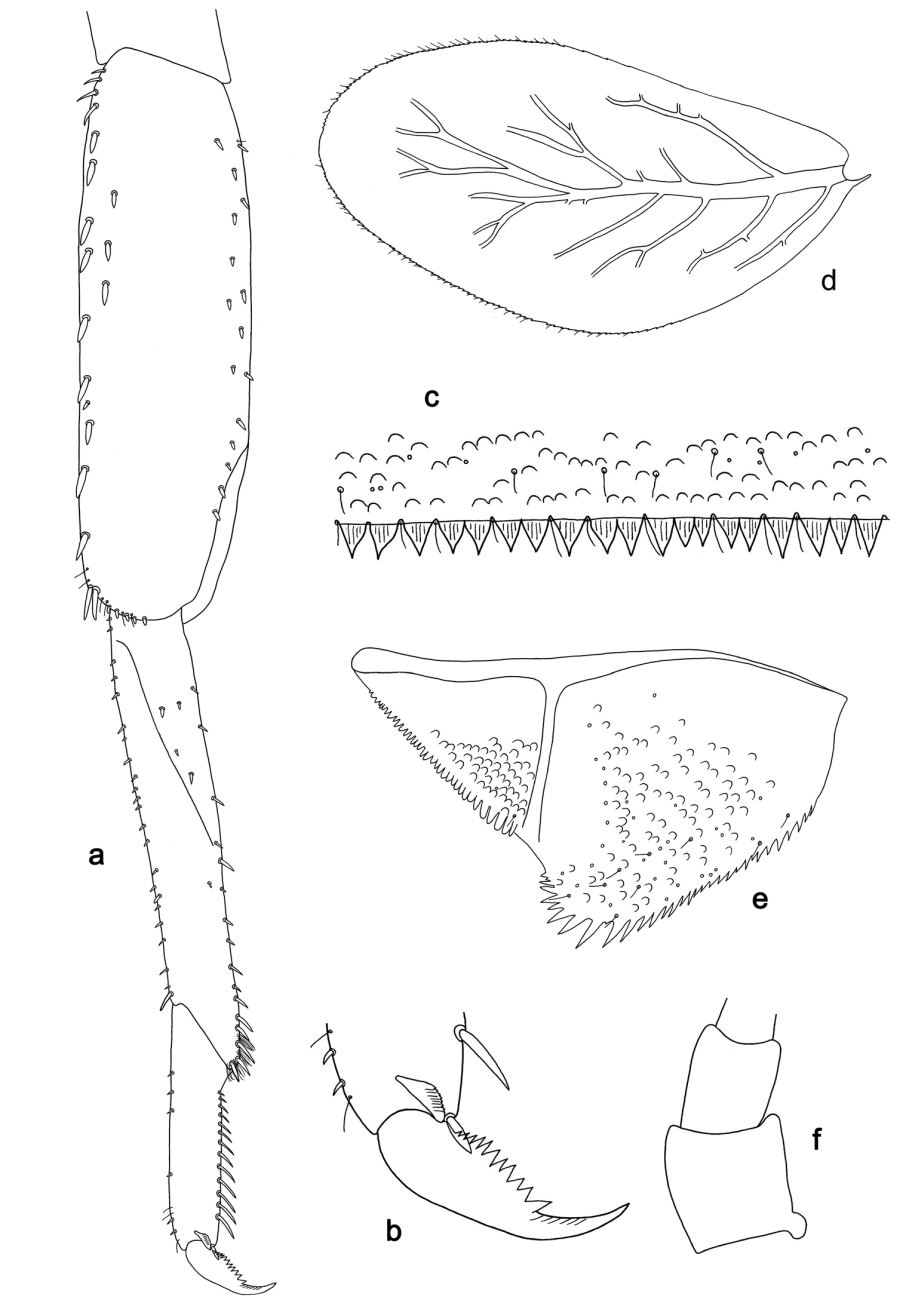
*Labiobaetis weifangae* sp. nov., larva morphology: **a** Foreleg **b** Fore claw **c** Tergum IV **d** Gill IV **e** Paraproct **f** Scape.

Figures 11 , 12 , 48a , 52d , 54b

##### Diagnosis.

**Larva.** Following combination of characters: A) dorsal surface of labrum with submarginal arc of 1 + 7 long, feathered setae; B) labial palp segment II with broad thumb-like distomedial protuberance; C) fore femur rather broad, length 3.3× maximum width, dorsal margin with a row of ca. 13 spine-like setae and some spine-like setae scattered along margin, D) spines at posterior margin of tergum IV triangular, longer than wide; E) paraproct distally not expanded, with 26–32 stout marginal spines.

##### Description.

**Larva** (Figs 11, 12, 48a, 52d). Body length 8.2 mm.

*Colouration.* Head, thorax, and abdomen dorsally dark brown, head and thorax with bright median, dorsal suture, thorax and abdomen with bright pattern as in Fig. 48a, forewing pads light brown with darker striation. Head, thorax and abdominal segment I ventrally transparent, abdominal segments II–X ventrally brown, femur transparent, with a ventroproxomedial and dorsodistomedial brown streak and an apical brown spot, tibia and tarsus light brown (Fig. 52d), caudal filaments light brown, with a dark brown band at 1/3 of cerci.

*Antenna* with scape and pedicel subcylindrical, with poorly developed distolateral process at scape; flagellum with lanceolate spines and fine, simple setae on apex of each segment.

*Labrum* (Fig. 11a, b). Rectangular, length 0.7× maximum width. Distal margin with medial emargination and a small process. Dorsally with medium, fine, simple setae scattered over surface; submarginal arc of setae composed of 1 + 7 long, feathered setae. Ventrally with marginal row of setae composed of lateral and anterolateral long, feathered setae and medial long, bifid, pectinate setae; ventral surface with four short, spine-like setae near lateral and anterolateral margin.

*Right mandible* (Fig. 11c, d). Incisors fused. Outer and inner sets of denticles with 4 + 3 denticles and one minute intermediate denticle. Inner margin of innermost denticle with a row of thin setae. Prostheca robust, apically denticulate. Margin between prostheca and mola slightly convex. Tuft of setae at apex of mola present.

*Left mandible* (Fig. 11e, f). Incisors fused. Outer and inner sets of denticles with 3 + 3 denticles. Prostheca robust, apically with small denticles and comb-shaped structure. Margin between prostheca and mola straight, with minute denticles towards subtriangular process. Subtriangular process long and slender, above level of area between prostheca and mola. Denticles of mola apically constricted. Tuft of setae at apex of mola present.

Both mandibles with lateral margins almost straight. Basal half with fine, simple setae scattered over dorsal surface.

*Hypopharynx* (Fig. 11g). Lingua longer than superlingua. Lingua approx. as broad as long; medial tuft of stout setae poorly developed; distal half not expanded. Superlingua rounded; lateral margin rounded; fine, long, simple setae along distal margin.

*Maxilla* (Fig. 11h). Galea-lacinia with two simple, robust apical setae under crown. Inner dorsal row of setae with three denti-setae, distal denti-seta tooth-like, middle and proximal denti-setae slender, bifid and pectinate. Medially with one bipectinate, spine-like seta and four long, simple setae. Maxillary palp 1.4× as long as length of galea-lacinia; two segmented; palp segment II 1.3× length of segment I; setae on maxillary palp fine, simple, scattered over surface of segments I and II; apex of last segment constricted, with excavation at inner distolateral margin.

*Labium* (Fig. 11i, j). Glossa basally broad, narrowing toward apex; shorter than paraglossa; inner margin with ten spine-like setae increasing in length distally; apex with two long and one medium, robust, pectinate setae and one short, robust seta; outer margin with six long spine-like setae increasing in length distally; ventral surface with short, fine, simple and short, spine-like setae. Paraglossa sub-rectangular, curved inward; apex rounded; with three rows of long, robust, distally pectinate setae in apical area and two medium, simple setae in anteromedial area; dorsally with a row of six long, spine-like setae near inner margin. Labial palp with segment I 0.9× length of segments II and III combined. Segment I ventrally with short, fine, simple setae. Segment II with broad thumb-like distomedial protuberance; distomedial protuberance 0.7× width of base of segment III; inner and outer margin with short, fine, simple setae; dorsally with a row of five medium, spine-like, simple setae near outer margin. Segment III conical; apex slightly pointed; length 1.1× width; ventrally covered with short, spine-like, simple setae and short, fine, simple setae.

*Hind wing pads* absent.

*Foreleg* (Fig. 12a, b). Ratio of foreleg segments 1.3:1.0:0.6:0.2. *Femur*. Length ca. 3× maximum width. Dorsal margin with a row of ca. 13 spine-like setae and some spine-like setae scattered along margin; length of setae 0.18× maximum width of femur. Apex rounded; with one pair of spine-like setae and some short, stout setae. Many stout, lanceolate setae scattered along the ventral margin; femoral patch absent. *Tibia.* Dorsal margin with a row of short, curved, spine-like setae; on apex one longer, curved, spine-like seta. Ventral margin with a row of curved, spine-like setae, on apex with several stout, partly bipectinate, spine-like setae and a tuft of long, fine, simple setae. Anterior surface scattered with stout, lanceolate setae. Patellotibial suture present on basal 1/2. *Tarsus.* Dorsal margin with a row of short, spine-like setae and fine, simple setae. Ventral margin with a row of curved, spine-like setae. Tarsal claw with one row of 11–13 denticles; distally pointed; with six stripes; subapical setae absent.

*Tergum* (Fig. 12c). Surface with irregular rows of U-shaped scale bases and scattered fine, simple setae and micropores. Posterior margin of tergum IV with triangular spines, longer than wide.

*Gills* (Fig. 12d). Present on segments I–VII. Margin with small denticles intercalating fine simple setae. Tracheae extending from main trunk to inner and outer margins. Gill I a little longer than segment II. Gill IV as long as length of segments V and 3/4 VI combined. Gill VII as long as length of segments VIII and 1/2 IX combined.

*Paraproct* (Fig. 12e). Distally not expanded, with 26–32 stout marginal spines. Surface scattered with U-shaped scale bases, fine, simple setae and micropores. Cercotractor with numerous small marginal spines.

##### Etymology.

Dedicated to Weifang Shi (Guizhou Center for Disease Control and Prevention, Guiyang, China), a renowned specialist of Baetidae.

##### Distribution.

Indonesia: Sumba and Sumbawa.

##### Biological aspects.

The specimens were collected in rest pools and waterholes at altitudes of 70 m and 150 m.

##### Type-material.

**Holotype.** Larva (on slide, GBIFCH 00592228), Indonesia, Sumbawa, Dompu-Huu, restpools, 70 m, 14.IX.2011, 08°37.72'S, 118°29.62'E, M. Balke leg. (SUMB03). Temporary deposited in MZL before definitely housed in MZB. **Paratypes.** 7 larvae (1 on slide, GBIFCH 00592230, 4 in alcohol, GBIFCH 00515350, deposited in MZL; 2 in alcohol, GBIFCH 00657740, GBIFCH 00657743, deposited in ZSM), same data as holotype; 1 larva (on slide, GBIFCH 00592229, deposited in MZL), Indonesia, Sumba, Waitabula env., waterholes nr. limestone well, 150 m, 26.IX.2011, 09°26.06'S, 119°18.53'E, M. Balke leg. (SUA01).

### *Labiobaetis sumigarensis* group of species

With the following combination of characters: A) dorsal surface of labrum with submarginal arc of clavate, apically smooth setae; B) labial palp segment II with large, lobed or thumb-like distomedial protuberance, outer margin of protuberance predominantly concave (*L. sumigarensis* with hook-like modification of the lobed protuberance); C) left mandible without setae at apex of mola, with minute denticles between prostheca and mola; D) six pairs of gills; E) hindwing pads absent; F) distolateral process at scape poorly developed or absent; G) colour of larvae dorsally uniform brown.

#### 7. Labiobaetis diffundus

Taxon classificationAnimaliaEphemeropteraBaetidae

(Müller-Liebenau, 1984)

0983C514-DEB6-5844-ABDD-D54C37BABE56

Figures 13 , 53b

##### Diagnosis.

**Larva.** Following combination of characters. A) dorsal surface of labrum with submarginal arc of ca. 16 clavate setae; B) labial palp segment II with a large, lobed distomedial protuberance, segment III slightly pentagonal, apically slightly pointed; C) left mandible without setae at apex of mola; D) fore femur rather slender, length ca. 4× maximum width, dorsal margin with a row of 10–13 curved, spine-like setae, femoral patch well developed; E) tarsus with pectinate setae at ventral margin (difficult to see), claw with ca. ten denticles; F) paraproct distally not expanded, with ca. 35 stout marginal spines.

##### Examined material.

**Paratypes.** 1 larva (on slide, no. 21), W. Malaysia, Gombak River, 4 ½ miles N. of Kuala Lumpur, 6.II.[19]69, Bishop leg.; 1 larva (on slide, no. 41), W. Malaysia, trib. of Gombak River, 16 ½ miles N. of Kuala Lumpur, 14.XI.[19]68, Bishop leg.

**Figure 13.**
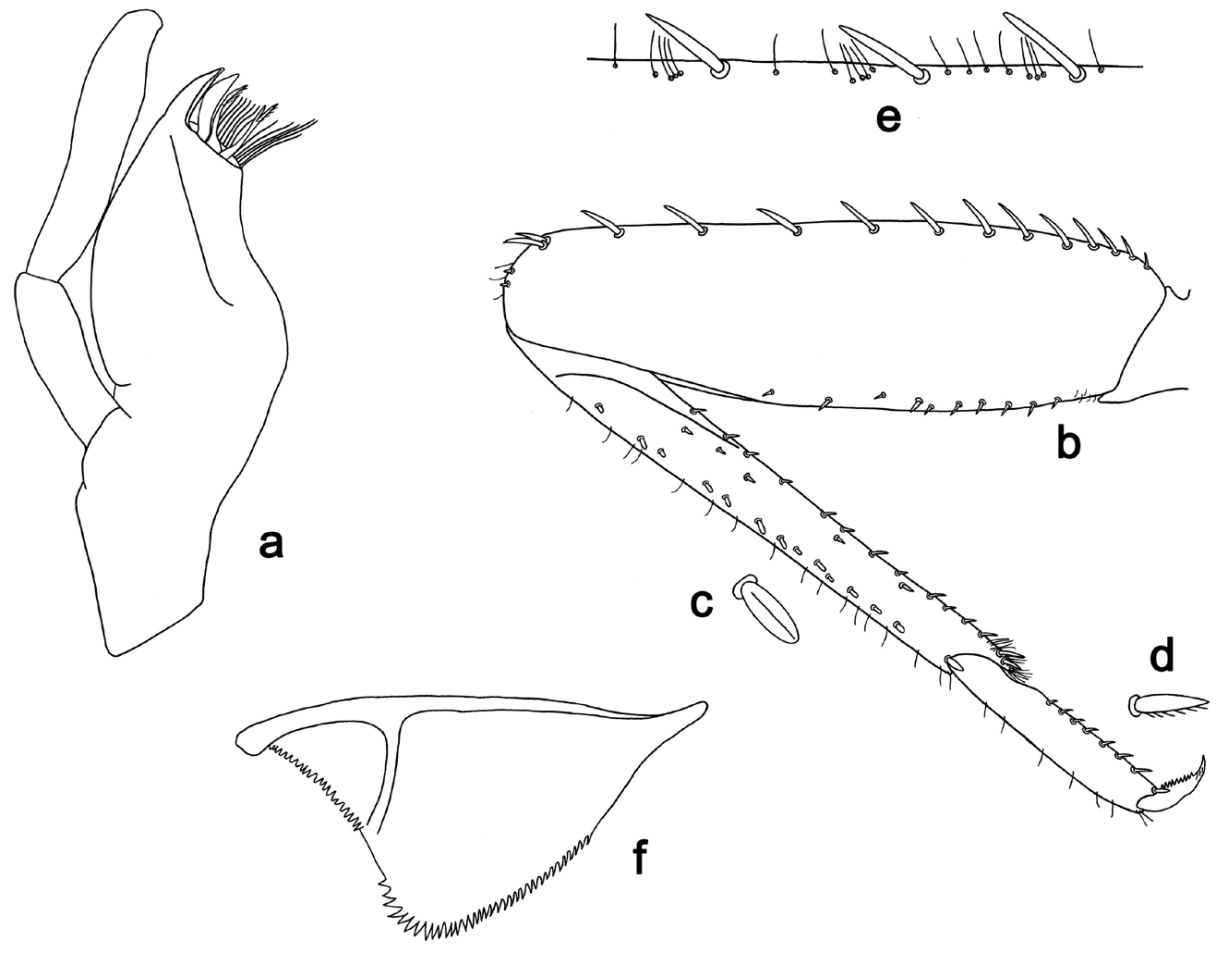
*Labiobaetis diffundus*, larva morphology: **a** Maxilla **b** Foreleg **c** Fore tibia dorsal seta **d** Fore tarsus ventral seta **e** Femur dorsal margin **f** Paraproct.

#### 8. Labiobaetis molawinensis

Taxon classificationAnimaliaEphemeropteraBaetidae

(Müller-Liebenau, 1982)

CFD1A54B-A28B-5F6C-BEF9-01756E88E0F4

Figures 14 , 53b

##### Diagnosis.

**Larva.** Following combination of characters: A) dorsal surface of labrum with submarginal arc of ca. 15 clavate setae; B) labial palp segment II with a large, lobed distomedial protuberance, segment III slightly pentagonal, apically slightly truncate; C) left mandible without setae at apex of mola; D) fore femur rather slender, length 3.6× maximum width, dorsal margin with a row of ca. ten curved, spine-like setae; E) tarsal claw with ca. eleven denticles; F) paraproct distally not expanded, with > 40 stout marginal spines.

##### Examined material.

**Paratype.** 1 larva (on slide), Philippines, Coll. Pescador, rapids Molawin creek, college, Laguna, 28.VII.1977, C.R. Realon leg.

**Figure 14.**
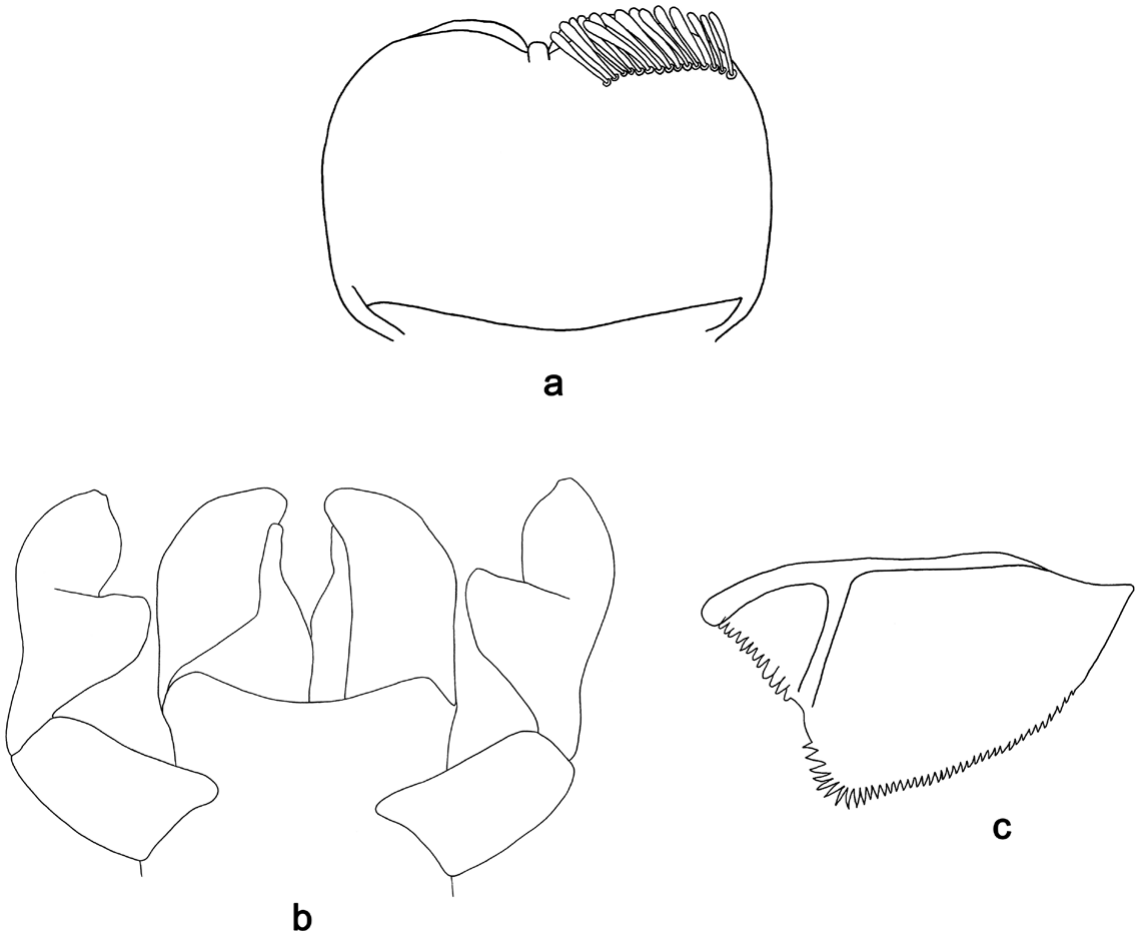
*Labiobaetis molawinensis*, larva morphology: **a** Labrum **b** Labium **c** Paraproct.

#### 9. Labiobaetis sumigarensis

Taxon classificationAnimaliaEphemeropteraBaetidae

(Müller-Liebenau, 1982)

957A5546-17A2-59E7-94F1-511E3922174B

Figures 15 , 53b

##### Diagnosis.

**Larva.** Following combination of characters: A) dorsal surface of labrum with submarginal arc of ca. 26 clavate setae; B) labial palp segment II with a hook-like distomedial protuberance, segment III slightly pentagonal, apically slightly pointed; C) left mandible without setae at apex of mola; D) fore femur rather broad, length 3.4× maximum width, dorsal margin with ca. 15 curved, spine-like setae; E) tarsal claw with ca. ten denticles; F) paraproct slightly expanded, with 35–39 stout marginal spines, some with split tips.

##### Examined material.

**Holotype.** 1 larva (on slide), Philippines, Mountain Prov., Sumigar stream, Sumigar, Banaue, 3.X1967, M.L. Pescador leg.

**Figure 15.**
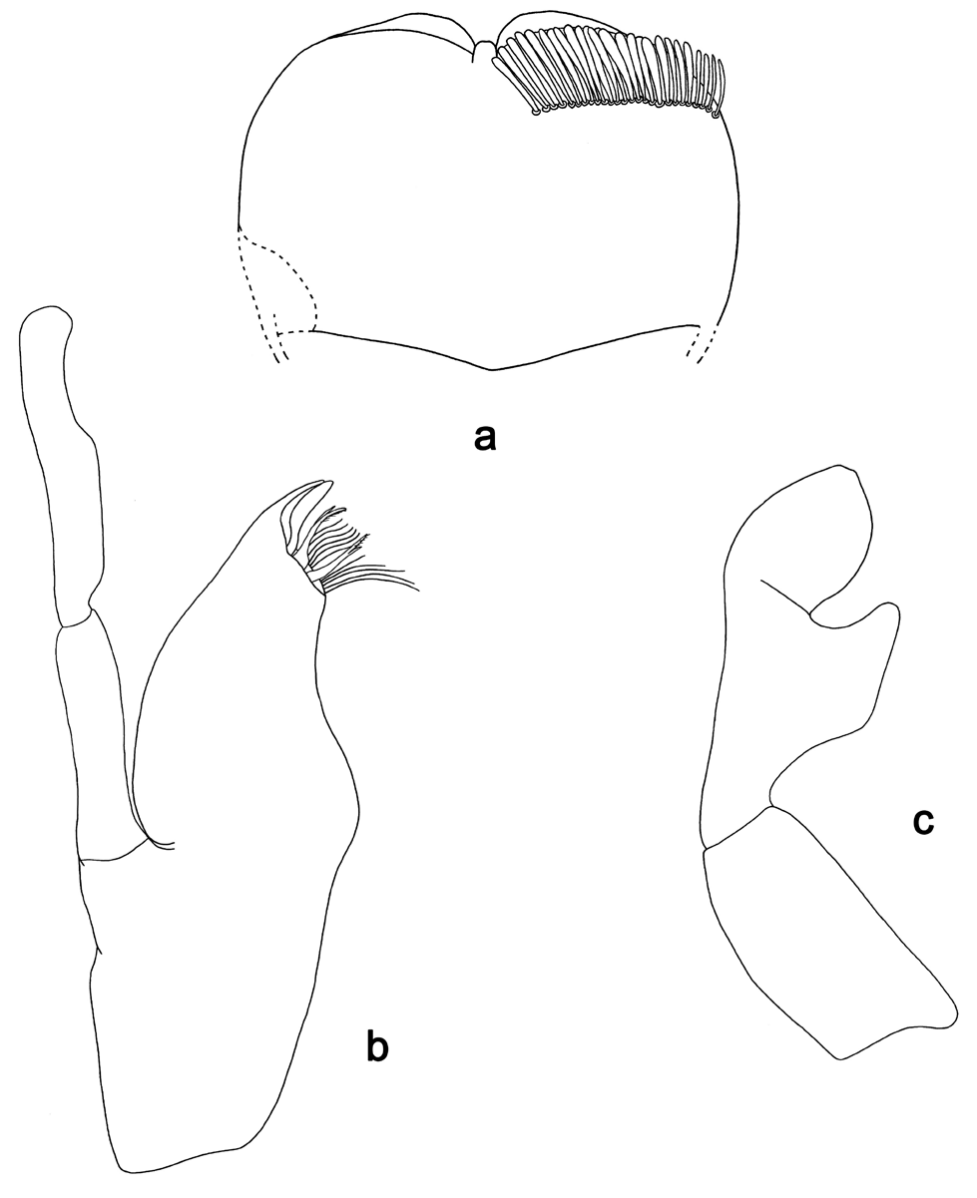
*Labiobaetis sumigarensis*, larva morphology: **a** Labrum **b** Maxilla **c** Labial palp.

#### 10. Labiobaetis itineris sp. nov.

Taxon classificationAnimaliaEphemeropteraBaetidae

102E8928-30F1-5B06-803C-890B00E0241E

http://zoobank.org/BCE97FC1-A22F-4AE2-9416-286329F2A130

**Figure 16.**
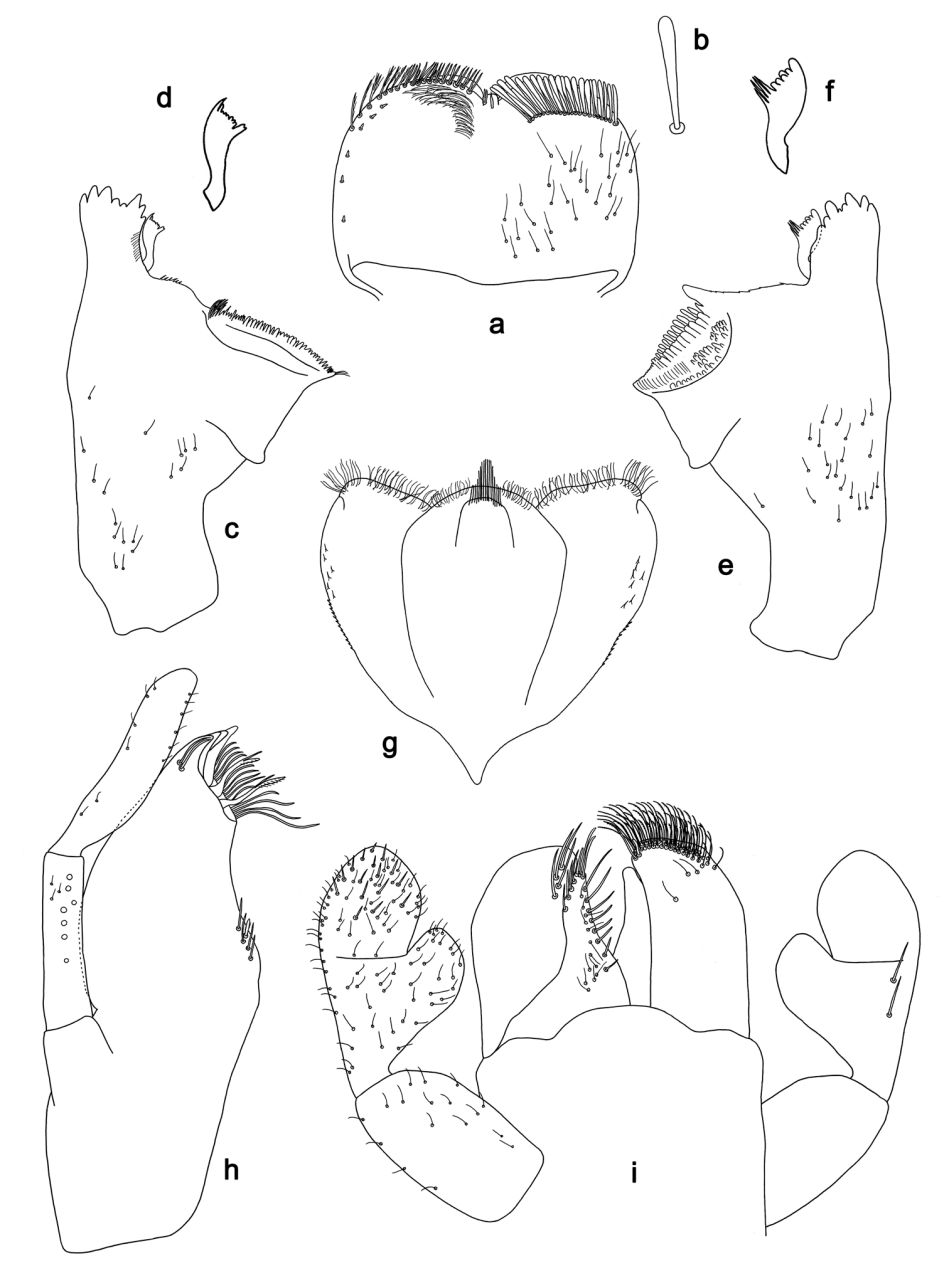
*Labiobaetis itineris* sp. nov., larva morphology: **a** Labrum **b** Seta of the submarginal arc on the dorsal surface of the labrum **c** Right mandible **d** Right prostheca **e** Left mandible **f** Left prostheca **g**Hypopharynx**h** Maxilla **i** Labium.

**Figure 17.**
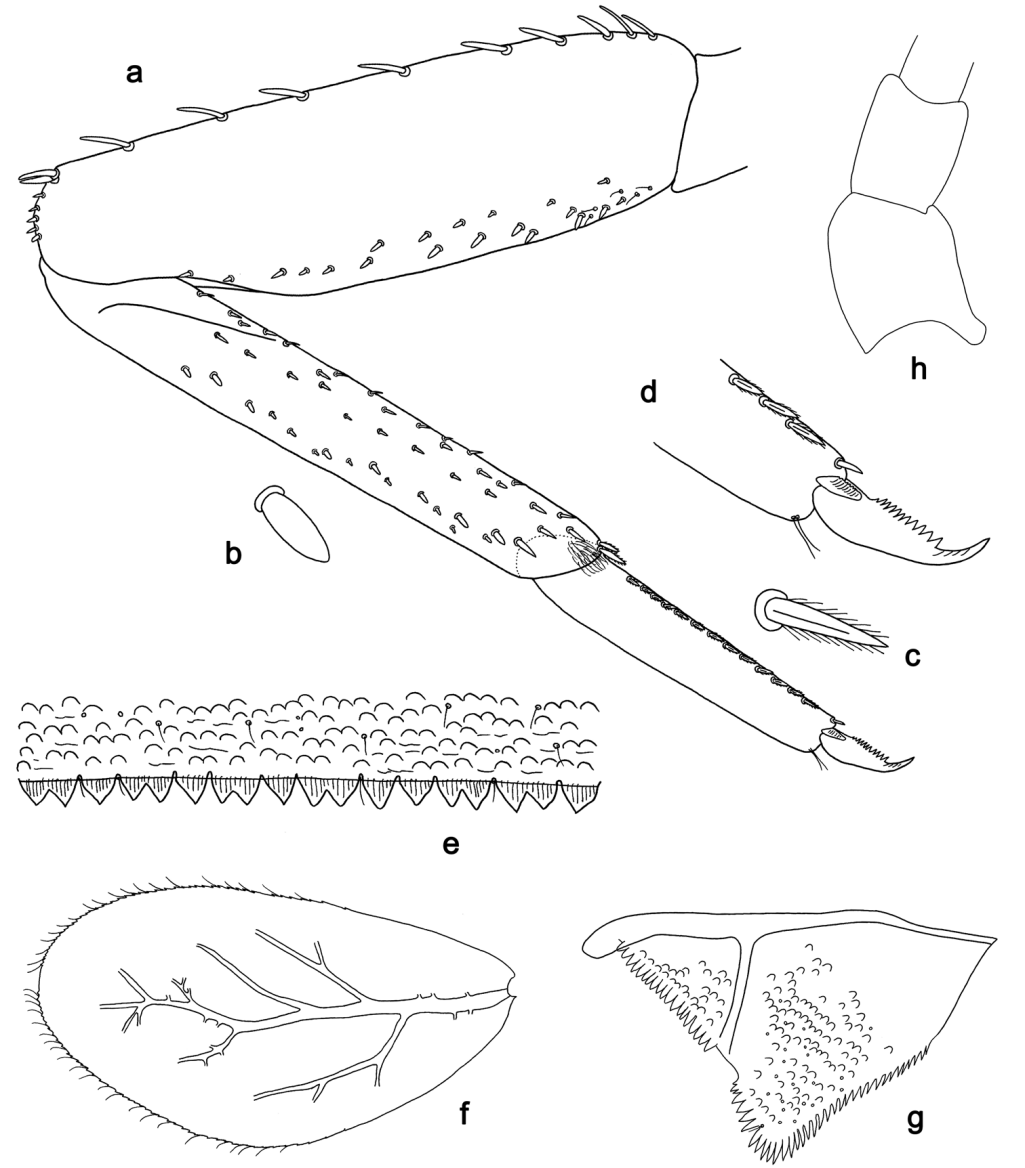
*Labiobaetis itineris* sp. nov., larva morphology: **a** Foreleg **b** Fore tibia dorsal seta **c** Fore tarsus ventral seta **d** Fore claw **e** Tergum IV **f** Gill IV **g** Paraproct **h** Scape.

Figures 16 , 17 , 48b , 54b

##### Diagnosis.

**Larva.** Following combination of characters: A) dorsal surface of labrum with submarginal arc of 20–23 clavate setae; B) labial palp segment II with a large, thumb-like distomedial protuberance, segment III oblong; C) left mandible without setae at apex of mola; D) fore femur rather broad, length 3.4× maximum width, dorsal margin with 9–12 curved, spine-like setae; E) tarsus ventrally with a row of stout, bipectinate setae; F) paraproct distally expanded, with ca. 40 stout marginal spines.

##### Description.

**Larva** (Figs 16, 17, 48b). Body length 5.8 mm.

*Colouration.* Head, thorax, and abdomen dorsally brown, head, and thorax with bright median, dorsal suture, thorax with pattern as in Fig. 48b, forewing pads with bright striation. Head, thorax, and abdomen ventrally light brown, caudal filaments light brown.

*Antenna* with scape and pedicel subcylindrical, with poorly developed distolateral process at scape; flagellum with lanceolate spines and fine, simple setae on apex of each segment.

*Labrum* (Fig. 16a, b). Rectangular, length 0.7× maximum width. Distal margin with medial emargination and a small process. Dorsally with medium, fine, simple setae scattered over surface; submarginal arc of setae composed of 20–23 long, clavate setae. Ventrally with marginal row of setae composed of anterolateral long, feathered setae and medial long, bifid setae; ventral surface with six short, spine-like setae near lateral and anterolateral margin.

*Right mandible* (Fig. 16c, d). Incisors fused. Outer and inner sets of denticles with 4 + 3 denticles and one minute intermediate denticle. Inner margin of innermost denticle with a row of thin setae. Prostheca robust, apically denticulate. Margin between prostheca and mola slightly convex, with minute setae. Tuft of setae at apex of mola present.

*Left mandible* (Fig. 16e, f). Incisors fused. Outer and inner sets of denticles with 4 + 3 denticles and one minute intermediate denticle. Prostheca robust, apically with small denticles and comb-shaped structure. Margin between prostheca and mola straight, with minute denticles towards subtriangular process. Subtriangular process long and slender, above level of area between prostheca and mola. Denticles of mola apically constricted. Tuft of setae at apex of mola absent.

Both mandibles with lateral margins almost straight. Basal half with fine, simple setae scattered over dorsal surface.

*Hypopharynx* (Fig. 16g). Lingua approx. as long as superlingua. Lingua longer than broad; medial tuft of stout setae well developed; distal half laterally expanded. Superlingua straight; lateral margin rounded; fine, long, simple setae along distal margin.

*Maxilla* (Fig. 16h). Galea-lacinia with two simple, robust apical setae under crown. Inner dorsal row of setae with three denti-setae, distal denti-seta tooth-like, middle and proximal denti-setae slender, bifid and pectinate. Medially with one bipectinate, spine-like seta and 3–4 medium, simple setae. Maxillary palp 1.2× as long as length of galea-lacinia; two segmented; palp segment II 1.3× length of segment I; setae on maxillary palp fine, simple, scattered over surface of segments I and II; apex of last segment rounded, with slight excavation at inner distolateral margin.

*Labium* (Fig. 16i). Glossa basally broad, narrowing toward apex; shorter than paraglossa; inner margin with eight spine-like setae increasing in length distally; apex with two long and one medium, robust setae; outer margin with 4–6 long, spine-like setae increasing in length distally; ventral surface with short, fine, simple, scattered setae. Paraglossa sub-rectangular, curved inward; apex rounded; with three rows of long, robust, distally pectinate setae in apical area and three medium, simple setae in anteromedial area; dorsally with a row of four or five long, spine-like setae near inner margin. Labial palp with segment I 0.8× length of segments II and III combined. Segment I ventrally with short, fine, simple setae. Segment II with broad thumb-like distomedial protuberance; distomedial protuberance 0.6× width of base of segment III; inner and outer margin with short, fine, simple setae; dorsally with two long, spine-like, simple setae near outer margin. Segment III oblong; apex rounded; length 1.1× width; ventrally covered with short, spine-like, simple setae and short, fine, simple setae.

*Hind wing pads* absent.

*Foreleg* (Fig. 17a, b, c, d). Ratio of foreleg segments 1.2:1.0:0.5:0.2. *Femur*. Length ca. 3× maximum width. Dorsal margin with a row of 9–12 curved, spine-like setae; length of setae 0.23× maximum width of femur. Apex rounded; with one pair of curved, spine-like setae and some short, stout setae. Many stout, lanceolate setae scattered along the ventral margin; femoral patch poorly developed. *Tibia.* Dorsal margin with a row of stout, lanceolate setae. Ventral margin with a row of short, curved, spine-like setae, on apex two bipectinate, spine-like setae and a tuft of fine, simple setae. Anterior surface scattered with stout, lanceolate setae. Patellotibial suture present on basal 1/3. *Tarsus.* Dorsal margin bare. Ventral margin with a row of spine-like, bipectinate setae. Tarsal claw with one row of eleven denticles; distally pointed; with four stripes; subapical setae absent.

*Tergum* (Fig. 17e). Surface with irregular rows of U-shaped scale bases and scattered fine, simple setae and micropores. Posterior margin of tergum IV with triangular spines, wider than long.

*Gills* (Fig. 17f). Present on segments II–VII. Margin with small denticles intercalating fine simple setae. Tracheae extending from main trunk to inner and outer margins. Gill IV as long as length of segments V and 1/2 VI combined. Gill VII as long as length of segments VIII and 1/4 IX combined.

*Paraproct* (Fig. 17g). Distally expanded, with 35–43 stout marginal spines. Surface scattered with U-shaped scale bases and micropores. Cercotractor with numerous small marginal spines.

##### Etymology.

Refers to travel activities, as the islands Sumbawa and especially Bali are well known for tourism.

##### Distribution.

Indonesia: Sumbawa and Bali.

##### Biological aspects.

The specimens were collected at altitudes of 195 m and 860 m.

##### Type-material.

**Holotype.** Larva (on slide, GBIFCH 00592225), Indonesia, Bali, Ubud, Sayan, Ayung River, 194 m, 20.IX.2011, 08°29.98'S, 115°14.59'E, M. Balke leg. (BLI015). Temporary deposited in MZL before definitely housed in MZB. **Paratypes.** 2 larvae (on slides, GBIFCH 00592226, GBIFCH 00592227, deposited in MZL), Indonesia, Sumbawa, Batu Dulang, 10 mins to Tepal, forest stream, 860 m, 16.IX.2011, 08°35.87'S, 117°16.68'E, M. Balke leg. (SUMB08).

#### 11. Labiobaetis lubu sp. nov.

Taxon classificationAnimaliaEphemeropteraBaetidae

DF771126-972A-5998-B124-1F462981F489

http://zoobank.org/68F1579E-07BC-4465-A531-2B4110EEF75E

**Figure 18.**
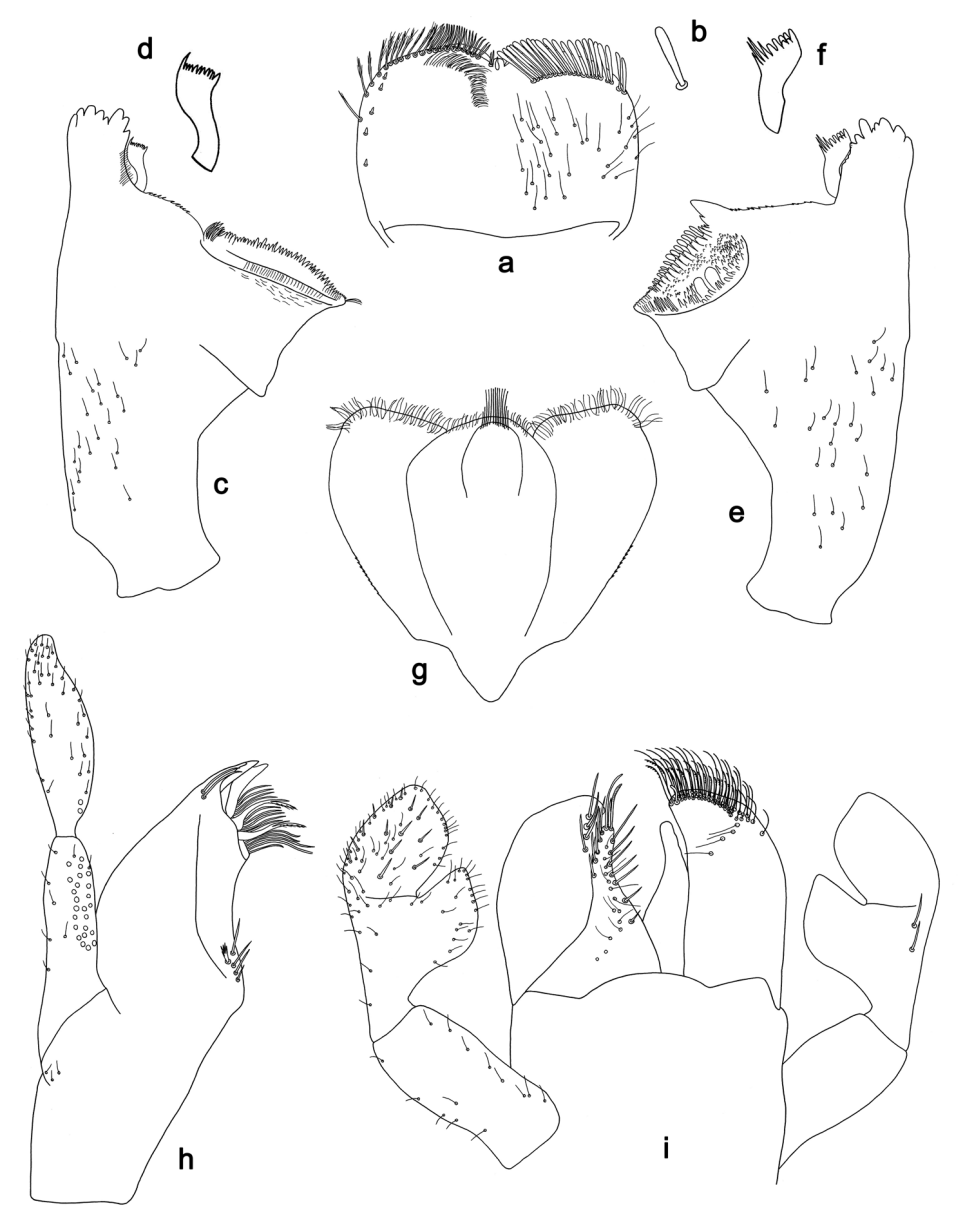
*Labiobaetis lubu* sp. nov., larva morphology: **a** Labrum **b** Seta of the submarginal arc on the dorsal surface of the labrum **c** Right mandible **d** Right prostheca **e** Left mandible **f** Left prostheca **g**Hypopharynx**h** Maxilla **i** Labium.

**Figure 19.**
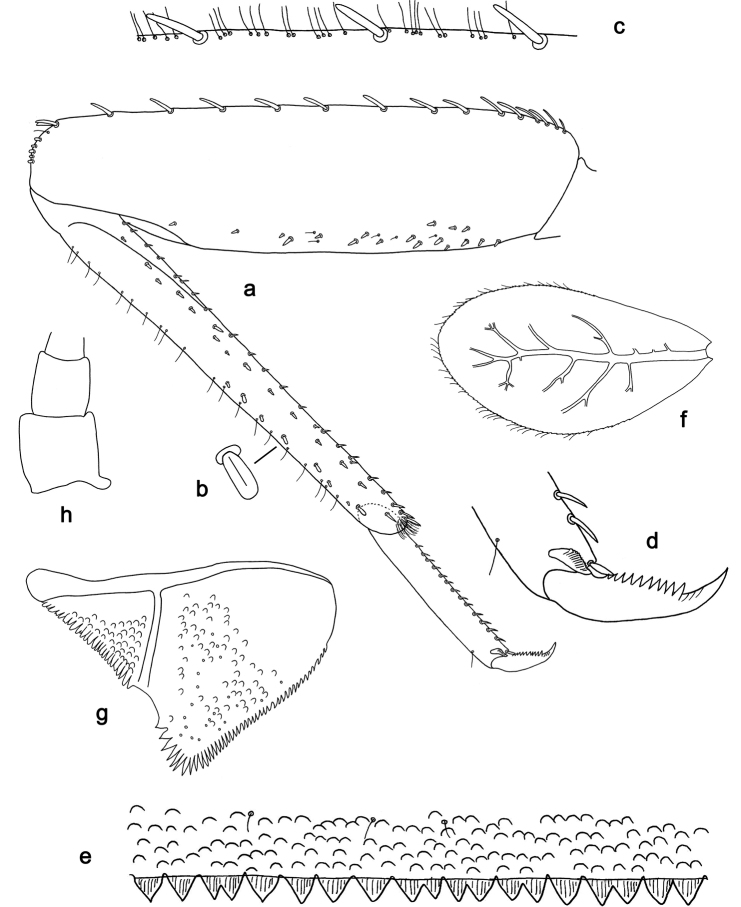
*Labiobaetis lubu* sp. nov., larva morphology: **a** Foreleg **b** Fore tibia dorsal seta **c** Fore femur dorsal margin **d** Fore claw **e** Tergum IV **f** Gill IV **g** Paraproct **h** Scape.

Figures 18 , 19 , 48c , 54b

##### Diagnosis.

**Larva.** Following combination of characters: A) dorsal surface of labrum with submarginal arc of 21–24 clavate setae; B) labial palp segment II with a large, lobed distomedial protuberance, segment III slightly pentagonal; C) left mandible without setae at apex of mola; D) fore femur rather slender, length 3.7× width, dorsal margin with a row of ca. 14 curved, spine-like setae and fine, simple setae; E) paraproct distally expanded, with ca. 45 stout marginal spines.

##### Description.

**Larva** (Figs 18, 19, 48c). Body length 7.2 mm.

*Colouration.* Head, thorax, and abdomen dorsally brown, head and thorax with bright median, dorsal suture, thorax with bright pattern as in Fig. 48c, forewing pads with bright striation. Head, thorax, and abdomen ventrally light brown, legs transparent with a brown, ventrodistomedial streak on femur, caudal filaments light brown.

*Antenna* with scape and pedicel subcylindrical, with poorly developed distolateral process at scape; flagellum with lanceolate spines and fine, simple setae on apex of each segment.

*Labrum* (Fig. 18a, b). Rectangular, length 0.7× maximum width. Distal margin with medial emargination and a small process. Dorsally with medium, fine, simple setae scattered over surface; submarginal arc of setae composed of 21–24 long, clavate setae. Ventrally with marginal row of setae composed of lateral and anterolateral long, feathered setae and medial long, bifid setae; ventral surface with five short, spine-like setae near lateral and anterolateral margin.

*Right mandible* (Fig. 18c, d). Incisors fused. Outer and inner sets of denticles with 4 + 3 denticles and one minute intermediate denticle. Inner margin of innermost denticle with a row of thin setae. Prostheca robust, apically denticulate. Margin between prostheca and mola slightly convex, with minute denticles. Tuft of setae at apex of mola present.

*Left mandible* (Fig. 18e, f). Incisors fused. Outer and inner sets of denticles with 4 + 3 denticles and one minute intermediate denticle. Prostheca robust, apically with small denticles and comb-shaped structure. Margin between prostheca and mola straight, with minute denticles towards subtriangular process. Subtriangular process long and slender, above level of area between prostheca and mola. Denticles of mola apically constricted. Tuft of setae at apex of mola absent.

Both mandibles with lateral margins almost straight. Basal half with fine, simple setae scattered over dorsal surface.

*Hypopharynx* (Fig. 18g). Lingua approx. as long as superlingua. Lingua longer than broad; medial tuft of stout setae well developed; distal half not expanded. Superlingua straight; lateral margin rounded; fine, long, simple setae along distal margin.

*Maxilla* (Fig. 18h). Galea-lacinia with two simple, robust apical setae under crown. Inner dorsal row of setae with three denti-setae, distal denti-seta tooth-like, middle and proximal denti-setae slender, bifid and pectinate. Medially with one bipectinate, spine-like seta and four long, simple setae. Maxillary palp 1.4× as long as length of galea-lacinia; two segmented; palp segment II 1.1× length of segment I; setae on maxillary palp fine, simple, scattered over surface of segments I and II; apex of last segment rounded, with excavation at inner distolateral margin.

*Labium* (Fig. 18i). Glossa basally broad, narrowing toward apex; shorter than paraglossa; inner margin with seven spine-like setae increasing in length distally; apex with two long and one medium, robust, pectinate setae; outer margin with five spine-like setae increasing in length distally; ventral surface with short, fine, simple, scattered setae. Paraglossa sub-rectangular, curved inward; apex rounded; with three rows of long, robust, distally pectinate setae in apical area and a row of four medium, simple setae in anteromedial area; dorsally with a row of four long, spine-like setae near inner margin. Labial palp with segment I 0.8× length of segments II and III combined. Segment I ventrally with short, fine, simple setae. Segment II with large, lobed distomedial protuberance; distomedial protuberance 0.8× width of base of segment III; inner and outer margin with short, fine, simple setae; dorsally with two long, spine-like, simple setae near outer margin. Segment III slightly pentagonal; apex slightly truncate; length 1.1× width; ventrally covered with short and medium spine-like, simple setae and short, fine, simple setae.

*Hind wing pads* absent.

*Foreleg* (Fig. 19a, b, c, d). Ratio of foreleg segments 1.2:1.0:0.4:0.1. *Femur*. Length ca. 4× maximum width. Dorsal margin with a row of ca. 14 curved, spine-like setae and fine, simple setae; length of setae 0.19× maximum width of femur. Apex rounded; with one pair of curved, spine-like setae and some short, stout setae. Many stout, lanceolate setae scattered along the ventral margin; femoral patch absent. *Tibia.* Dorsal margin with a row of stout, apically rounded setae and fine simple setae. Ventral margin with a row of curved, spine-like setae, on apex some spine-like, partly bipectinate setae and a tuft of fine, simple setae. Anterior surface scattered with stout, lanceolate setae. Patellotibial suture present on basal 1/3. *Tarsus.* Dorsal margin almost bare. Ventral margin with a row of curved, spine-like setae. Tarsal claw with one row of 10–12 denticles; distally pointed; with three stripes; subapical setae absent.

*Tergum* (Fig. 19e). Surface with rows of U-shaped scale bases and scattered fine, simple setae. Posterior margin of tergum IV with triangular spines, wider than long.

*Gills* (Fig. 19f). Present on segments II–VII. Margin with small denticles intercalating fine simple setae. Tracheae extending from main trunk to inner and outer margins. Gill IV as long as length of segments V and 1/3 VI combined. Gill VII as long as length of segment VIII.

*Paraproct* (Fig. 19g). Distally expanded, with > 40 stout marginal spines. Surface scattered with U-shaped scale bases and micropores. Cercotractor with numerous small marginal spines.

##### Etymology.

Dedicated to the indigenous Lubu people from Sumatra.

##### Distribution.

Indonesia: Sumatra.

##### Biological aspects.

The specimens were collected in a creek at an altitude of 1,050 m.

##### Type-material.

**Holotype.** Larva (on slide, GBIFCH 00592216), Indonesia, Sumatra Barat, Bukit Barisan, above Padang, creek, 1,047 m, 08.XI.2011, 00°56.74'S, 100°32.73'E, M. Balke leg. (UN3). Temporary deposited in MZL before definitely housed in MZB. **Paratypes.** 4 larvae (2 on slides, GBIFCH 00592217, GBIFCH 00592218, 2 in alcohol, GBIFCH 00515342, deposited in MZL), same data as holotype.

#### 12. Labiobaetis pakpak sp. nov.

Taxon classificationAnimaliaEphemeropteraBaetidae

39CDDCC1-03A6-50E9-ABDA-F1D5E92FFFAF

http://zoobank.org/5EAF4EA0-6625-48E2-9EC9-079ED6C55504

**Figure 20.**
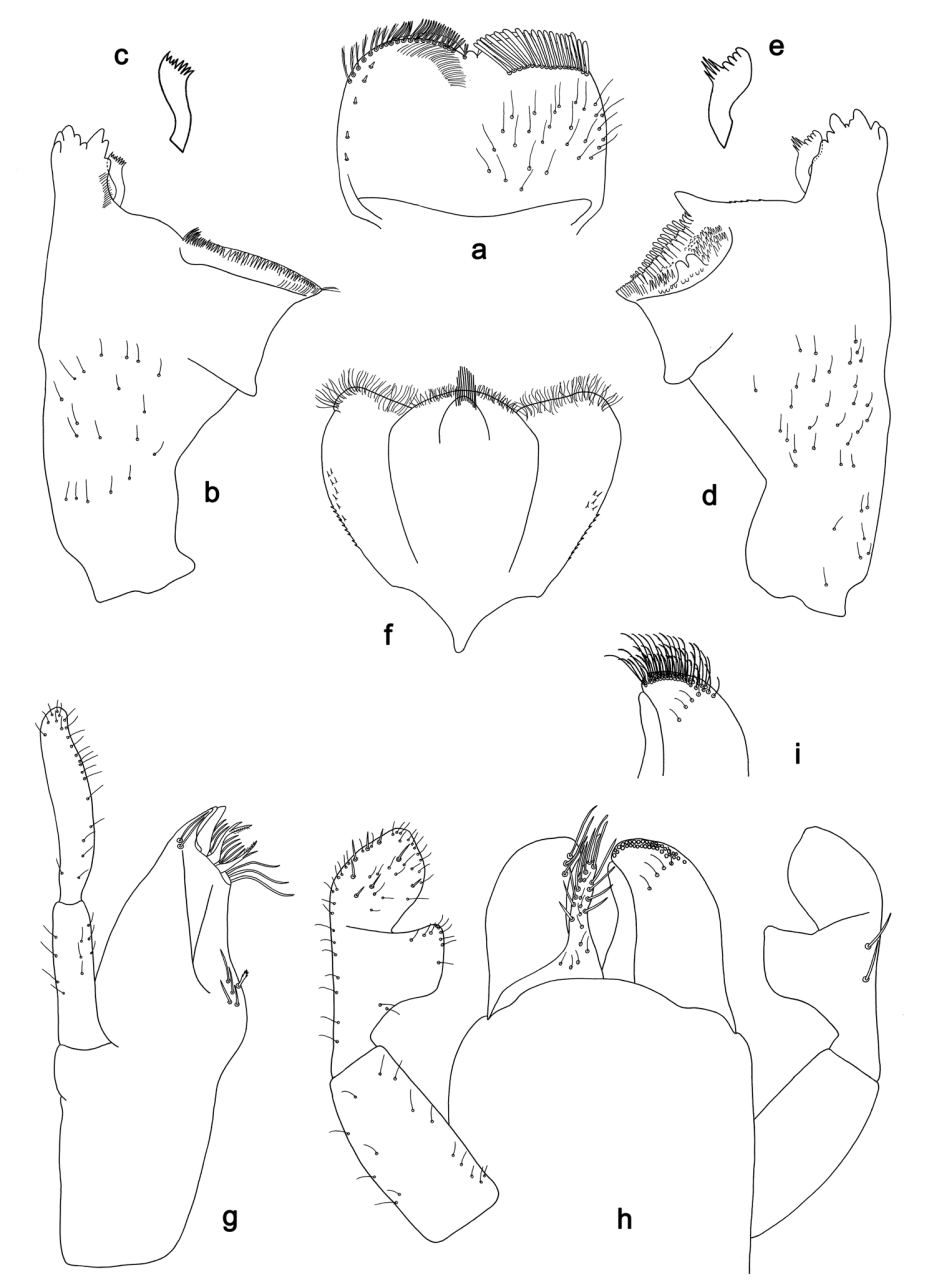
*Labiobaetis pakpak* sp. nov., larva morphology: **a** Labrum **b** Right mandible **c** Right prostheca **d** Left mandible **e** Left prostheca **f**Hypopharynx**g** Maxilla **h** Labium **i** Apex of paraglossa.

**Figure 21.**
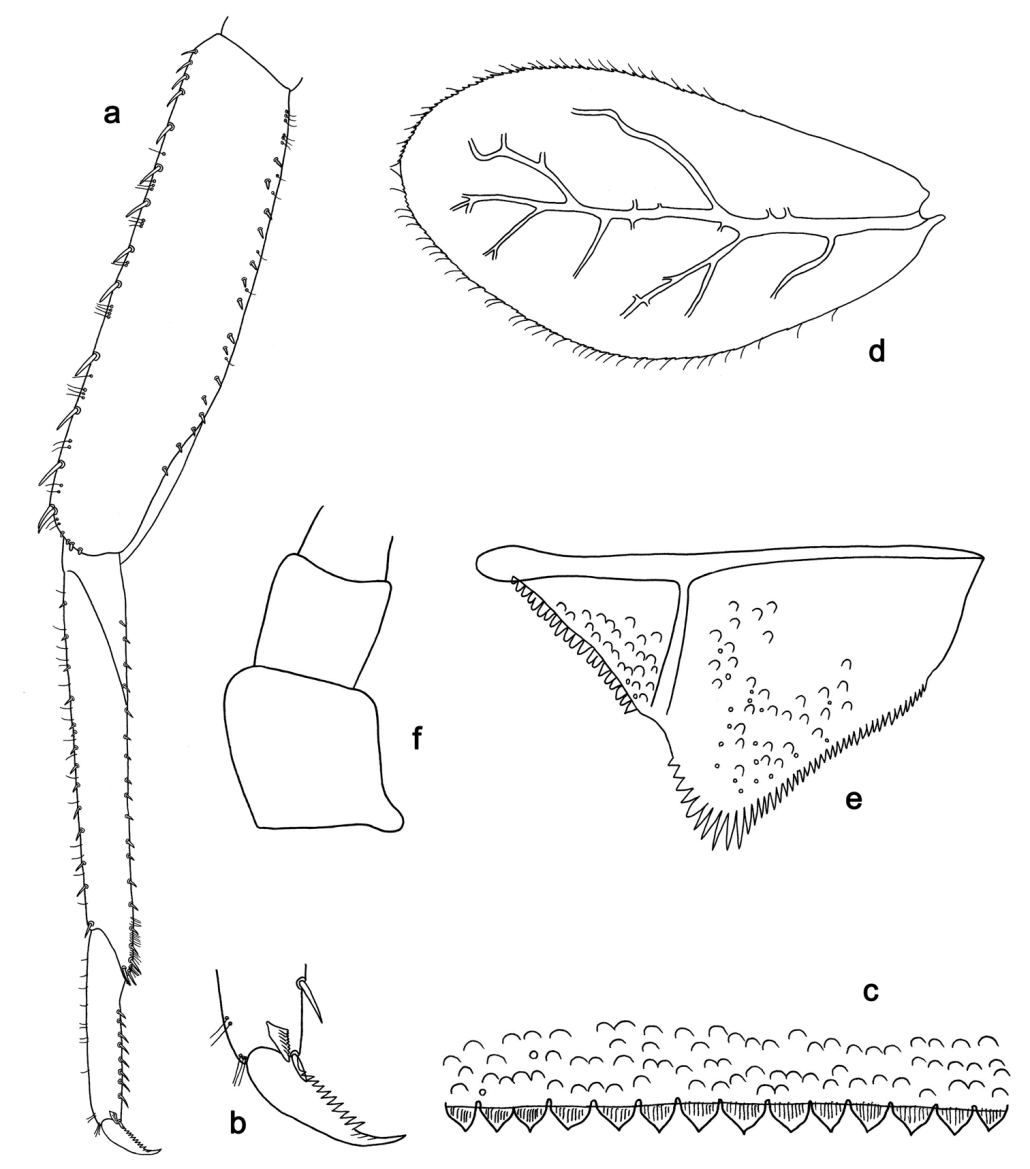
*Labiobaetis pakpak* sp. nov., larva morphology: **a** Foreleg **b** Fore claw **c** Tergum IV **d** Gill IV **e** Paraproct **f** Scape.

Figures 20 , 21 , 48d , 55a

##### Diagnosis.

**Larva.** Following combination of characters: A) dorsal surface of labrum with submarginal arc of 18–19 clavate setae; B) labial palp segment II with large, lobed distomedial protuberance, segment III slightly pentagonal; C) left mandible without setae at apex of mola; D) fore femur rather slender, length ca. 4× maximum width, dorsal margin with a row of 12–15 spine-like setae and many fine, simple setae; E) paraproct distally expanded, with > 40 stout marginal spines.

##### Description.

**Larva** (Figs 20, 21, 48d). Body length 3.7 mm.

*Colouration*. Head, thorax, and abdomen dorsally brown, head and thorax with bright median, dorsal suture. Head, thorax, and abdomen ventrally light brown, legs transparent, caudal filaments light brown.

*Antenna* with scape and pedicel subcylindrical, without distolateral process at scape; flagellum with lanceolate spines and fine, simple setae on apex of each segment.

*Labrum* (Fig. 20a). Rectangular, length 0.7× maximum width. Distal margin with medial emargination and a small process. Dorsally with long, fine, simple setae scattered over surface; submarginal arc of setae composed of 18–19 long, clavate setae. Ventrally with marginal row of setae composed of lateral and anterolateral long, feathered setae and medial long, bifid setae; ventral surface with five short, spine-like setae near lateral and anterolateral margin.

*Right mandible* (Fig. 20b, c). Incisors fused. Outer and inner sets of denticles with 4 + 3 denticles and one minute intermediate denticle. Inner margin of innermost denticle with a row of thin setae. Prostheca robust, apically denticulate. Margin between prostheca and mola slightly convex. Tuft of setae at apex of mola present.

*Left mandible* (Fig. 20d, e). Incisors fused. Outer and inner sets of denticles with 3 + 3 denticles and one minute intermediate denticle. Prostheca robust, apically with small denticles and comb-shaped structure. Margin between prostheca and mola straight, with minute denticles towards subtriangular process. Subtriangular process long and slender, above level of area between prostheca and mola. Denticles of mola apically constricted. Tuft of setae at apex of mola absent.

Both mandibles with lateral margins almost straight. Basal half with fine, simple setae scattered over dorsal surface.

*Hypopharynx* (Fig. 20f). Lingua approx. as long as superlingua. Lingua longer than broad; medial tuft of stout setae well developed; distal half laterally expanded. Superlingua straight; lateral margin rounded; fine, long, simple setae along distal margin.

*Maxilla* (Fig. 20g). Galea-lacinia with two simple, robust apical setae under crown. Inner dorsal row of setae with three denti-setae, distal denti-seta tooth-like, middle and proximal denti-setae slender, bifid and pectinate. Medially with one bipectinate, spine-like seta and 3–4 medium, simple setae. Maxillary palp 1.4× as long as length of galea-lacinia; two segmented; palp segment II 1.3× length of segment I; setae on maxillary palp fine, simple, scattered over surface of segments I and II; apex of last segment rounded, with slight excavation at inner distolateral margin.

*Labium* (Fig. 20h, i). Glossa basally broad, narrowing toward apex; shorter than paraglossa; inner margin with 4–6 spine-like setae increasing in length distally; apex with two long and one medium, robust setae; outer margin with 4–6 long, spine-like setae increasing in length distally; ventral surface with short, fine, simple, scattered setae. Paraglossa sub-rectangular, curved inward; apex rounded; with three rows of long, robust, distally pectinate setae in apical area and a row of three or four medium, simple setae in anteromedial area; dorsally with a row of three long, spine-like setae near inner margin. Labial palp with segment I 0.8× length of segments II and III combined. Segment I ventrally with short, fine, simple setae. Segment II with large, lobed distomedial protuberance; distomedial protuberance 0.8× width of base of segment III; inner and outer margin with short, fine, simple setae; dorsally with two long, spine-like, simple setae near outer margin. Segment III slightly pentagonal; apex slightly truncate; length 1.2× width; ventrally covered with short, spine-like, simple setae and short, fine, simple setae.

*Hind wing pads* absent.

*Foreleg* (Fig. 21a, b). Ratio of foreleg segments 1.3:1.0:0.5:0.2. *Femur*. Length ca. 4× maximum width. Dorsal margin with a row 12–15 spine-like setae and many fine, simple setae; length of setae 0.23× maximum width of femur. Apex rounded; with 1–2 pairs of curved, spine-like setae and some short, stout setae. Many stout, lanceolate setae scattered along the ventral margin; femoral patch poorly developed. *Tibia*. Dorsal margin with a row of short, stout setae (not always present) and fine, simple setae. Ventral margin with a row of short, spine-like setae, on apex some stout, spine-like, partially bipectinate setae and a tuft of fine, simple setae. Patellotibial suture present on basal 1/3. *Tarsus*. Dorsal margin with a row of fine, simple setae. Ventral margin with a row of curved, spine-like setae. Tarsal claw with one row of 10–11 denticles; distally pointed; with three stripes; subapical setae absent.

*Tergum* (Fig. 21c). Surface with rows of U-shaped scale bases and scattered micropores. Posterior margin of tergum IV with triangular spines, wider than long.

*Gills* (Fig. 21d). Present on segments II–VII. Margin with small denticles intercalating fine simple setae. Tracheae extending from main trunk to inner and outer margins. Gill IV as long as length of segments V and VI combined. Gill VII as long as length of segments VIII and 1/2 IX combined.

*Paraproct* (Fig. 21e). Distally expanded, with > 40 stout marginal spines. Surface scattered with U-shaped scale bases and micropores. Cercotractor with numerous small marginal spines.

##### Etymology.

Dedicated to the indigenous Pakpak people from Sumatra.

##### Distribution.

Indonesia: Sumatra.

##### Biological aspects.

The specimens were collected in forest at an altitude of 490 m.

##### Type-material.

**Holotype.** Larva (on slide, GBIFCH 00592219), Indonesia, Sumatra Barat, Sijunjung / Muara area, forest, 488 m, 10.XI.2011, 00°40.10'S, 101°07.26'E, M. Balke leg. (UN7). Temporary deposited in MZL before definitely housed in MZB. **Paratypes.** 10 larvae (2 on slides, GBIFCH 00592221, GBIFCH 00592220, 3 in alcohol, GBIFCH 00235851, GBIFCH 00235781, GBIFCH 00515344, deposited in MZL; 5 in alcohol, GBIFCH 00515345, deposited in ZSM), same data as holotype.

#### 13. Labiobaetis paradiffundus sp. nov.

Taxon classificationAnimaliaEphemeropteraBaetidae

D5B148EA-78FA-5AAA-B53A-128E92D87BA4

http://zoobank.org/1B6B4283-55F6-40FA-8D8C-7C6042409677

**Figure 22.**
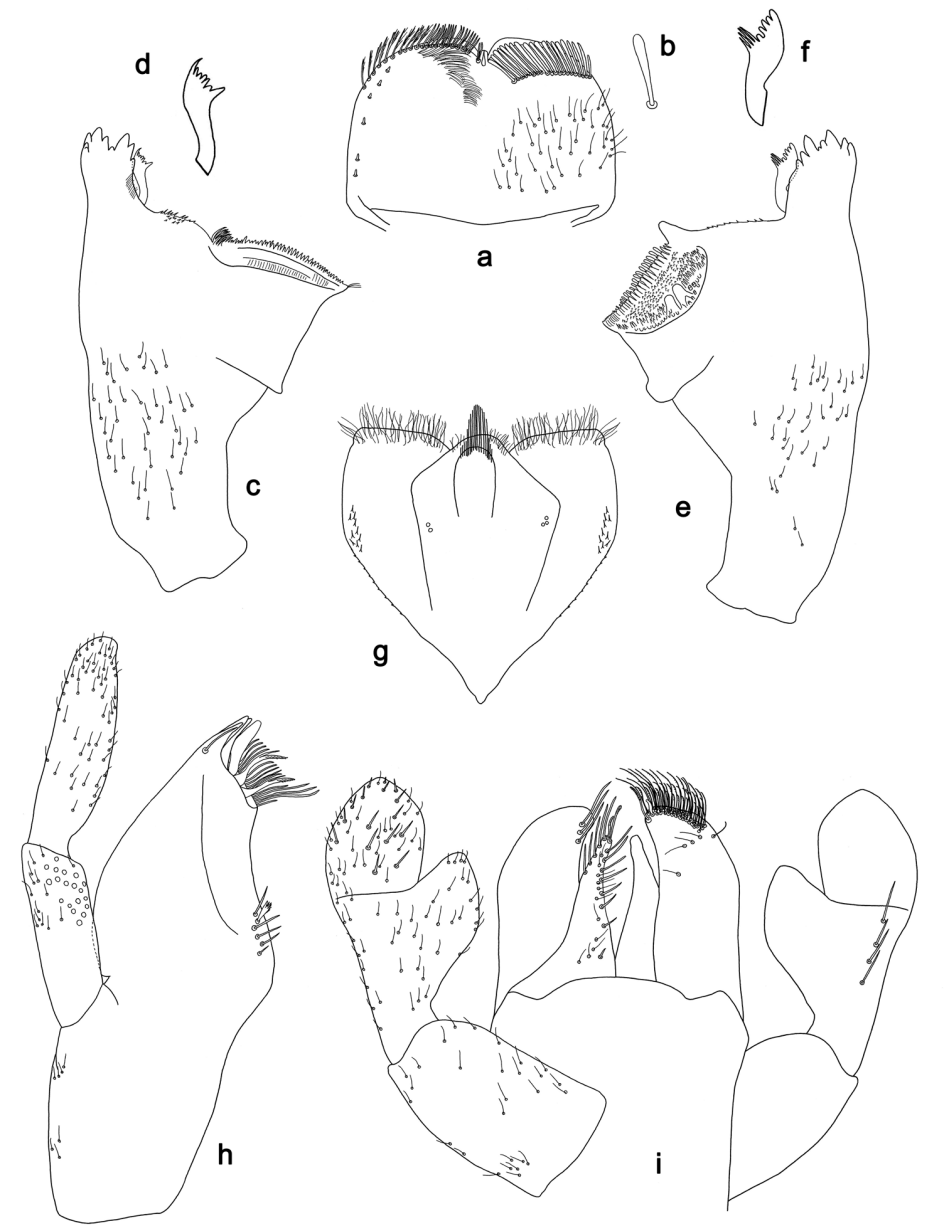
*Labiobaetis paradiffundus* sp. nov., larva morphology: **a** Labrum **b** Seta of the submarginal arc on the dorsal surface of the labrum **c** Right mandible **d** Right prostheca **e** Left mandible **f** Left prostheca **g**Hypopharynx**h** Maxilla **i** Labium.

**Figure 23.**
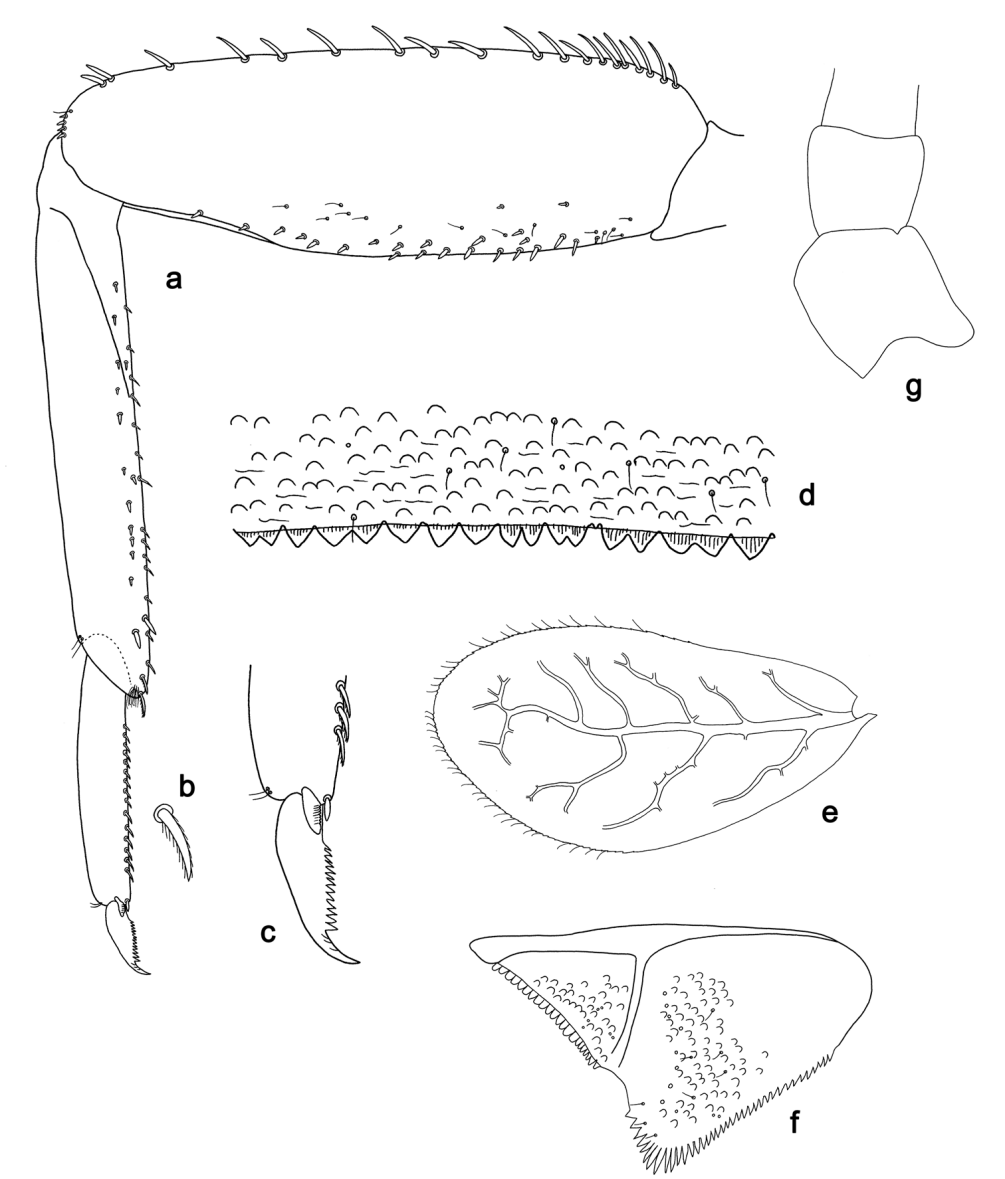
*Labiobaetis paradiffundus* sp. nov., larva morphology: **a** Foreleg **b** Fore tarsus ventral seta **c** Fore claw **d** Tergum IV **e** Gill IV **f** Paraproct **g** Scape.

Figures 22 , 23 , 49a , 54b

##### Diagnosis.

**Larva.** Following combination of characters: A) dorsal surface of labrum with submarginal arc of 20–24 clavate setae; B) labial palp segment II with a large, thumb-like distomedial protuberance, segment III slightly pentagonal; C) left mandible without setae at apex of mola; D) foreleg femur rather broad, length ca. 3× maximum width, dorsal margin with a row of ca. 16 curved, spine-like setae; E) paraproct distally expanded, with 37–48 stout marginal spines.

##### Description.

**Larva** (Figs 22, 23, 49a). Body length 6.0 mm.

*Colouration*. Head, thorax, and abdomen dorsally brown, head and thorax with bright median, dorsal suture, thorax with bright pattern as in Fig. 49a, abdominal segments IX and X light brown, forewing pads with dark brown striation and bright striation near margin. Head, thorax, and abdomen ventrally light brown, legs transparent, ventral margin of femur brown, caudal filaments light brown.

*Antenna* with scape and pedicel subcylindrical, with poorly developed distolateral process at scape; flagellum with lanceolate spines and fine, simple setae on apex of each segment.

*Labrum* (Fig. 22a, b). Rectangular, length 0.7× maximum width. Distal margin with medial emargination and a small process. Dorsally with medium, fine, simple setae scattered over surface; submarginal arc of setae composed of 20–24 long, clavate setae. Ventrally with marginal row of setae composed of anterolateral long, feathered setae and medial long, bifid setae; ventral surface with six short, spine-like setae near lateral and anterolateral margin.

*Right mandible* (Fig. 22c, d). Incisors fused. Outer and inner sets of denticles with 4 + 3 denticles and one minute intermediate denticle. Inner margin of innermost denticle with a row of thin setae. Prostheca robust, apically denticulate. Margin between prostheca and mola slightly convex, with minute denticles. Tuft of setae at apex of mola present.

*Left mandible* (Fig. 22e, f). Incisors fused. Outer and inner sets of denticles with 4 + 3 denticles and one minute intermediate denticle. Prostheca robust, apically with small denticles and comb-shaped structure. Margin between prostheca and mola straight, with minute denticles towards subtriangular process. Subtriangular process long and slender, above level of area between prostheca and mola. Denticles of mola apically constricted. Tuft of setae at apex of mola absent.

Both mandibles with lateral margins almost straight. Basal half with fine, simple setae scattered over dorsal surface.

*Hypopharynx* (Fig. 22g). Lingua shorter than superlingua. Lingua longer than broad; medial tuft of stout setae well developed; distal half laterally expanded. Superlingua straight; lateral margin rounded; fine, long, simple setae along distal margin.

*Maxilla* (Fig. 22h). Galea-lacinia with one simple, robust apical seta under crown. Inner dorsal row of setae with three denti-setae, distal denti-seta tooth-like, middle and proximal denti-setae slender, bifid and pectinate. Medially with one bipectinate, spine-like seta and five long, simple setae. Maxillary palp 1.3× as long as length of galea-lacinia; two segmented; palp segment II 1.4× length of segment I; setae on maxillary palp fine, simple, scattered over surface of segments I and II; apex of last segment rounded, with slight excavation at inner distolateral margin.

*Labium* (Fig. 22i). Glossa basally broad, narrowing toward apex; shorter than paraglossa; inner margin with eight spine-like setae increasing in length distally; apex with two long and one medium, robust, pectinate setae; outer margin with 4–5 long, spine-like setae increasing in length distally; ventral surface with short, fine, simple setae. Paraglossa sub-rectangular, curved inward; apex rounded; with three rows of long, robust, distally pectinate setae in apical area and four medium, simple setae in anteromedial area; dorsally with a row of three long, spine-like setae near inner margin. Labial palp with segment I 0.8× length of segments II and III combined. Segment I ventrally with short, fine, simple setae. Segment II with broad thumb-like distomedial protuberance; distomedial protuberance 0.7× width of base of segment III; inner and outer margin with short, fine, simple setae; dorsally with a row of 3–4 long, spine-like, simple setae near outer margin. Segment III slightly pentagonal; apex slightly pointed; length 1.2× width; ventrally covered with short and medium spine-like, simple setae and short, fine, simple setae.

*Hind wing pads* absent.

*Foreleg* (Fig. 23a, b, c). Ratio of foreleg segments 1.2:1.0:0.5:0.2. *Femur*. Length ca. 3× maximum width. Dorsal margin with a row of ca. 16 curved, spine-like setae; length of setae 0.21× maximum width of femur. Apex rounded; with one pair of curved, spine-like setae and some short, stout setae. Many stout, lanceolate setae scattered along the ventral margin; femoral patch poorly developed. *Tibia*. Dorsal margin bare. Ventral margin with a row of curved, spine-like setae, on apex two bipectinate, spine-like setae and a tuft of fine, simple setae. Anterior surface scattered with stout, lanceolate setae. Patellotibial suture present on basal 1/2. *Tarsus.* Dorsal margin bare. Ventral margin with a row of curved, spine-like, bipectinate setae. Tarsal claw with one row of 10–13 denticles; distally pointed; with three stripes; subapical setae absent.

*Tergum* (Fig. 23d). Surface with irregular rows of U-shaped scale bases and scattered fine, simple setae and micropores. Posterior margin of tergum IV with triangular spines, wider than long.

*Gills* (Fig. 23e). Present on segments II–VII. Margin with small denticles intercalating fine simple setae. Tracheae extending from main trunk to inner and outer margins. Gill IV as long as length of segments V and 2/3 VI combined. Gill VII as long as length of segments VIII and 1/3 IX combined.

*Paraproct* (Fig. 23f). Distally expanded, with 37–48 stout marginal spines. Surface scattered with U-shaped scale bases, fine, simple setae and micropores. Cercotractor with numerous small marginal spines.

##### Etymology.

Refers to the morphological similarity with *L. diffundus*.

##### Distribution.

Indonesia: Sumatra.

**Biological aspects.** The specimens were collected at altitudes from 890 m to 1,830 m in small to medium (0.5 m – 5 m wide), shallow (5 cm – 10 cm deep) and slow to medium fast (velocity 0.2 m/s – 0.7 m/s) forest streams or, in one case, a highly disturbed stream (agriculture, waste). The substrate was predominantly bedrock, boulder, stones, gravel and sand and only rarely with leaf litter or dead wood.

##### Type-material.

**Holotype.** Larva (on slide, GBIFCH 00592213), Indonesia, Sumatra, volcano Marapi, 00°23.65'S, 100°25.48E, 1,403 m, 04.IV.2014, M. Gueuning leg. Temporary deposited in MZL before definitely housed in MZB. **Paratypes.** 33 larvae (3 on slides, GBIFCH 00592214, GBIFCH 00592215, GBIFCH 00422188, 23 in alcohol, GBIFCH 00422059, GBIFCH 00422191, GBIFCH 00422054, GBIFCH 00422144, GBIFCH 00422095, GBIFCH 00422179, GBIFCH 00422212, GBIFCH 00422214, GBIFCH 00422658, GBIFCH 00422660, GBIFCH 00422164, GBIFCH 00422180, GBIFCH 00422096, deposited in MZL; 6 larvae in alcohol, GBIFCH 00515341, GBIFCH 00422139, GBIFCH 00422155, deposited in ZSM), same data as holotype.

##### Other material.

6 larvae (in alcohol, GBIFCH 00422131, GBIFCH 00422193, GBIFCH 00422146, GBIFCH 00422199, GBIFCH 00422161, GBIFCH 00422615), Indonesia, Sumatra, volcano Sago, Simbukan River, 00°18.93'S, 100°40.73'E, 1,645 m, 17.III.2014, M. Gueuning leg.; 2 larvae (in alcohol, GBIFCH 00422138, GBIFCH 00422171), Indonesia, Sumatra, volcano Sago, River Kaligain, 00°18.02'S, 100°40.13'E, 1,040 m, 05.IV.2014, M. Gueuning leg.; 3 larvae (in alcohol, GBIFCH 00422143, GBIFCH 00422150, GBIFCH 00422210), Indonesia, Sumatra, volcano Singgalan, River Pagu Pagu, 00°24.20'S, 100°22.72'E, 1,185 m, 22.III.2014, M. Gueuning leg.; 8 larvae (in alcohol, GBIFCH 00422169, GBIFCH 00422151, GBIFCH 00422127, GBIFCH 00422078, GBIFCH00422204, GBIFCH 00422136, GBIFCH 00422931), Indonesia, Sumatra, volcano Singgalan, River Pagu Pagu, 00°23.53'S, 100°21.45'E, 1,785 m, 22.III.2014, M. Gueuning leg.; 3 larvae (in alcohol, GBIFCH 00422091, GBIFCH 00422102, GBIFCH 00422187), Indonesia, Sumatra, volcano Singgalang, River Caruak, 00°22.93'S, 100°22.70'E, 1,300 m, 23.III.2014, M. Gueuning leg.; 1 larva (in alcohol, GBIFCH 00422189), Indonesia, Sumatra, volcano Singgalang, River Caruak, 00°23.05'S, 100°21.40'E, 1,640 m, 23.III.2014, M. Gueuning leg.; 3 larvae (in alcohol, GBIFCH 00422192, GBIFCH 00422140, GBIFCH 00422213), Indonesia, Sumatra, volcano Singgalang, River Sianok, 00°19.95'S, 100°19.32'E, 1,150 m, 24.III.2014, M. Gueuning leg.; 5 larvae (in alcohol, GBIFCH 00422196, GBIFCH 00422055, GBIFCH 00422145, GBIFCH 00422223, GBIFCH 00422172), Indonesia, Sumatra, volcano Singgalan, River Sianok, 1,350 m, 24.III.2014, M. Gueuning leg.; 7 larvae (in alcohol, GBIFCH 00422174, GBIFCH 00422202, GBIFCH 00422148, GBIFCH 00422207, GBIFCH 00422178, GBIFCH 00422137, GBIFCH 00422661), Indonesia, Sumatra, volcano Talamau, River Karumiang, 00°05.35'N, 99°58.10'E, 1,830 m, 29.III.2014, M. Gueuning leg. All material deposited in MZL.

#### 14. Labiobaetis rimba sp. nov.

Taxon classificationAnimaliaEphemeropteraBaetidae

1142ACA3-3FA0-5820-BE2B-D2999AFE2698

http://zoobank.org/F49E0FC5-75FD-4518-9E94-FA9672E07F39

**Figure 24.**
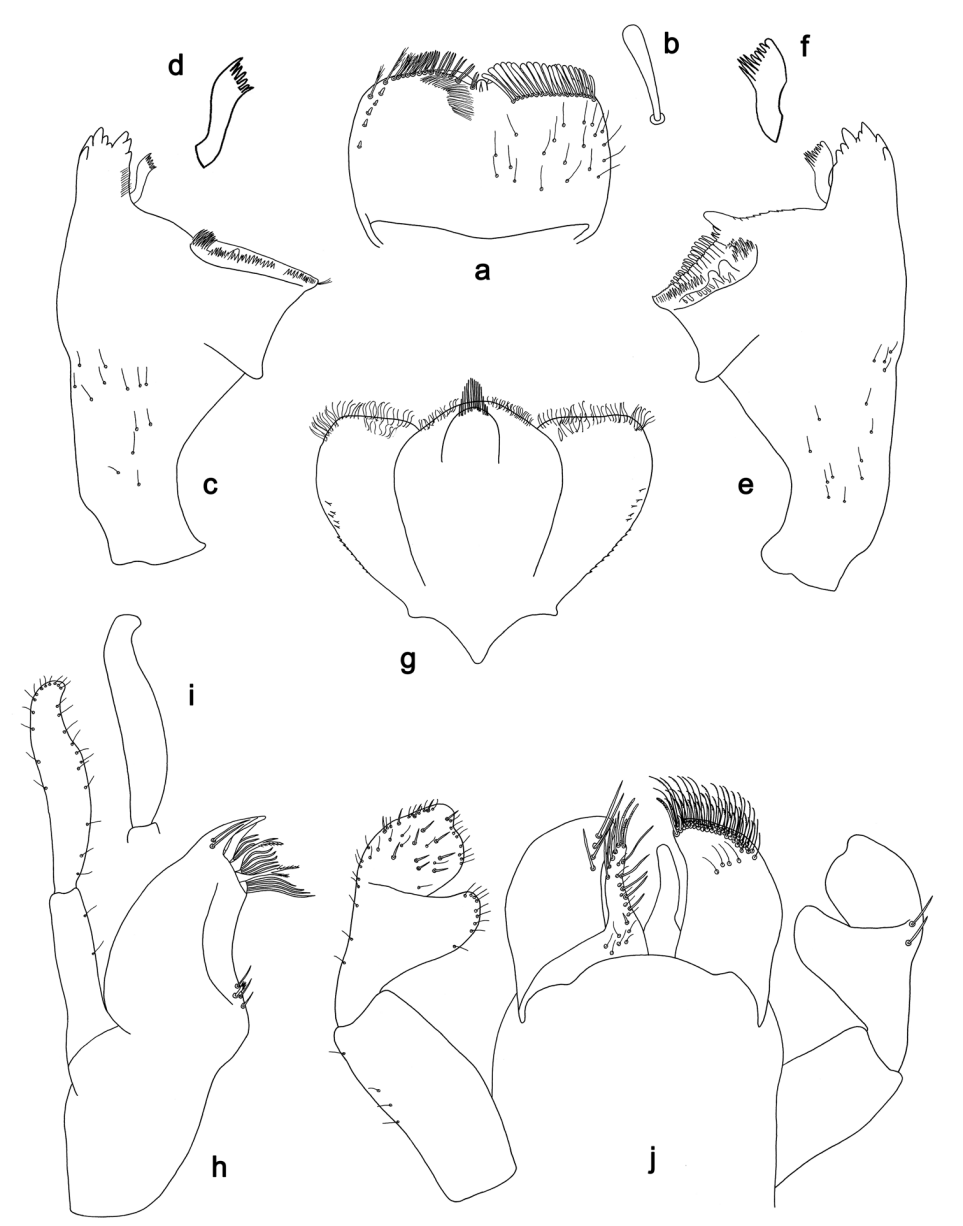
*Labiobaetis rimba* sp. nov., larva morphology: **a** Labrum **b** Seta of the submarginal arc on the dorsal surface of the labrum **c** Right mandible **d** Right prostheca **e** Left mandible **f** Left prostheca **g**Hypopharynx**h** Maxilla **i** Maxillary palp segment II **h** Labium.

**Figure 25.**
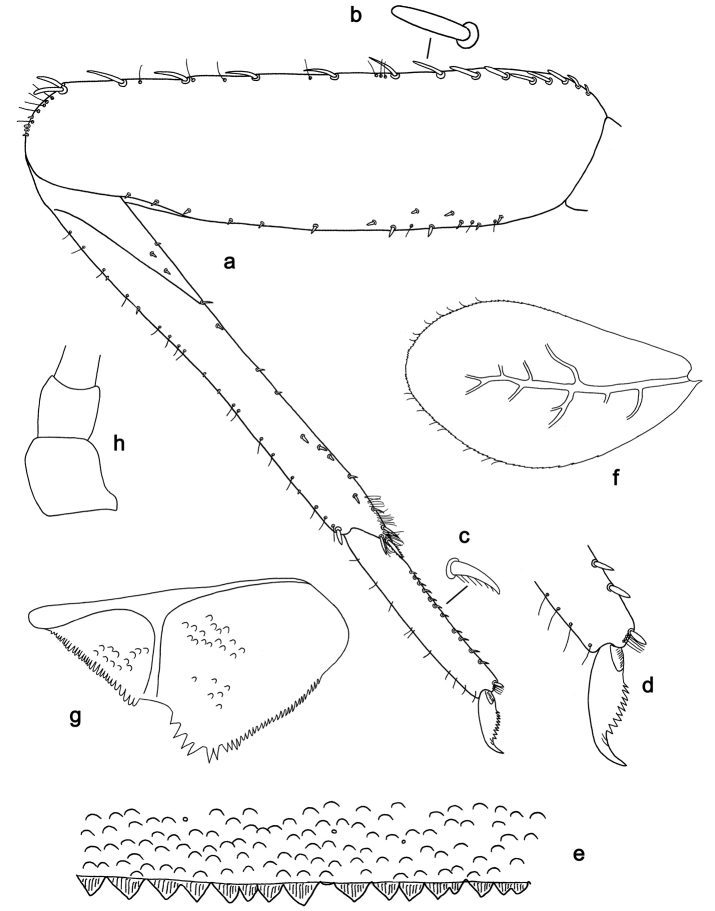
*Labiobaetis rimba* sp. nov., larva morphology: **a** Foreleg **b** Fore femur dorsal seta **c** Fore tarsus ventral seta **d** Fore claw **e** Tergum IV **f** Gill IV **g** Paraproct **h** Scape.

Figures 24, 25, 49b, 55a

##### Diagnosis.

**Larva.** Following combination of characters: A) dorsal surface of labrum with submarginal arc of 20 clavate setae; B) labial palp segment II with an elongated, thumb-like distomedial protuberance, segment III slightly pentagonal, apically with a small projection; C) left mandible without setae at apex of mola; D) maxillary palp much longer than galea-lacinia, apically rounded and curved inward, with excavation at inner distolateral margin; E) fore femur rather slender, length ca. 4× maximum width, dorsal margin with a row of 10–13 curved, spine-like setae; F) paraproct distally not expanded, with ca. 32 stout marginal spines.

##### Description.

**Larva** (Figs 24, 25, 49b). Body length 4.2 mm.

*Colouration*. Head and thorax dorsally brown, with bright median, dorsal suture, thorax with pattern as in Fig. 49b, forewing pads with dark striation. Abdomen dorsally reddish-brown. Head, thorax, and abdomen ventrally light brown, legs transparent with brown spots distomedially on femur, caudal filaments light brown.

*Antenna* with scape and pedicel subcylindrical, without distolateral process at scape; flagellum with broad, lanceolate spines and fine, simple setae on apex of each segment.

*Labrum* (Fig. 24a, b). Rectangular, length 0.6× maximum width. Distal margin with medial emargination and a small process. Dorsally with medium, fine, simple setae scattered over surface; submarginal arc of setae composed of 20 long, clavate setae. Ventrally with marginal row of setae composed of anterolateral long, feathered setae and medial long, bifid setae; ventral surface with five short, spine-like setae near lateral and anterolateral margin.

*Right mandible* (Fig. 24c, d). Incisors fused. Outer and inner sets of denticles with 4 + 3 denticles and one minute intermediate denticle. Inner margin of innermost denticle with a row of thin setae. Prostheca robust, apically denticulate. Margin between prostheca and mola straight. Tuft of setae at apex of mola present.

*Left mandible* (Fig. 24e, f). Incisors fused. Outer and inner sets of denticles with 4 + 3 denticles and one minute intermediate denticle. Prostheca robust, apically with small denticles and comb-shaped structure. Margin between prostheca and mola straight, with minute denticles towards subtriangular process. Subtriangular process long and slender, above level of area between prostheca and mola. Denticles of mola apically constricted. Tuft of setae at apex of mola absent.

Both mandibles with lateral margins almost straight. Basal half with fine, simple setae scattered over dorsal surface.

*Hypopharynx* (Fig. 24g). Lingua longer than superlingua. Lingua longer than broad; medial tuft of stout setae well developed; distal half laterally expanded. Superlingua straight; lateral margin rounded; fine, long, simple setae along distal margin.

*Maxilla* (Fig. 24h, i). Galea-lacinia with two simple, robust apical setae under crown. Inner dorsal row of setae with three denti-setae, distal denti-seta tooth-like, middle and proximal denti-setae slender, bifid and pectinate. Medially with one bipectinate, spine-like seta and 3–4 medium, simple setae. Maxillary palp 1.5× as long as length of galea-lacinia; two segmented; palp segment II 1.4× length of segment I; setae on maxillary palp fine, simple, scattered over surface of segments I and II; apex of last segment rounded and curved inward, with excavation at inner distolateral margin.

*Labium* (Fig. 24j). Glossa basally broad, narrowing toward apex; shorter than paraglossa; inner margin with six spine-like setae increasing in length distally; apex with two long and one medium, robust, pectinate setae; outer margin with 4 long, spine-like setae; ventral surface with short, fine, simple setae. Paraglossa sub-rectangular, curved inward; apex rounded; with three rows of long, robust, distally pectinate setae in apical area and 4–5 medium, simple setae in anteromedial area; dorsally with a row of four long, spine-like setae near inner margin. Labial palp with segment I approx. as long as length of segments II and III combined. Segment I ventrally with short, fine, simple setae. Segment II with elongated, thumb-like distomedial protuberance; distomedial protuberance 0.8× width of base of segment III; inner and outer margin with short, fine, simple setae; dorsally with two long, spine-like, simple setae near outer margin. Segment III slightly pentagonal; apex with small projection; length 1.0× width; ventrally covered with short, spine-like, simple setae and short, fine, simple setae.

*Foreleg* (Fig. 25a, b, c, d). Ratio of foreleg segments 1.2:1.0:0.4:0.1. *Femur*. Length ca. 4× maximum width. Dorsal margin with a row of 10–13 curved, spine-like, apically rounded setae; length of setae 0.22× maximum width of femur. Apex rounded; with one pair of curved, spine-like setae, some short stout setae and some fine, simple setae. Short to medium stout, lanceolate setae scattered along the ventral margin; femoral patch reduced to a few setae. *Tibia*. Dorsal margin with a row of minute, stout setae and fine, simple setae. Ventral margin with a row of curved, spine-like setae, on apex some stout, spine-like and partly bipectinate setae and a tuft of fine, simple setae. Anterior surface scattered with stout, lanceolate setae. Patellotibial suture present on basal 1/3. *Tarsus*. Dorsal margin with a row of fine, simple setae. Ventral margin with a row of curved, spine-like, bipectinate setae (pectination difficult to see). Tarsal claw with one row of 9–10 denticles; distally pointed; with two stripes; subapical setae absent.

*Tergum* (Fig. 25e). Surface with rows of U-shaped scale bases and scattered micropores. Posterior margin of tergum IV with triangular spines, wider than long.

*Gills* (Fig. 25f). Present on segments II–VII. Margin with small denticles intercalating fine simple setae. Tracheae extending from main trunk to inner and outer margins. Gill IV as long as length of segments V and 1/2 VI combined. Gill VII as long as length of segments VIII and 1/4 IX combined.

*Paraproct* (Fig. 25g). Distally not expanded, with ca. 32 stout marginal spines. Surface scattered with U-shaped scale bases. Cercotractor with numerous small marginal spines.

##### Etymology.

Dedicated to the indigenous Rimba people from Sumatra.

##### Distribution.

Indonesia: Sumatra.

##### Biological aspects.

The specimens were collected at altitudes of 75 m and 275 m.

##### Type-material.

**Holotype.** Larva (on slide, GBIFCH 00592222), Indonesia, Sumatra Barat, Barung-Barung, 01°06.03'S, 100°29.12'E, 75 m, 24.V.2010, J.-M. Elouard leg. Temporary deposited in MZL before definitely housed in MZB. **Paratypes.** 2 larvae (1 on slide, GBIFCH 00592223, 1 in alcohol, GBIFCH 00515366, deposited in MZL), same data as holotype; 1 larva (on slide, GBIFCH 00592224, deposited in MZL), Indonesia, Sumatra Barat, Sawahlunto, stream, 275 m, 10.XI.2011, 00°41.33'S, 100°46.72'E, M. Balke leg. (UN5).

### *Labiobaetis gueuningi* group of species

With the following combination of characters: A) dorsal surface with submarginal arc of simple setae; B) labial palp segment II with thumb-like distomedial protuberance; C) maxillary palp with three segments; D) seven pairs of gills; E) spines at posterior margin of abdominal terga with medial enhancement; F) hindwing pads absent; G) distolateral process at scape well developed.

#### 15. Labiobaetis gueuningi sp. nov.

Taxon classificationAnimaliaEphemeropteraBaetidae

437F57CC-1716-5E09-AB20-6A38A6C60DCB

http://zoobank.org/DEAD4F24-1884-4EDB-9CF6-2D0D78C94BE3

**Figure 26.**
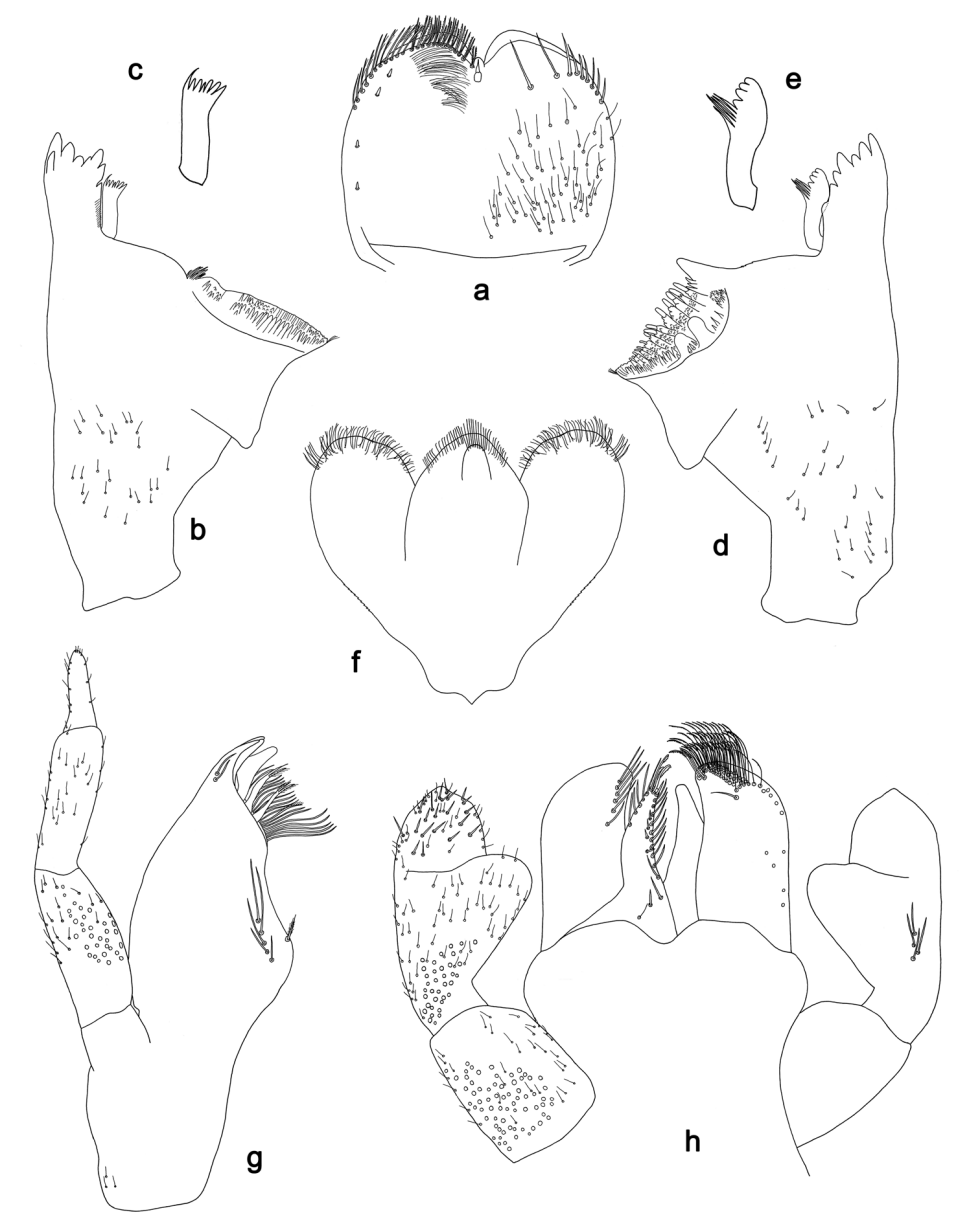
*Labiobaetis gueuningi* sp. nov., larva morphology: **a** Labrum **b** Right mandible **c** Right prostheca **d** Left mandible **e** Left prostheca **f**Hypopharynx**g** Maxilla **h** Labium.

**Figure 27.**
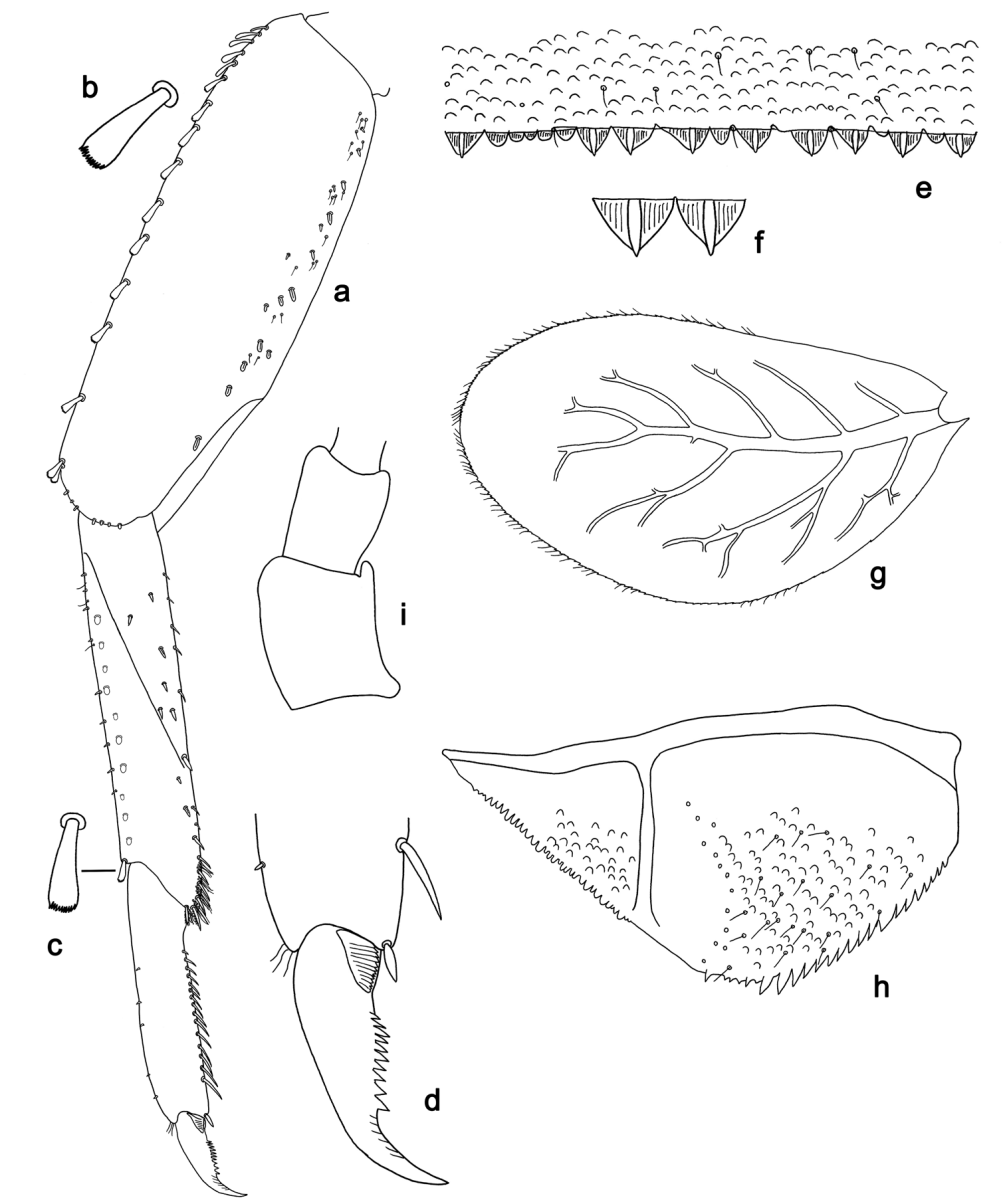
*Labiobaetis gueuningi* sp. nov., larva morphology: **a** Foreleg **b** Fore femur dorsal seta **c** Fore tibia dorsal, apical seta **d** Fore claw **e** Tergum IV **f** Tergum IV, spines at posterior margin **g** Gill IV **h** Paraproct **i** Scape.

Figures 26 , 27 , 49c , 52e , 54a

##### Diagnosis.

**Larva.** Following combination of characters: A) dorsal surface with submarginal arc of 9–10 long, simple setae; B) maxillary palp with three segments; C) labial palp segment II with a broad thumb-like distomedial protuberance, segment III conical; D) fore femur rather broad, length ca. 3× maximum width, dorsal margin with ca. 14 clavate setae, apically with minute serration; E) posterior margin of abdominal tergites with triangular spines, wider than long and with medial enhancement; F) paraproct distally not expanded, with 21–24 stout marginal spines.

##### Description.

**Larva** (Figs 26, 27, 49c, 52e). Body length 5.6 mm. Cerci length ca. 2/3 of body length. Terminal filament: length ca. 3/4 length of cerci.

*Colouration*. Head, thorax, and abdomen dorsally dark brown, head and thorax with bright median, dorsal suture, thorax and abdomen with lively pattern as in Fig. 49c, forewing pads light brown with brown and bright striation. Head, thorax, and abdomen ventrally with pattern as in Fig. 52e, femur with yellowish markings, distomedial brown spot, taupe dorsal margin and taupe apex, tibia and tarsus brown, caudal filaments taupe, with black band at ca. 1/2 of cerci.

*Antenna* with scape and pedicel subcylindrical, with well-developed distolateral process at scape; flagellum with lanceolate spines and fine, simple setae on apex of each segment.

*Labrum* (Fig. 26a). Rectangular, length 0.8× maximum width. Distal margin with medial emargination and a small process. Dorsally with medium, fine, simple setae scattered over surface; submarginal arc of setae composed of nine or ten medium to long, simple setae. Ventrally with marginal row of setae composed of lateral and anterolateral long, feathered setae and medial long, bifid, pectinate setae; ventral surface with four short, spine-like setae near lateral and anterolateral margin.

*Right mandible* (Fig. 26b, c). Incisors fused. Outer and inner sets of denticles with 4 + 3 denticles and one minute intermediate denticle. Inner margin of innermost denticle with a row of thin setae. Prostheca robust, apically denticulate. Margin between prostheca and mola straight. Tuft of setae at apex of mola present.

*Left mandible* (Fig. 26d, e). Incisors fused. Outer and inner sets of denticles with 3 + 3 denticles. Prostheca robust, apically with small denticles and comb-shaped structure. Margin between prostheca and mola straight, with minute denticles towards subtriangular process. Subtriangular process long and slender, above level of area between prostheca and mola. Denticles of mola apically constricted. Tuft of setae at apex of mola present.

Both mandibles with lateral margins almost straight. Basal half with fine, simple setae scattered over dorsal surface.

*Hypopharynx* (Fig. 26f). Lingua approx. as long as superlingua. Lingua approx. as broad as long; medial tuft of stout setae poorly developed; distal half not expanded. Superlingua rounded; lateral margin rounded; fine, long, simple setae along distal margin.

*Maxilla* (Fig. 26g). Galea-lacinia with two simple, robust apical setae under crown. Inner dorsal row of setae with three denti-setae, distal denti-seta tooth-like, middle and proximal denti-setae slender, bifid and pectinate. Medially with one bipectinate, spine-like seta and five medium to long, simple setae. Maxillary palp 1.3× as long as length of galea-lacinia; three segmented; palp segment II approx. as long as segment I; setae on maxillary palp fine, simple, scattered over surface of segments I, II and III; palp segment III shorter than segment II; apex of last segment slightly pointed, without excavation at inner distolateral margin.

*Labium* (Fig. 26h). Glossa basally broad, narrowing toward apex; shorter than paraglossa; inner margin with ten or eleven spine-like setae; apex with two long and one medium, robust, pectinate setae and one short, robust seta; outer margin with 5–7 spine-like setae; ventral surface with short, fine, simple and short, spine-like setae. Paraglossa sub-rectangular, curved inward; apex rounded; with three rows of long, robust, distally pectinate setae in apical area and one or two medium, simple setae in anteromedial area; dorsally with a row of six long, spine-like setae near inner margin. Labial palp with segment I 0,7× length of segments II and III combined. Segment I ventrally with short, fine, simple setae. Segment II with broad thumb-like distomedial protuberance; distomedial protuberance 0.5× width of base of segment III; inner and outer margin with short, fine, simple setae; dorsally with a row of four long, spine-like, simple setae near outer margin. Segment III conical; apex with small projection; length 0.9× width; ventrally covered with short, spine-like, simple setae and short, fine, simple setae.

*Hind wing pads* absent.

*Foreleg* (Fig. 27a, b, c, d). Ratio of foreleg segments 1.4:1.0:0.7:0.3. *Femur*. Length ca. 3× maximum width. Dorsal margin with a row of ca. 14 clavate setae, apically with minute serration; length of setae 0.14× maximum width of femur. Apex rounded; with one pair of clavate setae with apical minute serration and some short, stout setae. Many stout, lanceolate, apically rounded setae scattered along the ventral margin; femoral patch poorly developed. *Tibia*. Dorsal margin with a row of short, stout setae and fine, simple setae, along margin a row of lanceolate, apically rounded setae; on apex one long, clavate seta with minute apical serration. Ventral margin with a row of curved, spine-like setae, on apex some bipectinate, spine-like setae and a tuft of simple setae. Anterior surface scattered with stout, lanceolate setae. Patellotibial suture present on basal 2/3. *Tarsus*. Dorsal margin with a row of short, stout setae, on apex fine, simple setae. Ventral margin with a row of curved, spine-like setae. Tarsal claw with one row of ten or eleven denticles; distally pointed; with 5–6 stripes; subapical setae absent.

*Tergum* (Fig. 27e, f). Surface with rows of U-shaped scale bases and scattered fine, simple setae. Posterior margin of tergum IV with triangular spines, wider than long and with medial enhancement.

*Gills* (Fig. 27g). Present on segments I–VII. Margin with small denticles intercalating fine simple setae. Tracheae extending from main trunk to inner and outer margins. Gill I as long as length of segment II. Gill IV as long as length of segments V and VI combined. Gill VII as long as length of segments VIII and 1/3 IX combined.

*Paraproct* (Fig. 27h). Distally not expanded, with 21–24 stout marginal spines. Surface scattered with U-shaped scale bases, fine, simple setae and micropores. Cercotractor with numerous small marginal spines.

##### Etymology.

Dedicated to Morgan Gueuning, who collected the specimens in Sumatra.

##### Distribution.

Indonesia: Sumatra.

##### Biological aspects.

The specimens were collected at altitudes of 840 m to 1,300 m in small to medium (0.5–5 m wide), shallow (7–40 cm deep) and slow to medium fast (velocity 0.2–0.8 m/s) streams in forest or in highly disturbed areas (agriculture, waste). The substrate was predominantly boulder, stones, gravel and sand with few patches of leaf litter of dead wood.

##### Type-material.

**Holotype.** Larva (on slide, GBIFCH 00422153), Indonesia, Sumatra, volcano Singgalang, River Airjernih, 00°24.12'S, 100°16.73'E, 840 m, 25.III.2014, M. Gueuning leg. Temporary deposited in MZL before definitely housed in MZB. **Paratypes.** 1 larva (in alcohol, GBIFCH 00422220, deposited in MZL), same data as holotype; 12 larvae (3 on slides, GBIFCH 00465243, GBIFCH00465244, GBIFCH 00465245, 8 in alcohol, GBIFCH 00515340, GBIFCH 00422884, GBIFCH 00422224, GBIFCH 00422195, GBIFCH 00422200, GBIFCH 00422190, GBIFCH 00422197, GBIFCH 00422793, deposited in MZL), Indonesia, Sumatra, volcano Singgalang, River Sianok, 00°19.95'S, 100°19.32'E, 1,150 m, 24.III.2014, M. Gueuning leg.; 1 larva (on slide, GBIFCH 00422205, deposited in MZL), Indonesia, Sumatra, volcano Sago, River Kaligain, 00°18.02'S, 100°40.13'E, 1,040 m, 05.IV.2014, M. Gueuning leg.; 2 larvae (in alcohol, GBIFCH 00422156, GBIFCH 00422114, deposited in MZL), Indonesia, Sumatra, volcano Singgalang, River Caruak, 00°22.93'S, 100°22.70'E, 1,300 m, 23.III.2014, M. Gueuning leg.; 5 larvae (in alcohol, GBIFCH 00422149, GBIFCH 00422147, GBIFCH 00422163, GBIFCH 00422182, GBIFCH 00422141, deposited in ZSM), Indonesia, Sumatra, volcano Singgalang, River Magyih, 00°23.55'S, 100°16.57'E, 845 m, 25.III.2014, M. Gueuning leg.; 1 larva (in alcohol, GBIFCH 00422108, deposited in ZSM), Indonesia, Sumatra, volcano Singgalang, River Magyih, 00°22.85'S, 100°17.65'E, 1,075 m, 26.III.2014, M. Gueuning leg.

#### 16. Labiobaetis minang sp. nov.

Taxon classificationAnimaliaEphemeropteraBaetidae

C33411A0-A382-5B19-A32F-6B298BDEF322

http://zoobank.org/650FDA3E-4800-488F-A394-1482E2A9EE20

**Figure 28.**
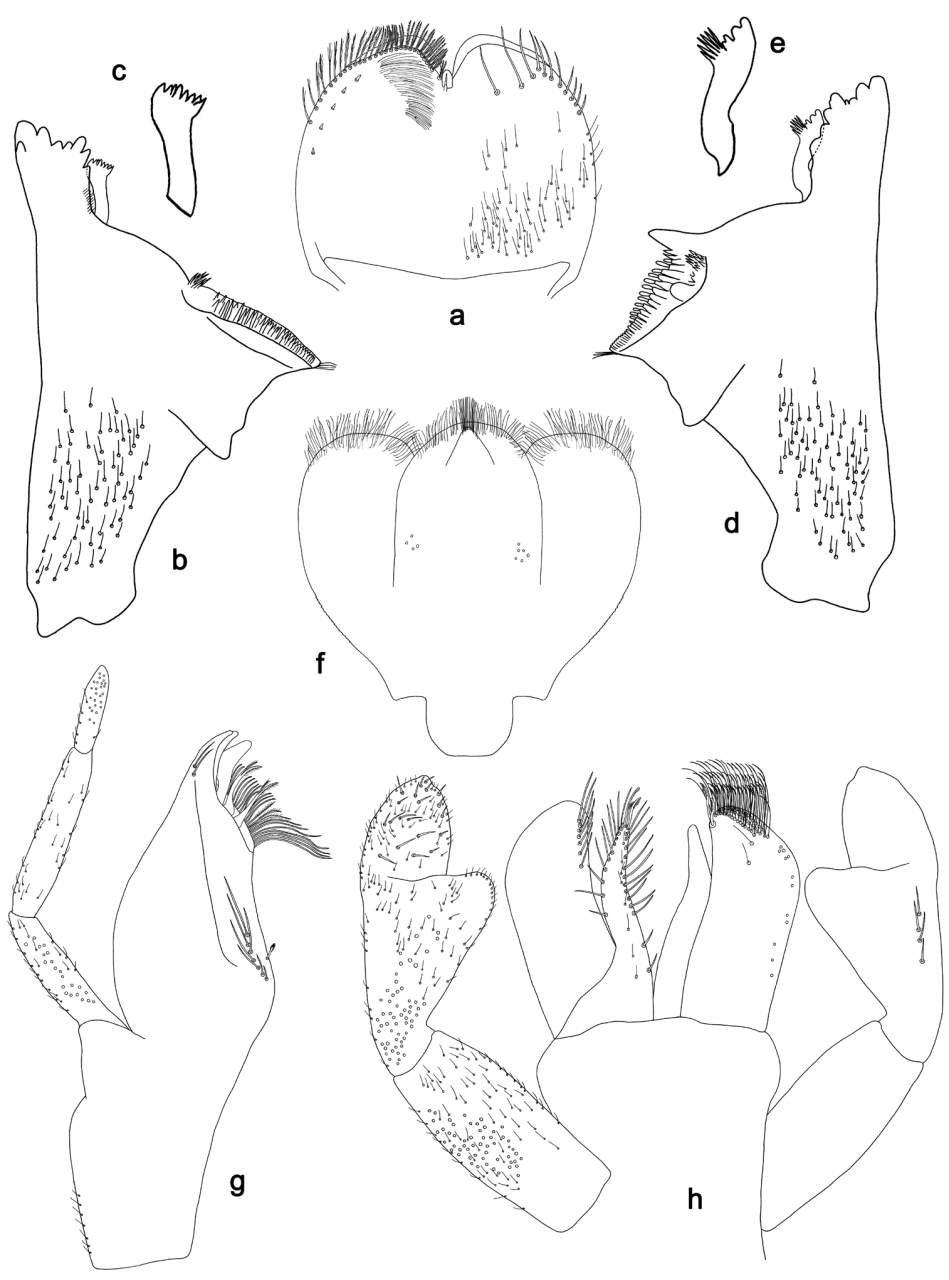
*Labiobaetis minang* sp. nov., larva morphology: **a** Labrum **b** Right mandible **c** Right prostheca **d** Left mandible **e** Left prostheca **f**Hypopharynx**g** Maxilla **h** Labium.

**Figure 29.**
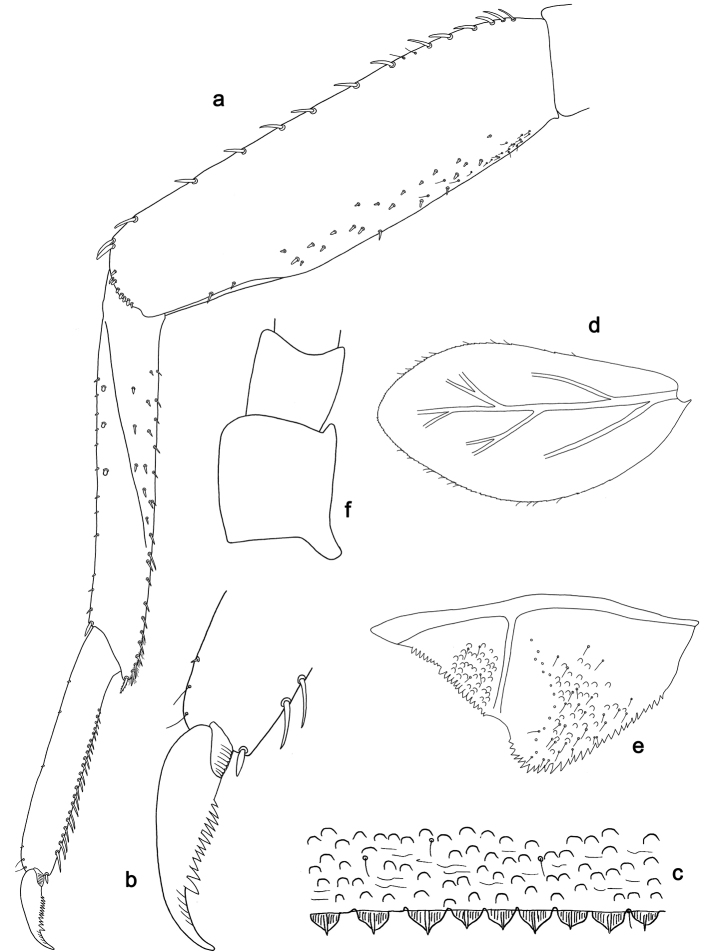
*Labiobaetis minang* sp. nov., larva morphology: **a** Foreleg **b** Fore claw **c** Tergum IV **d** Gill IV **e** Paraproct **f** Scape.

Figures 28 , 29 , 49d , 55a

##### Diagnosis.

**Larva.** Following combination of characters: A) dorsal surface of labrum with submarginal arc of eleven medium to long, simple setae; B) maxillary palp with three segments; C) labial palp segment II with broad thumb-like distomedial protuberance, segment III oblong; D) fore femur rather slender, length 3.6× maximum width, dorsal margin with 12–15 spine-like setae; E) posterior margin of abdominal tergites with triangular spines, wider than long and with medial enhancements; F) paraproct distally not expanded, with ca. 29 stout marginal spines.

##### Description.

**Larva** (Figs 28, 29, 49d). Body length 7.1 mm. Cerci length ca. 2/3 of body length.

*Colouration*. Colouration of head, thorax, and abdomen unknown due to DNA extraction from all specimens in an earlier project. Femur along dorsal and ventral margin brown, tibia and tarsus brown.

*Antenna* with scape and pedicel subcylindrical, with well-developed distolateral process at scape; flagellum with lanceolate spines and fine, simple setae on apex of each segment.

*Labrum* (Fig. 28a). Rectangular, length 0.8× maximum width. Distal margin with medial emargination and a small process. Dorsally with medium, fine, simple setae scattered over surface; submarginal arc of setae composed of eleven medium to long, simple setae. Ventrally with marginal row of setae composed of lateral and anterolateral long, feathered setae and medial long, bifid, pectinate setae; ventral surface with five short, spine-like setae near lateral and anterolateral margin.

*Right mandible* (Fig. 28b, c). Incisors fused. Outer and inner sets of denticles with 4 + 3 denticles and one minute intermediate denticle. Inner margin of innermost denticle with a row of thin setae. Prostheca robust, apically denticulate. Margin between prostheca and mola straight. Tuft of setae at apex of mola present.

*Left mandible* (Fig. 28d, e). Incisors fused. Outer and inner sets of denticles with 3 + 4 denticles and one minute intermediate denticle. Prostheca robust, apically with small denticles and comb-shaped structure. Margin between prostheca and mola straight. Subtriangular process long and slender, above level of area between prostheca and mola. Denticles of mola apically constricted. Tuft of setae at apex of mola present.

Both mandibles with lateral margins almost straight. Basal half with fine, simple setae scattered over dorsal surface.

*Hypopharynx* (Fig. 28f). Lingua approx. as long as superlingua. Lingua longer than broad; medial tuft of stout setae poorly developed; distal half not expanded. Superlingua rounded; lateral margin rounded; fine, long, simple setae along distal margin.

*Maxilla* (Fig. 28g). Galea-lacinia with two simple, robust apical setae under crown. Inner dorsal row of setae with three denti-setae, distal denti-seta tooth-like, middle and proximal denti-setae slender, bifid and pectinate. Medially with one bipectinate, spine-like seta and seven or eight simple setae increasing in length distally. Maxillary palp 1.3× as long as length of galea-lacinia; three segmented; palp segment II 1.4× length of segment I; setae on maxillary palp fine, simple, scattered over surface of segments I, II and III; palp segment III shorter than segment II; apex of last segment slightly pointed, without excavation at inner distolateral margin.

*Labium* (Fig. 28h). Glossa basally broad, narrowing toward apex; shorter than paraglossa; inner margin with 13 spine-like setae increasing in length distally; apex with two long and one medium, robust, pectinate setae; outer margin with ten or eleven spine-like setae; ventral surface with short, fine, simple setae. Paraglossa sub-rectangular, curved inward; apex rounded; with three rows of long, robust, distally pectinate setae in apical area and two medium, simple setae in anteromedial area; dorsally with a row of seven or eight long, spine-like setae near inner margin. Labial palp with segment I 0.8× length of segments II and III combined. Segment I ventrally with short, fine, simple setae. Segment II with broad thumb-like distomedial protuberance; distomedial protuberance 0.6× width of base of segment III; inner and outer margin with short, fine, simple setae; dorsally with a row of 4–6 medium, spine-like setae near outer margin. Segment III oblong; apex with small projection; length 1.3× width; ventrally covered with short and medium spine-like, simple setae and short, fine, simple setae.

*Hind wing pads* absent.

*Foreleg* (Fig. 29a, b). Ratio of foreleg segments 1.3:1.0:0.7:0.2. *Femur*. Length ca. 4× maximum width. Dorsal margin with a row of 12–15 curved, spine-like setae; length of setae 0.16× maximum width of femur. Apex rounded; with one pair of curved, spine-like setae and some short, stout setae. Many stout, lanceolate, apically rounded setae scattered along the ventral margin; femoral patch well developed. *Tibia.* Dorsal margin with a row of short, stout setae and a few lanceolate, apically rounded setae along margin; on apex one longer, spine-like seta. Ventral margin with a row of curved, spine-like setae, on apex one bipectinate, spine-like seta and a tuft of fine, simple setae. Anterior surface scattered with stout, lanceolate setae. Patellotibial suture present on basal 2/3. *Tarsus*. Dorsal margin with a row of short, stout setae, on apex fine, simple setae. Ventral margin with a row of curved, spine-like setae. Tarsal claw with one row of 12 denticles; distally pointed; with five stripes; subapical setae absent.

*Tergum* (Fig. 29c). Surface with rows of U-shaped scale bases and scattered fine, simple setae. Posterior margin of tergum IV with triangular spines, wider than long and with medial enhancement.

*Gills* (Fig. 29d). Present on segments I-VII. Margin with small denticles intercalating fine simple setae. Tracheae extending from main trunk to inner and outer margins. Gill I as long as length of segment II. Gill IV as long as length of segments V and 1/2 VI combined. Gill VII as long as length of segments VIII and 1/3 IX combined.

*Paraproct* (Fig. 29e). Distally not expanded with ca. 29 stout marginal spines. Surface scattered with U-shaped scale bases, fine, simple setae and micropores. Cercotractor with numerous small marginal spines.

##### Etymology.

Dedicated to the indigenous Minang people from Sumatra.

##### Distribution.

Indonesia: Sumatra.

##### Biological aspects.

The specimens were collected in altitudes of 1,640 m a.s.l. and 1,785 m a.s.l. in small (ca. 1 m wide), shallow (ca. 15 cm deep) and slow (0.15 m/s – 0.2 m/s) forest streams with partly open or closed canopy. The substrate was predominantly boulder, stones and gravel with patches of leaf litter or dead wood.

##### Type-material.

**Holotype.** Larva (on slide, GBIFCH 00422498), Indonesia, Sumatra, Volcan Singgalang, River Caruak, 00°23.05'S, 100°21.40'E, 1,640 m, 23.III.2014, M. Gueuning leg. Temporary deposited in MZL before definitely housed in MZB. **Paratypes.** 3 larvae (2 on slides, GBIFCH 00422537, GBIFCH 00422456, 1 in alcohol, GBIFCH 00422521, deposited in MZL), Indonesia, Sumatra, Volcan Singgalang, River Pagu Pagu, 00°23.53'S, 100°21.45'E, 1,785 m, 22.III.2014, M. Gueuning leg.

### *Labiobaetis numeratus* group of species

With the following combination of characters: A) dorsal surface with submarginal arc of simple setae; B) labial palp segment II with thumb-like distomedial protuberance; C) right mandible with a pronounced hump between prostheca and mola; D) six pairs of gills; E) hindwing pads present, minute; F) distolateral process at scape absent.

#### 17. Labiobaetis numeratus

Taxon classificationAnimaliaEphemeropteraBaetidae

(Müller-Liebenau, 1984)

0CB9F344-BCE4-544F-85FD-60D689382593

Figure 30 , 53b

##### Diagnosis.

**Larva.** Following combination of characters: A) dorsal surface of labrum with submarginal arc of 1 + ca. 7 simple setae; B) labial palp segment II with a large, thumb-like distomedial protuberance; segment III conical, apically rounded; C) right mandible with a pronounced hump between prostheca and mola; D) hypopharynx with medial tuft of stout setae well developed; E) maxillary palp much longer than length of galea-lacinia; F) fore femur rather broad, length ca. 3× maximum width, dorsal margin with ca. seven curved, spine-like setae; G) tarsal claw with 10–12 denticles; H) paraproct distally not expanded, with 12–14 stout marginal spines.

##### Examined material.

**Paratype.** 1 larva (on slide, no. 40), W. Malaysia, Gombak River, 4½ miles N of Kuala Lumpur, 19.X.[19]68, Bishop leg.

**Figure 30.**
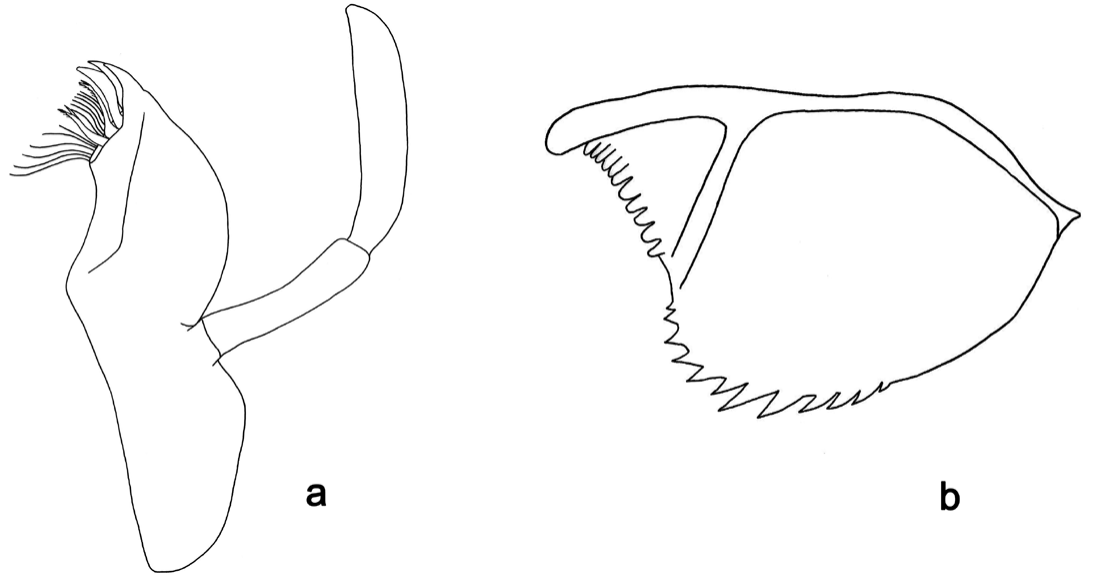
*Labiobaetis numeratus*, larva morphology: **a** Maxilla **b** Paraproct.

#### 18. Labiobaetis paranumeratus sp. nov.

Taxon classificationAnimaliaEphemeropteraBaetidae

3A3367E7-CDB4-5F86-8FBA-4982BAD5A296

http://zoobank.org/551F3B4B-4F90-4433-A26A-8E305E6A3024

**Figure 31.**
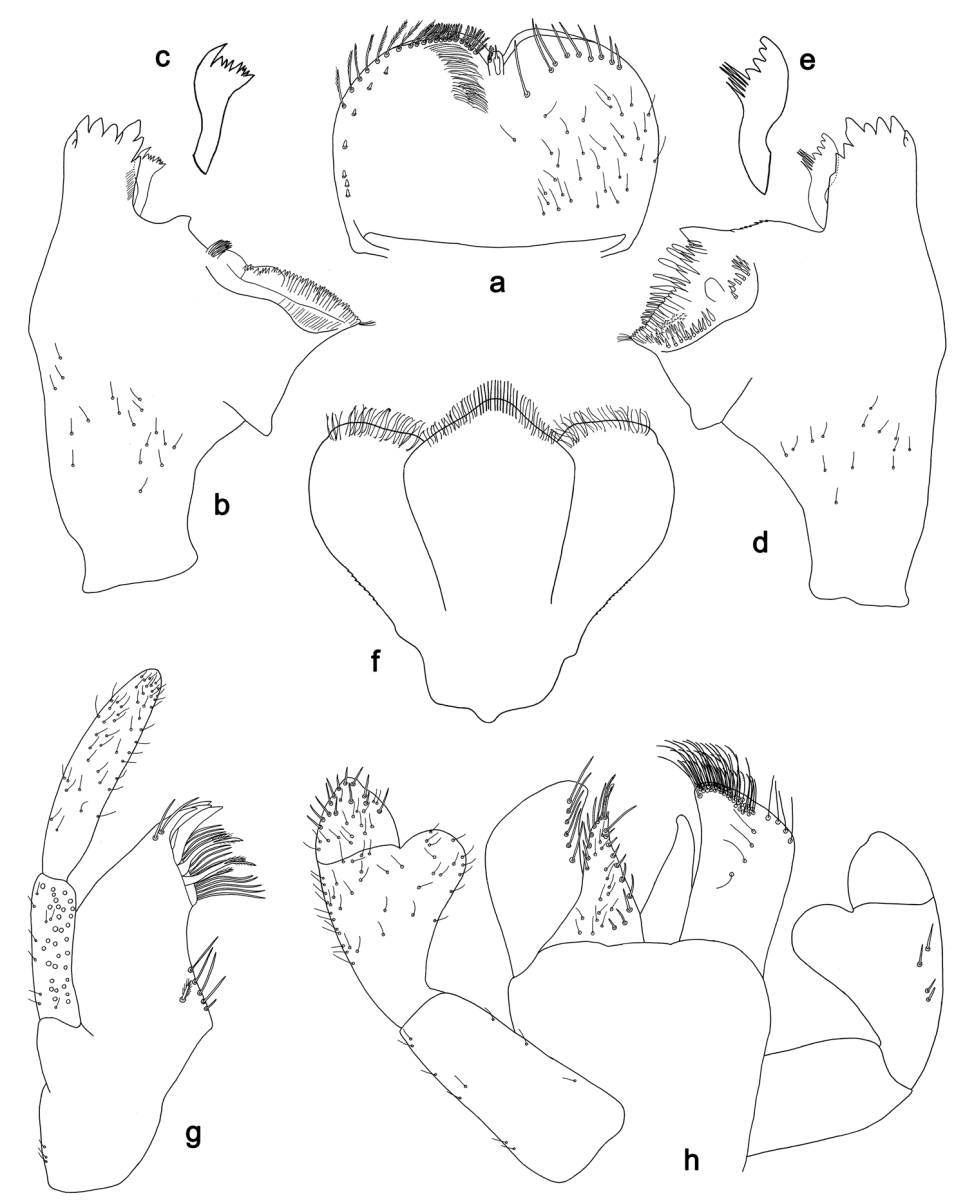
*Labiobaetis paranumeratus* sp. nov., larva morphology: **a** Labrum **b** Right mandible **c** Right prostheca **d** Left mandible **e** Left prostheca **f**Hypopharynx**g** Maxilla **h** Labium.

**Figure 32.**
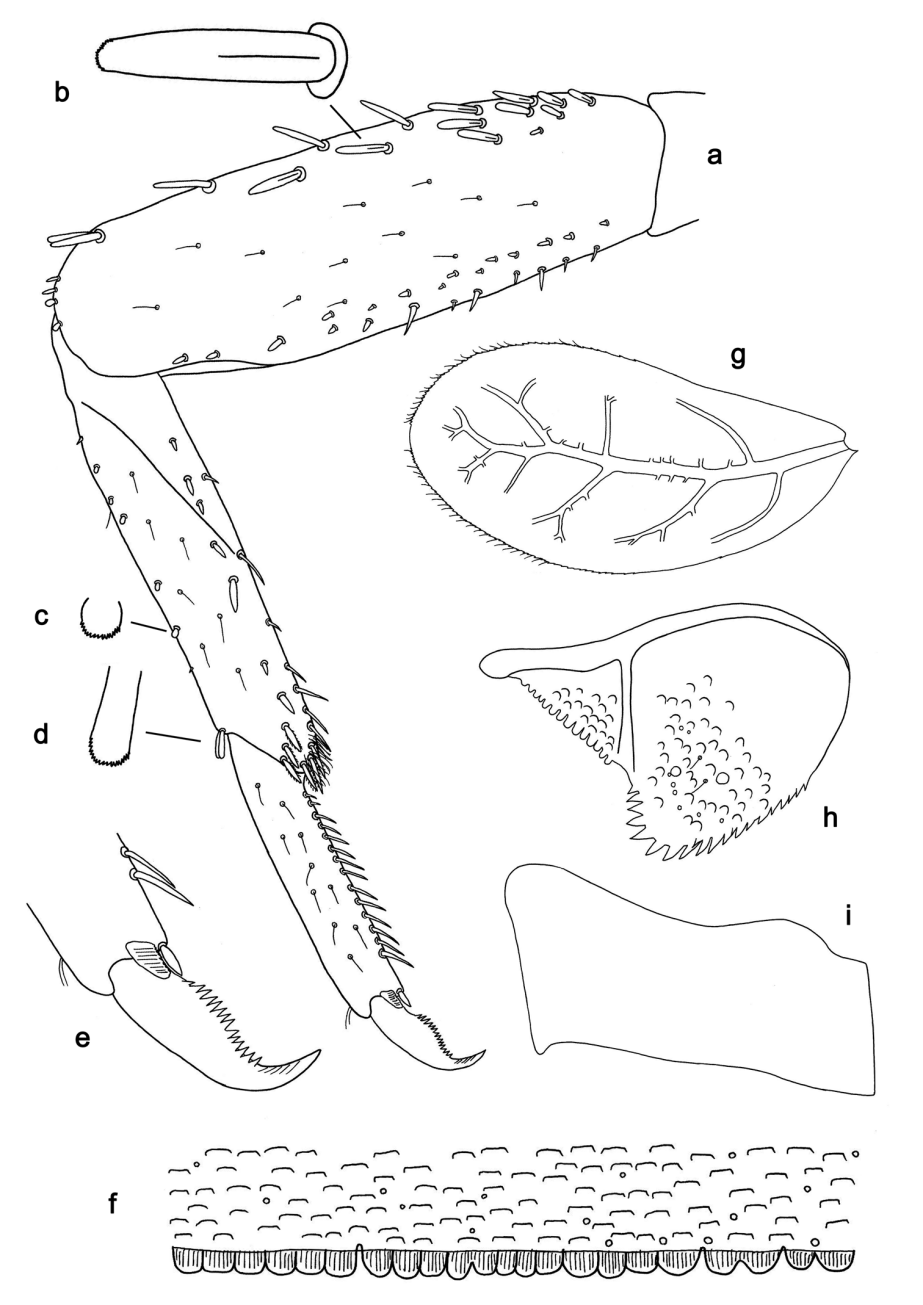
*Labiobaetis paranumeratus* sp. nov., larva morphology: **a** Foreleg **b** Fore femur dorsal seta **c** Fore tibia dorsal seta **d** Fore tibia dorsal, apical seta **e** Fore claw **f** Tergum IV **g** Gill IV **h** Paraproct **i** Metanotum.

Figures 31 , 32 , 50a , 54b

##### Diagnosis.

**Larva.** Following combination of characters: A) dorsal surface of labrum with submarginal arc of 1 + 8 medium to long, simple setae, the first two setae after the central seta are closely together; B) right mandible with a pronounced hump between prostheca and mola; C) labial palp segment II with a broad thumb-like distomedial protuberance, segment III conical; D) fore femur rather broad, length ca. 3× maximum width, dorsal margin with ca. seven spine-like setae, apically rounded and with minute serration, close to margin some spine-like setae, apically rounded and with minute pectination; E) paraproct distally not expanded, with ca. 17 stout marginal spines and some submarginal spines.

##### Description.

**Larva** (Figs 31, 32, 50a). Body length 4.3–4.7 mm. Cerci ca. 2/3 length of abdomen. Terminal filament: approx. as long as cerci.

*Colouration*. Head, thorax, and abdomen dorsally brown, head and thorax with bright median, dorsal suture, forewing pads light brown with darker striation. Head, thorax, and abdomen ventrally brown, legs transparent, femur distomedially with a brown spot, tibia distomedially and tarsus proximally brown. Caudal filaments light brown, with a dark brown band at 2/3 of cerci.

*Antenna* with scape and pedicel subcylindrical, without distolateral process at scape; flagellum with broad, apically blunt spines and fine, simple setae on apex of each segment.

*Labrum* (Fig. 31a). Rectangular, length 0.7× maximum width. Distal margin with medial emargination and a small process. Dorsally with medium, fine, simple setae scattered over surface; submarginal arc of setae composed of 1 + 8 medium to long, simple setae. Ventrally with marginal row of setae composed of lateral and anterolateral long, feathered setae and medial long, bifid, pectinate setae; ventral surface with seven short, spine-like setae near lateral and anterolateral margin.

*Right mandible* (Fig. 31b, c). Incisors fused. Outer and inner sets of denticles with 4 + 3 denticles and one minute intermediate denticle. Inner margin of innermost denticle with a row of thin setae. Prostheca robust, apically denticulate. Margin between prostheca and mola with a pronounced hump. Tuft of setae at apex of mola present.

*Left mandible* (Fig. 31d, e). Incisors fused. Outer and inner sets of denticles with 4 + 3 denticles. Prostheca robust, apically with small denticles and comb-shaped structure. Margin between prostheca and mola slightly convex, with minute denticles toward subtriangular process. Subtriangular process long and slender, above level of area between prostheca and mola. Denticles of mola apically constricted. Tuft of setae at apex of mola present.

Both mandibles with lateral margins slightly convex. Basal half with fine, simple setae scattered over dorsal surface.

*Hypopharynx* (Fig. 31f). Lingua longer than superlingua. Lingua longer than broad; medial tuft of stout setae poorly developed; distal half laterally expanded. Superlingua straight; lateral margin rounded; fine, long, simple setae along distal margin.

*Maxilla* (Fig. 31g). Galea-lacinia with two simple, robust apical setae under crown. Inner dorsal row of setae with three denti-setae, distal denti-seta tooth-like, middle and proximal denti-setae slender, bifid and pectinate. Medially with one bipectinate, spine-like seta and four or five long, simple setae. Maxillary palp 1.6× as long as length of galea-lacinia; two segmented; palp segment II 1.6× length of segment I; setae on maxillary palp fine, simple, scattered over surface of segments I and II; apex of last segment rounded, without excavation at inner distolateral margin.

*Labium* (Fig. 31h). Glossa basally broad, narrowing toward apex; shorter than paraglossa; inner margin with eight spine-like setae, distalmost seta much longer than other setae; apex with two long and one medium, robust, pectinate setae and one short, robust seta; outer margin with six long spine-like setae increasing in length distally; ventral surface with short, fine, simple and short, spine-like setae. Paraglossa sub-rectangular, curved inward; apex rounded; with three rows of long, robust, distally pectinate setae in apical area and four medium, simple setae in anteromedial area; dorsally with a row of six long, spine-like setae near inner margin. Labial palp with segment I 0.9× length of segments II and III combined. Segment I ventrally with short, fine, simple setae. Segment II with broad thumb-like distomedial protuberance; distomedial protuberance 0.8× width of base of segment III; inner and outer margin with short, fine, simple setae; dorsally with a row of four short to medium, spine-like, simple setae near outer margin. Segment III conical; apex rounded; length 0.9× width; ventrally covered with short and medium spine-like, simple setae and short, fine, simple setae.

*Hind wing pads* minute.

*Foreleg* (Fig. 32a, b, c, d, e). Ratio of foreleg segments 1.3:1.0:0.6:0.2. *Femur*. Length ca. 3× maximum width. Dorsal margin with a row of ca. seven spine-like setae, apically rounded and with minute serration, close to margin some spine-like setae, apically rounded and with minute serration; length of setae 0.27× maximum width of femur. Apex rounded; with one pair of spine-like setae, apically rounded and with minute pectination and with some short, stout setae. Many stout, lanceolate setae scattered along the ventral margin; femoral patch absent. *Tibia*. Dorsal margin with a row of stout setae, apically rounded and with minute pectination; on apex one pair of long, stout setae, apically rounded and with minute pectination. Ventral margin with a row of curved, spine-like setae, on apex some spine-like, bipectinate setae and a tuft of fine, simple setae. Anterior surface scattered with stout, lanceolate setae. Patellotibial suture present on basal 1/2. *Tarsus*. Dorsal margin almost bare. Ventral margin with a row of curved, spine-like setae. Tarsal claw with one row of 13–14 denticles; distally pointed; with five stripes; subapical setae absent.

*Tergum* (Fig. 32f). Surface with irregular rows of scale bases and scattered micropores. Posterior margin of tergum IV with rounded spines, wider than long.

*Gills* (Fig. 32g). Present on segments II–VII. Margin with small denticles intercalating fine simple setae. Tracheae extending from main trunk to inner and outer margins. Gill IV as long as length of segments V and 2/3 VI combined. Gill VII as long as length of segments VIII and 2/3 IX combined.

*Paraproct* (Fig. 32h). Distally not expanded, with 16–19 stout marginal spines and some submarginal spines. Surface scattered with U-shaped scale bases, fine, simple setae and micropores. Cercotractor with numerous small marginal spines.

##### Etymology.

Refers to the morphological similarity with *L. numeratus*.

##### Distribution.

Indonesia: Sumatra.

##### Biological aspects.

The specimens were collected at an altitude of 520 m.

##### Type-material.

**Holotype.** Larva (on slide, GBIFCH 00592210), Indonesia, Sumatra Barat, Harau Canyon, stream near Ikbal's cottage, 520 m, 23.VI.2012, 00°06.44'S, 100°40.37'E, M. Balke leg. (UN11). Temporary deposited in MZL before definitely housed in MZB. **Paratypes.** 2 larvae (on slides, GBIFCH 00592211, GBIFCH 00592212, deposited in MZL), same data as holotype.

#### 19. Labiobaetis pilosus sp. nov.

Taxon classificationAnimaliaEphemeropteraBaetidae

6290BBEF-42C0-5964-B489-BADF48177E1F

http://zoobank.org/F70202C1-5909-48A1-A108-4481165F451E

**Figure 33.**
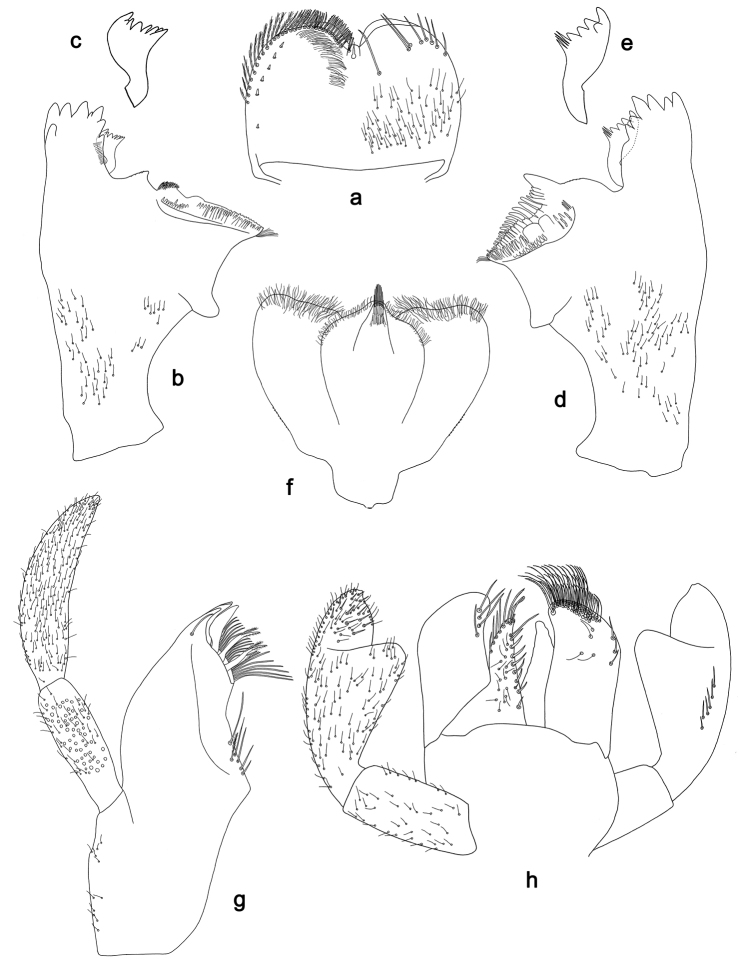
*Labiobaetis pilosus* sp. nov., larva morphology: **a** Labrum **b** Right mandible **c** Right prostheca **d** Left mandible **e** Left prostheca **f**Hypopharynx**g** Maxilla **h** Labium.

**Figure 34.**
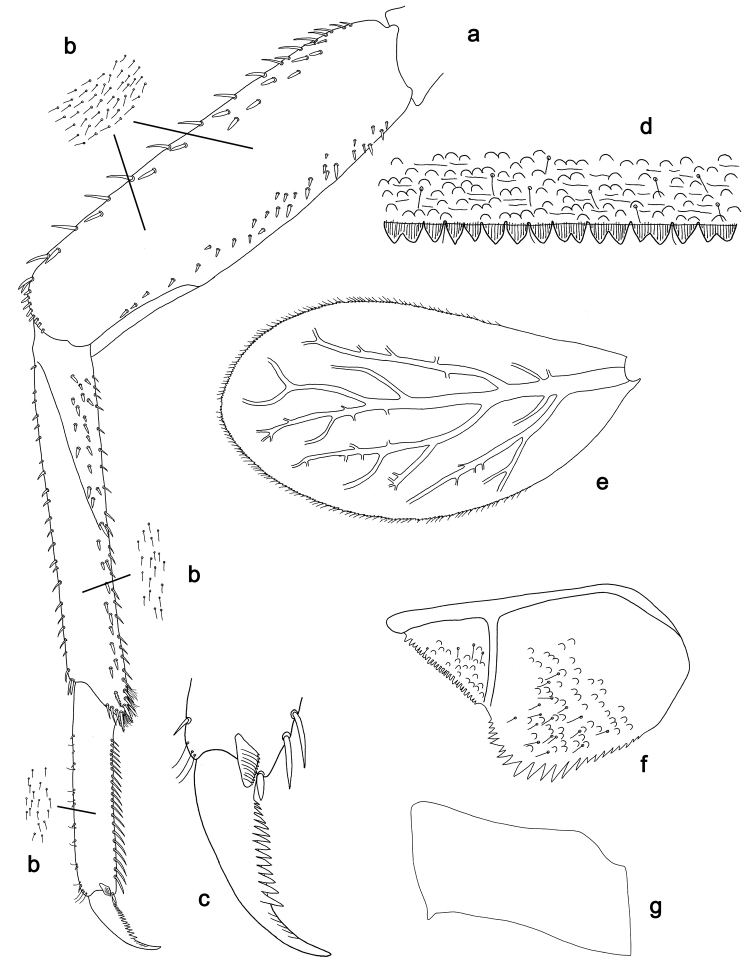
*Labiobaetis pilosus* sp. nov., larva morphology: **a** Foreleg **b** Foreleg setation on surface of femur, tibia and tarsus **c** Fore claw **d** Tergum IV **e** Gill IV **f** Paraproct **g** Metanotum.

Figures 33 , 34 , 50b , 54b

##### Diagnosis.

**Larva.** Following combination of characters: A) dorsal surface of labrum with submarginal arc of 1 + 6–8 long, simple setae, the first two setae after the central seta are closely together; B) right mandible with a pronounced hump between prostheca and mola; C) labial palp segment II with a thumb-like distomedial protuberance, segment III conical; D) maxillary palp segment II large and bent inwards, much longer than segment I; E) fore femur rather broad, length ca. 3.4× width, dorsal margin with a row of 12–16 curved, spine-like setae and many spine-like apically rounded setae along margin; F) paraproct distally not expanded, with ca. 21 stout marginal spines.

##### Description.

**Larva** (Figs 33, 34, 50b). Body length 6.7 mm; antenna approximately 2.5× as long as head length.

*Colouration.* Head, thorax, and abdomen dorsally brown, head and thorax with bright median, dorsal suture, abdominal segments with pattern as in Fig. 50b, forewing pads with bright striation. Head, thorax, and abdomen ventrally brown, abdominal segment X light brown, legs light brown, dorsal margin of femur brown, caudal filaments light brown and with a dark brown band at 1/3 of cerci.

*Antenna* with scape and pedicel subcylindrical, without distolateral process at scape; flagellum with lanceolate spines and fine, simple setae on apex of each segment.

*Labrum* (Fig. 33a). Rectangular, length 0.7× maximum width. Distal margin with medial emargination and a small process. Dorsally with medium, fine, simple setae scattered over surface; submarginal arc of setae composed of 1 + 6–8 long, simple setae. Ventrally with marginal row of setae composed of lateral and anterolateral long, feathered setae and medial long, bifid, pectinate setae; ventral surface with six short, spine-like setae near lateral and anterolateral margin.

*Right mandible* (Fig. 33b, c). Incisors fused. Outer and inner sets of denticles with 4 + 3 denticles and one minute intermediate denticle. Inner margin of innermost denticle with a row of thin setae. Prostheca robust, apically denticulate. Margin between prostheca and mola with a pronounced hump and minute denticles on margin of hump. Tuft of setae at apex of mola present.

*Left mandible* (Fig. 33d, e). Incisors fused. Outer and inner sets of denticles with 3 + 3 denticles. Prostheca robust, apically with small denticles and comb-shaped structure. Margin between prostheca and mola slightly convex, with minute denticles toward subtriangular process. Subtriangular process long and slender, above level of area between prostheca and mola. Denticles of mola apically constricted. Tuft of setae at apex of mola present.

Both mandibles with lateral margins almost straight. Basal half with fine, simple setae scattered over dorsal surface.

*Hypopharynx* (Fig. 33f). Lingua approx. as long as superlingua. Lingua longer than broad; medial tuft of stout setae well developed; distal half laterally expanded. Superlingua straight; lateral margin rounded; fine, long, simple setae along distal margin.

*Maxilla* (Fig. 33g). Galea-lacinia with one simple, robust apical seta under crown. Inner dorsal row of setae with three denti-setae, distal denti-seta tooth-like, middle and proximal denti-setae slender, bifid and pectinate. Medially with one bipectinate, spine-like seta and five long, simple setae. Maxillary palp 1.6× as long as length of galea-lacinia; two segmented; palp segment II 1.4× length of segment I; setae on maxillary palp fine, simple, scattered over surface of segments I and II, very dense on segment II; apex of last segment slightly pointed, with excavation at inner distolateral margin.

*Labium* (Fig. 33h). Glossa basally broad, narrowing toward apex; shorter than paraglossa; inner margin with nine or ten spine-like setae, distalmost seta much longer and less robust than other setae; apex with two long and one medium, robust, pectinate setae and one short, robust seta; outer margin with 5–7 spine-like setae increasing in length distally; ventral surface with short, fine, simple, scattered setae. Paraglossa sub-rectangular, curved inward; apex rounded; with three rows of long, robust, distally pectinate setae in apical area; outer margin with row of three long, spine-like setae and 4–7 medium, simple setae in anteromedial area; dorsally with a row of 4–5 long, spine-like setae near inner margin. Labial palp with segment I 0.6× length of segments II and III combined. Segment I ventrally with short, fine, simple setae. Segment II with thumb-like distomedial protuberance; distomedial protuberance 0.6× width of base of segment III; inner and outer margin with short, fine, simple setae; dorsally with a row of 5–7 medium, spine-like, simple setae near outer margin. Segment III conical; apex slightly truncate; length 1.1× width; ventrally covered with short, spine-like, simple setae and short, fine, simple setae.

*Hind wing pads* minute.

*Foreleg* (Fig. 34a–c). Ratio of foreleg segments 1.2:1.0:0.5:0.2. *Femur*. Length ca. 3× maximum width. Dorsal margin with a row of 12–16 curved, spine-like setae and many spine-like, apically rounded setae along margin; length of setae 0.19× maximum width of femur. Apex rounded; with one pair of curved, spine-like setae and some short, stout setae. Many stout, lanceolate setae scattered along the ventral margin, femoral patch absent. Anterior surface covered with fine, simple setae. *Tibia*. Dorsal margin with a row of curved, spine-like setae; on apex a pair of longer, spine-like setae. Ventral margin with a row of curved, spine-like setae, on apex several stout, partly bipectinate setae and a tuft of fine, simple setae. Anterior surface scattered with stout, lanceolate setae and covered with fine, simple setae. Patellotibial suture present on basal 1/2. *Tarsus*. Dorsal margin with a row of short, spine-like setae and fine, simple setae. Ventral margin with a row of curved, spine-like setae. Anterior surface covered with fine, simple setae. Tarsal claw with one row of 11–14 denticles; distally pointed; with five stripes; subapical setae absent.

*Tergum* (Fig. 34d). Surface with irregular rows of U-shaped scale bases and scattered fine, simple setae. Posterior margin of tergum IV with triangular spines, approx. as long as wide.

*Gills* (Fig. 34e). Present on segments II–VII. Margin with small denticles intercalating fine simple setae. Tracheae extending from main trunk to inner and outer margins. Gill IV as long as length of segments V and 2/3 VI combined. Gill VII as long as length of segments VIII and 1/3 IX combined.

*Paraproct* (Fig. 34f). Distally not expanded, with 18–23 stout marginal spines. Surface scattered with U-shaped scale bases and fine, simple setae. Cercotractor with numerous small marginal spines.

##### Etymology.

Refers to the many fine, dense hairs on the legs and the mouthparts.

##### Distribution.

Indonesia: Sulawesi.

##### Biological aspects.

The specimens were collected at altitudes of 660 m and 1,600 m.

##### Type-material.

**Holotype.** Larva (on slide, GBIFCH 00592201), Indonesia, Sulawesi Tengah, Lake Lore, 1,600 m, 01.IX.2011, 01°19.58'S, 120°18.67'E, M. Balke leg. (SUL013). Temporary deposited in MZL before definitely housed in MZB. **Paratypes.** 31 larvae (1 on slide, GBIFCH 00592202, 28 in alcohol, GBIFCH 00515359, GBIFCH 00657738, GBIFCH 00657734, deposited in MZL; 2 in alcohol, GBIFCH 00515355, GBIFCH 00515356, deposited in ZSM), same data as holotype; 7 larvae (3 on slides, GBIFCH 00592203, GBIFCH 00592204, GBIFCH 00592205, deposited in MZL; 4 in alcohol, GBIFCH 00515357, GBIFCH 00515358, deposited in ZSM), Indonesia, Sulawesi Tengah, Palu-Lake Lore, stream, 660 m, 01.IX.2011, 01°11.74'S, 120°10.20'E, M. Balke leg. (SUL012).

### *Labiobaetis operosus* group of species

With the following combination of characters: A) dorsal surface of labrum with submarginal arc of feathered setae; B) labial palp segment II with thumb-like distomedial protuberance; C) seven pairs of gills; D) hindwing pads well developed; E) distolateral process at scape well developed.

#### 20. Labiobaetis operosus

Taxon classificationAnimaliaEphemeropteraBaetidae

(Müller-Liebenau, 1984)

1CFC6686-B8D0-5B19-A12D-5D83FA8B503F

Figures 35 , 53b

##### Diagnosis.

**Larva.** Following combination of characters: A) dorsal surface of labrum with submarginal arc of ca. ten feathered setae; B) labial palp segment II with thumb-like distomedial protuberance, segment III slightly pentagonal, apically slightly truncate; C) hypopharynx with medial tuft of stout setae well developed; D) fore femur rather slender, length 3.8× maximum width, dorsal margin with a row of ca. ten spine-like setae; E) paraproct distally not expanded, with ca. 16 stout marginal spines.

##### Examined material.

**Holotype.** 1 larva (on slide, no. 34), W. Malaysia, Gombak River, 4½ miles N of Kuala Lumpur, nr. Bentong road, 18.IX.1969, Bishop leg.

**Figure 35.**
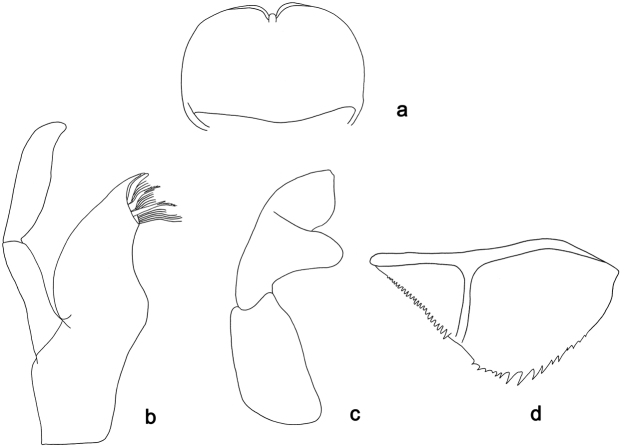
*Labiobaetis operosus*, larva morphology: **a** Labrum **b** Maxilla **c** Labial palp **d** Paraproct.

#### 21. Labiobaetis paraoperosus sp. nov.

Taxon classificationAnimaliaEphemeropteraBaetidae

1A70DFCF-68FC-59AB-8AF6-8B6C28A4E5CF

http://zoobank.org/7CC2BD0A-1543-4570-A81D-CE4189B8A945

**Figure 36.**
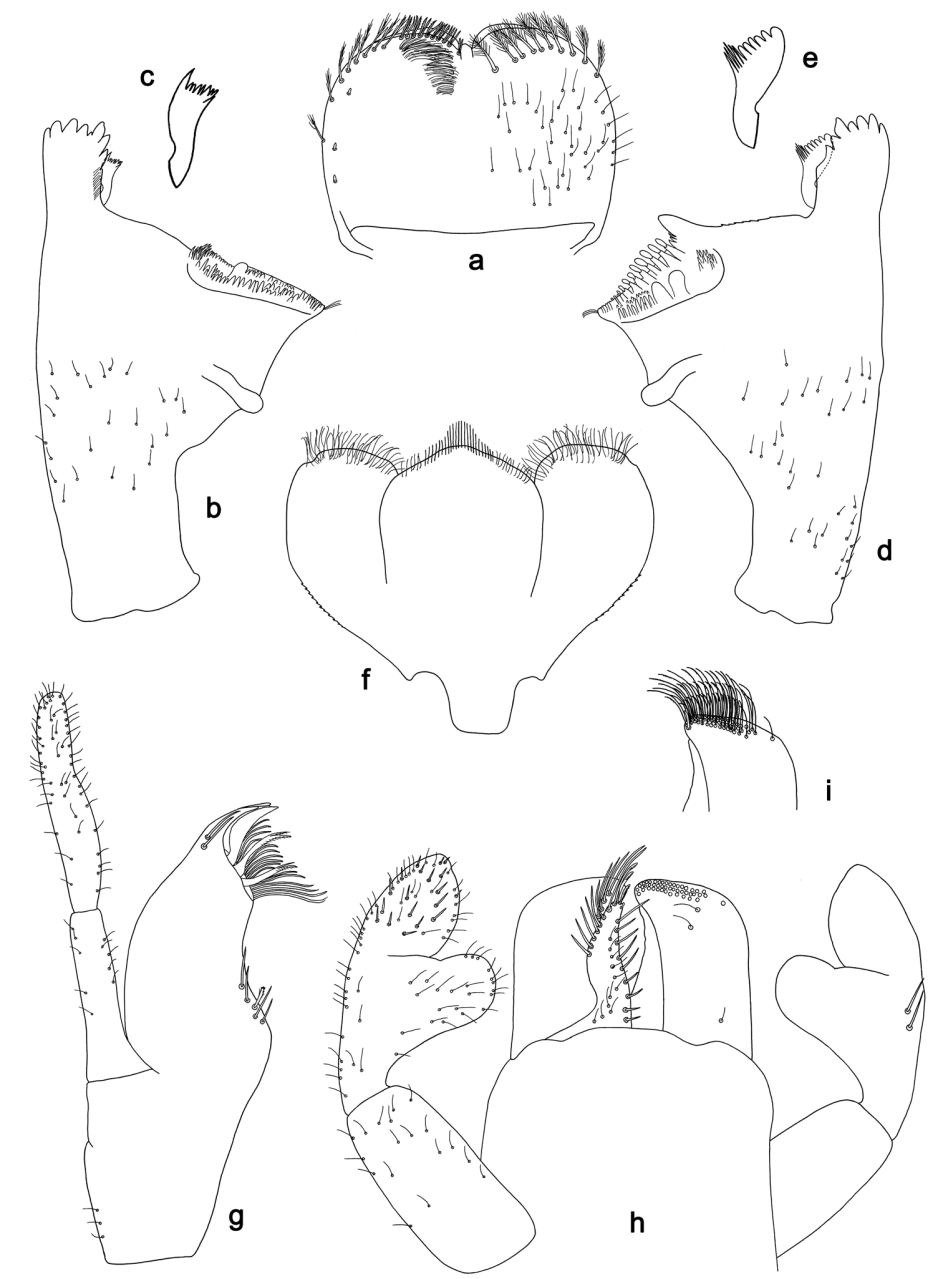
*Labiobaetis paraoperosus* sp. nov., larva morphology: **a** Labrum **b** Right mandible **c** Right prostheca **d** Left mandible **e** Left prostheca **f**Hypopharynx**g** Maxilla **h** Labium **i** Apex of paraglossa.

**Figure 37.**
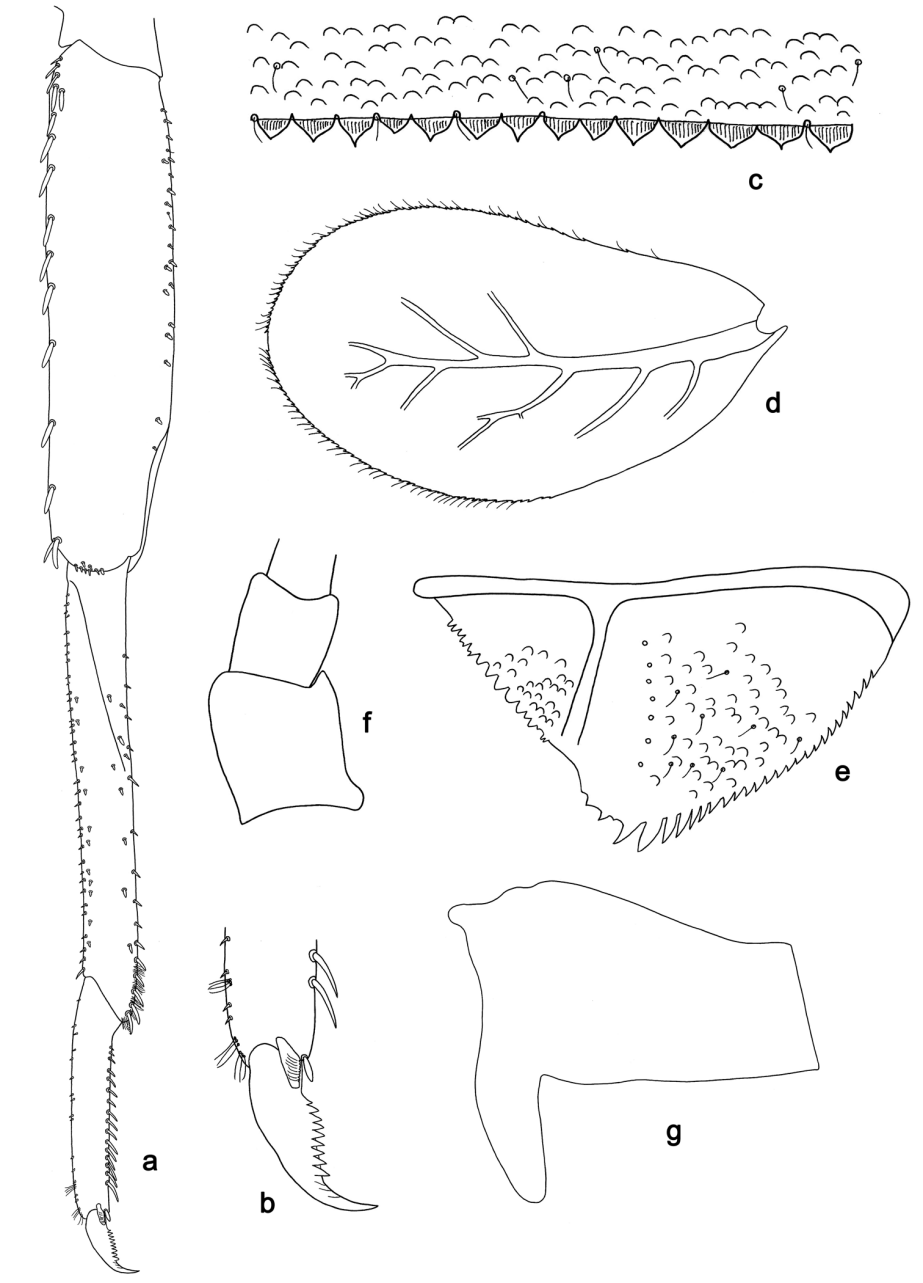
*Labiobaetis paraoperosus* sp. nov., larva morphology: **a** Foreleg **b** Fore claw **c** Tergum IV **d** Gill IV **e** Paraproct **f** Scape **g** Metanotum.

Figures 36 , 37 , 51d , 55b

##### Diagnosis.

**Larva.** Following combination of characters: A) dorsal surface of labrum with submarginal arc of 1 + 9 or 10 long, feathered setae; B) labial palp segment II with a large, thumb-like distomedial protuberance, segment III oblong; C) fore femur rather broad, length ca. 4× maximum width, dorsal margin with a row of 11–18 spine-like setae; D) hindwing pad well developed; E) paraproct distally not expanded, with ca. 30 stout marginal spines.

##### Description.

**Larva** (Figs 36, 37, 51d). Body length 5.5–6.0 mm; antenna approximately 3× as long as head length; cerci ca. 0.7× body length.

*Colouration*. Head, thorax, and abdomen dorsally brown, head and thorax with bright median, dorsal suture, thorax and abdomen with pattern as in Fig. 51d. Forewing pads light brown at base and with light brown distal spots. Head, thorax, and abdomen ventrally very light brown, abdominal segments VIII and IX brown. Legs transparent, femur with a distomedial, brown spot and brown apex, caudal filaments transparent with a dark brown band at ca. 1/3 of cerci.

*Antenna* with scape and pedicel subcylindrical, with well-developed distolateral process at scape; flagellum with lanceolate spines and fine, simple setae on apex of each segment.

*Labrum* (Fig. 36a). Rectangular, length 0.7× maximum width. Distal margin with medial emargination and a small process. Dorsally with medium, fine, simple setae; submarginal arc of setae composed of 1 + 9 or10 long, feathered setae. Ventrally with marginal row of setae composed of lateral and anterolateral long, feathered setae and medial long, bifid, pectinate setae; ventral surface with three short, spine-like setae near lateral and anterolateral margin.

*Right mandible* (Fig. 36b, c). Incisors fused. Outer and inner sets of denticles with 4 + 3 denticles and one minute intermediate denticle. Inner margin of innermost denticle with a row of thin setae. Prostheca robust, apically denticulate. Margin between prostheca and mola straight. Tuft of setae at apex of mola present.

*Left mandible* (Fig. 36d, e). Incisors fused. Outer and inner sets of denticles with 4 + 3 denticles. Prostheca robust, apically with small denticles and comb-shaped structure. Margin between prostheca and mola straight, with minute denticles towards subtriangular process. Subtriangular process long and slender, above level of area between prostheca and mola. Denticles of mola apically constricted. Tuft of setae at apex of mola present.

Both mandibles with lateral margins almost straight. Basal half with fine, simple setae scattered over dorsal surface.

*Hypopharynx* (Fig. 36f). Lingua approx. as long as superlingua. Lingua broader than long; medial tuft of stout setae poorly developed; distal half not expanded. Superlingua rounded; lateral margin rounded; fine, long, simple setae along distal margin.

*Maxilla* (Fig. 36g). Galea-lacinia with two simple, robust apical setae under crown. Inner dorsal row of setae with three denti-setae, distal denti-seta tooth-like, middle and proximal denti-setae slender, bifid and pectinate. Medially with one bipectinate, spine-like seta and 4–5 long, simple setae. Maxillary palp 1.4× as long as length of galea-lacinia; two segmented; palp segment II 1.3× length of segment I; setae on maxillary palp fine, simple, scattered over surface of segments I and II; apex of last segment rounded, with slight excavation at inner distolateral margin.

*Labium* (Fig. 36h, i). Glossa basally broad, narrowing toward apex; shorter than paraglossa; inner margin with eight or nine spine-like setae increasing in length distally; apex with two long and one medium, robust, pectinate setae and one short, robust seta; outer margin with 5–7 spine-like setae increasing in length distally; ventral surface with short, fine, simple, scattered setae. Paraglossa sub-rectangular, curved inward; apex rounded; with three rows of long, robust, distally pectinate setae in apical area and two or three medium, simple setae in anteromedial area and partly one short, simple seta in proximomedial area; dorsally with a row of four or five long, spine-like setae near inner margin. Labial palp with segment I 0.8× length of segments II and III combined. Segment I ventrally with short, fine, simple setae. Segment II with broad thumb-like distomedial protuberance; distomedial protuberance 0.9× width of base at segment III; inner and outer margin with short, fine, simple setae; dorsally with two long, spine-like, simple setae near outer margin. Segment III oblong; apex rounded; length 1.5× width; ventrally covered with short, spine-like, simple setae and short, fine, simple setae.

*Hind wing pads* well developed.

*Foreleg* (Fig. 37a, b). Ratio of foreleg segments 1.2:1.0:0.5:0.2. *Femur*. Length ca. 4× maximum width. Dorsal margin with a row of 11–18 spine-like setae and one or two spine-like setae near margin; length of setae 0.25× maximum width of femur. Apex rounded; with one pair of curved, spine-like setae and some short, stout setae. Many short, stout, lanceolate setae scattered along the ventral margin; femoral patch absent. *Tibia*. Dorsal margin with a row of short, stout setae; on apex one longer seta, and a row of short, stout setae close to dorsal margin. Ventral margin with a row of curved, spine-like setae, distally longer, on apex one bipectinate, spine-like seta and a tuft of long, fine, simple setae. Anterior surface scattered with stout, lanceolate setae. Patellotibial suture present on basal 1/2. *Tarsus*. Dorsal margin with a row of short, stout setae and long, fine, simple setae. Ventral margin with a row of curved, spine-like setae. Tarsal claw with one row of 9–11 denticles; distally pointed; with 3–5 stripes; subapical setae absent.

*Tergum* (Fig. 37c). Surface with irregular rows of U-shaped scale bases and scattered fine, simple setae. Posterior margin of tergum IV with triangular spines, wider than long.

*Gills* (Fig. 37d). Present on segments I–VII. Margin with small denticles intercalating fine simple setae. Tracheae extending from main trunk to inner and outer margins. Gill I ca. 2/3 length of segment II. Gill IV as long as length of segments V and 1/3 VI combined. Gill VII as long as length of segment VIII.

*Paraproct* (Fig. 37e). Distally not expanded with 29–32 stout marginal spines. Surface scattered with U-shaped scale bases and fine, simple setae. Cercotractor with numerous small marginal spines.

##### Etymology.

Refers to the morphological similarity with *L. operosus*.

##### Distribution.

Indonesia: Sumatra.

##### Biological aspects.

The specimens were collected at altitudes between sea level and 890 m, one of them in a small (2 m wide), shallow (25 cm deep) and slow (velocity 0.3 m/s) stream in a highly disturbed area (agriculture, livestock, waste). The substrate was predominantly stone and gravel without patches of leaf litter or dead wood.

##### Type-material.

**Holotype.** Larva (on slide, GBIFCH 00592254), Indonesia, Sumatra, Aceh, Ketambe, Alas River at Cinta Alam Lodge, 03°40.73'N, 97°39.37'E, 400 m, 14.X.2013, M. Balke leg. (SUM 45). Temporary deposited in MZL before definitely housed in MZB. **Paratypes.** 11 larvae (1 on slide, GBIFCH 00592253, 5 in alcohol, GBIFCH 00515329, GBIFCH 00515338, deposited in MZL; 5 in alcohol, GBIFCH 00515328, deposited in ZSM), same data as holotype; 1 larva (on slide, GBIFCH 00592249, deposited in MZL), Indonesia, Sumatra Barat, Sawahlunto, stream, 275 m, 10.XI.2011, 00°41.33'S, 100°46.72'E, M. Balke leg. (UN5); 1 larva (on slide, GBIFCH 00422165, deposited in MZL), Indonesia, Sumatra, volcano Marapi, 00°21.97'S, 100°33.30'E, 890 m, 02.IV.2014, M. Gueuning leg.; 2 larvae (on slides, GBIFCH 00592248, GBIFCH 00592247, deposited in MZL), Indonesia, Sumatra Barat, Tarusan, upstream Tarusan, 01°13.62'S, 100°29.83E, 10 m, 24.V.2010, J.-M. Elouard leg.

### *Labiobaetis seramensis* group of species

With the following combination of characters: A) dorsal surface of labrum with submarginal arc of simple setae; B) labial palp segment II with slender or rather slender, thumb-like distomedial protuberance, segment III approx. semi-circular; C) maxillary palp approx. as long as galea-lacinia; D) six pairs of gills; E) hindwing pads absent; F) distolateral process at scape absent.

#### 22. Labiobaetis seramensis sp. nov.

Taxon classificationAnimaliaEphemeropteraBaetidae

365BB213-E238-5B4E-8728-29F13EE2D93D

http://zoobank.org/6FB7DFAC-A5A8-43DC-88C2-5004D09857E5

**Figure 38.**
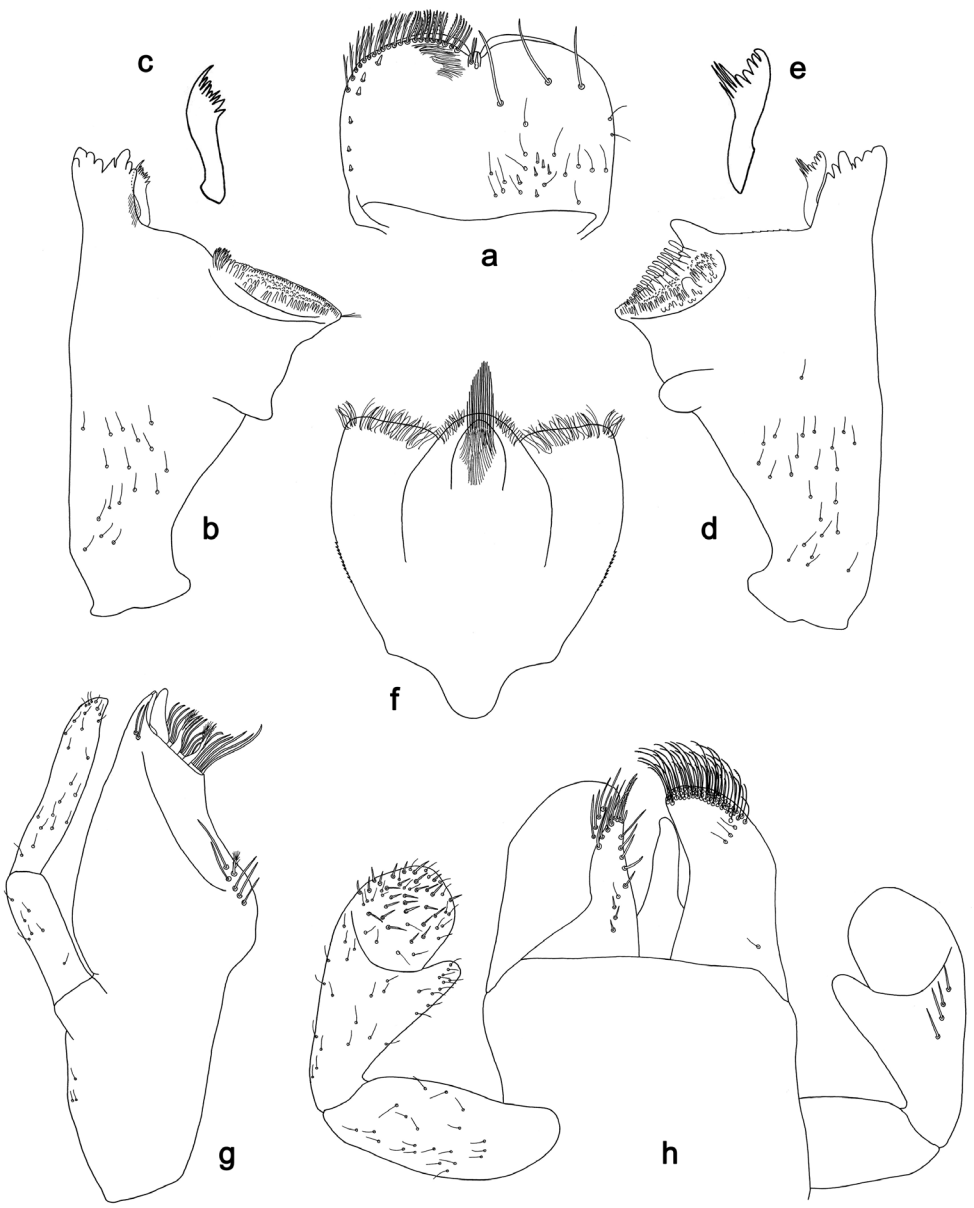
*Labiobaetis seramensis* sp. nov., larva morphology: **a** Labrum **b** Right mandible **c** Right prostheca **d** Left mandible **e** Left prostheca **f**Hypopharynx**g** Maxilla **h** Labium.

**Figure 39.**
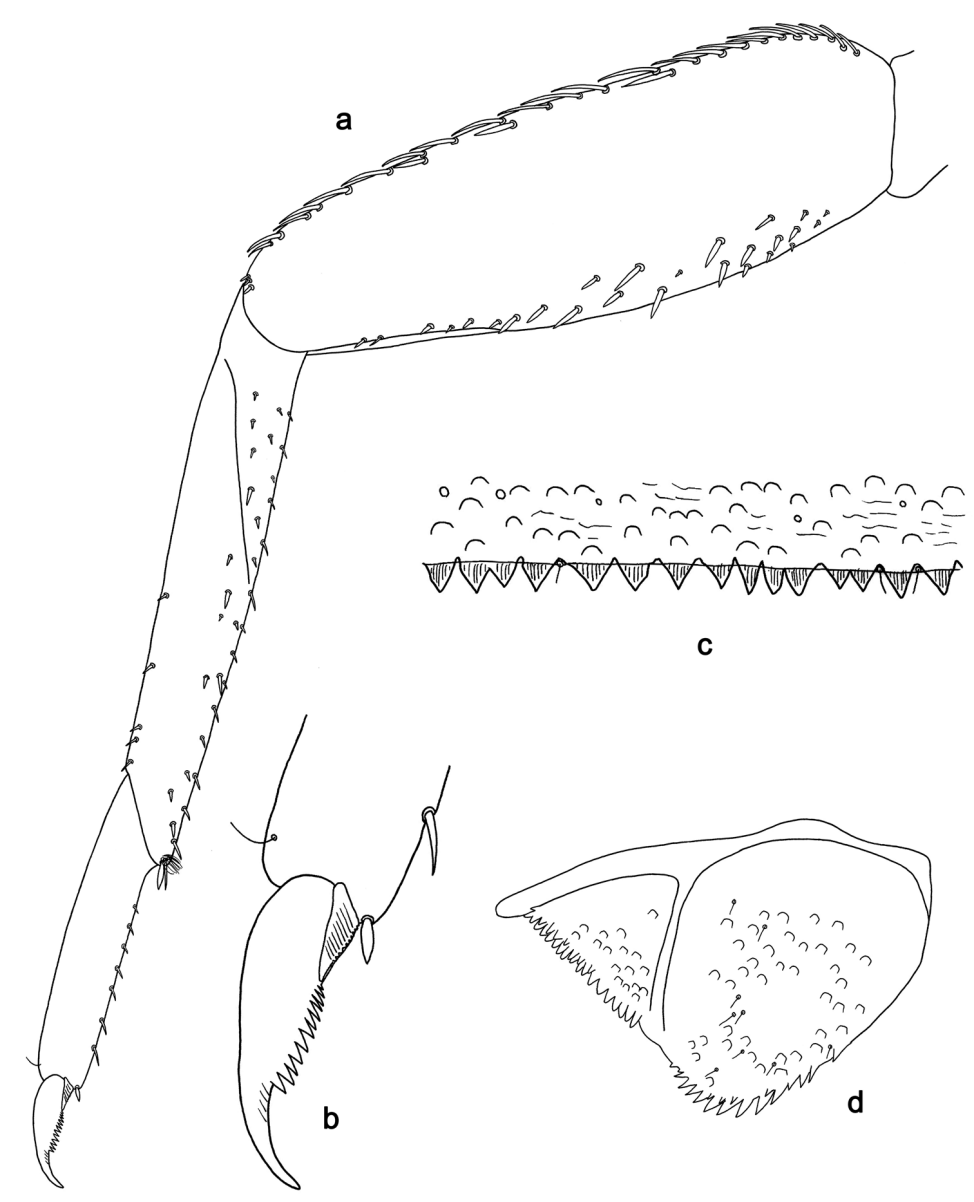
*Labiobaetis seramensis* sp. nov., larva morphology: **a** Foreleg **b** Fore claw **c** Tergum IV **d** Paraproct.

Figures 38 , 39 , 50c , 54a

##### Diagnosis.

**Larva.** Following combination of characters: A) dorsal surface of labrum with submarginal arc of 1 + 2 long, simple setae; B) labial palp segment II with slender, thumb-like distomedial protuberance; C) maxillary palp approx. as long as length of galea-lacinia; D) fore femur rather broad, length ca. 3× width, dorsally with a row of ca. 21 curved, spine-like setae on margin; E) posterior margin of tergum IV with triangular spines, wider than long; F) paraproct distally not expanded, with ca. 15 stout marginal spines and a few stout submarginal spines.

**Description. Larva** (Figs 38, 39, 50c). Body length 4.8 mm.

*Colouration*. Head, thorax, and abdomen dorsally brown, head and thorax with bright median, dorsal suture, thorax with bright pattern as in Fig. 50c, forewing pads with darker striation, abdominal segment VI with bright areas as in Fig. 50c, abdominal segments IX and X light brown. Head, thorax, and abdomen ventrally light brown, legs transparent, femur with brown dorsal margin, caudal filaments transparent.

*Antenna* with scape and pedicel subcylindrical, without distolateral process at scape; flagellum with broad, lanceolate spines and fine, simple setae on apex of each segment.

*Labrum* (Fig. 38a). Rectangular, length 0.7× maximum width. Distal margin with medial emargination and a small process. Dorsally with medium, fine, simple setae and a few short, slightly lanceolate setae; submarginal arc of setae composed of 1 + 2 long, simple setae. Ventrally with marginal row of setae composed of anterolateral long, feathered setae and medial long, bifid, pectinate setae; ventral surface with six short, spine-like setae near lateral and anterolateral margin.

*Right mandible* (Fig. 38b, c). Incisors fused. Outer and inner sets of denticles with 4 + 4 denticles. Inner margin of innermost denticle with a row of thin setae. Prostheca robust, apically denticulate. Margin between prostheca and mola slightly convex. Tuft of setae at apex of mola present.

*Left mandible* (Fig. 38d, e). Incisors fused. Outer and inner sets of denticles with 3 + 3 denticles and one minute intermediate denticle. Prostheca robust, apically with small denticles and comb-shaped structure. Margin between prostheca and mola straight, with minute denticles towards subtriangular process. Subtriangular process long and slender, above level of area between prostheca and mola. Denticles of mola apically constricted. Tuft of setae at apex of mola present.

Both mandibles with lateral margins almost straight. Basal half with fine, simple setae scattered over dorsal surface.

*Hypopharynx* (Fig. 38f). Lingua approx. as long as superlingua. Lingua approx. as broad as long; medial tuft of stout setae well developed, long; distal half not expanded. Superlingua straight; lateral margin rounded; fine, long, simple setae along distal margin.

*Maxilla* (Fig. 38g). Galea-lacinia with two simple, robust apical setae under crown. Inner dorsal row of setae with three denti-setae, distal denti-seta tooth-like, middle and proximal denti-setae slender, bifid and pectinate. Medially with one bipectinate, spine-like seta and five long, simple setae. Maxillary palp approx. as long as length of galea-lacinia; two segmented; palp segment II 1.5× length of segment I; setae on maxillary palp fine, simple, scattered over surface of segments I and II; apex of last segment slightly pointed, with slight excavation at inner distolateral margin.

*Labium* (Fig. 38h). Glossa basally broad, narrowing toward apex; shorter than paraglossa; inner margin with five spine-like setae increasing in length distally; apex with two long and one medium, robust, pectinate setae and one short, robust seta; outer margin with four long, spine-like setae; ventral surface with short, fine, simple and short, spine-like setae. Paraglossa sub-rectangular, curved inward; apex rounded; with three rows of long, robust, distally pectinate setae in apical area and three medium, simple setae in anteromedial area and one fine, simple seta in proximomedial area; dorsally with a row of four long, spine-like setae near inner margin. Labial palp with segment I 0.8× length of segments II and III combined. Segment I ventrally with short, fine, simple setae. Segment II with slender, thumb-like distomedial protuberance; distomedial protuberance 0.4× width of base of segment III; inner and outer margin with short, fine, simple setae; dorsally with a row of four medium, spine-like setae near outer margin. Segment III approximately semi-circular; apex rounded; length 1.1× width; ventrally covered with short, spine-like, simple setae and short, fine, simple setae.

*Hind wing pads* absent.

*Foreleg* (Fig. 39a, b). Ratio of foreleg segments 1.3:1.0:0.6:0.2. *Femur*. Length ca. 3× maximum width. Dorsal margin with a row of ca. 21 curved, spine-like setae and a few spine-like setae near margin; length of setae 0.25× maximum width of femur. Apex rounded; with a few short, stout, pointed setae. Many short to long, lanceolate setae scattered along the ventral margin; femoral patch absent. *Tibia.* Dorsal margin with a row of short, stout setae. Ventral margin with a row of curved, spine-like setae, on apex one to several stout, spine-like setae and a tuft of long, fine, simple setae. Anterior surface scattered with stout, lanceolate setae. Patellotibial suture present on basal 1/2. *Tarsus.* Dorsal margin almost bare. Ventral margin with a row of curved spine-like setae. Tarsal claw with one row of ten denticles; distally pointed; with four stripes; subapical setae absent.

*Tergum* (Fig. 39c). Surface with irregular rows of U-shaped scale bases and scattered micropores. Posterior margin of tergum IV with triangular spines, approx. as long as wide.

*Gills*. Present on segments II–VII.

*Paraproct* (Fig. 39d). Distally not expanded, with ca. 15 stout marginal spines and some submarginal spines. Surface scattered with U-shaped scale bases and fine, simple setae. Cercotractor with numerous small marginal spines and some submarginal spines.

##### Etymology.

Refers to the island Seram, where the specimens were collected.

##### Distribution.

Indonesia: Seram.

##### Biological aspects.

The specimen was collected at an altitude of 2,000 m.

##### Type-material.

**Holotype.** Larva (on slide, GBIFCH 00592207), Indonesia, Maluku, Seram, Waihuhu below Mt. Binaya, 2,000 m, 10.IV.2012, 03°10.01'S, 129°28.93'E, M. Balke leg. (AMB11). Temporary deposited in MZL before definitely housed in MZB.

#### 23. Labiobaetis wahai sp. nov.

Taxon classificationAnimaliaEphemeropteraBaetidae

B421B16C-3964-5AEF-8AA4-0C2B01A22C45

http://zoobank.org/F4E7E5BA-C06A-455A-ACBB-B1D446F08A67

**Figure 40.**
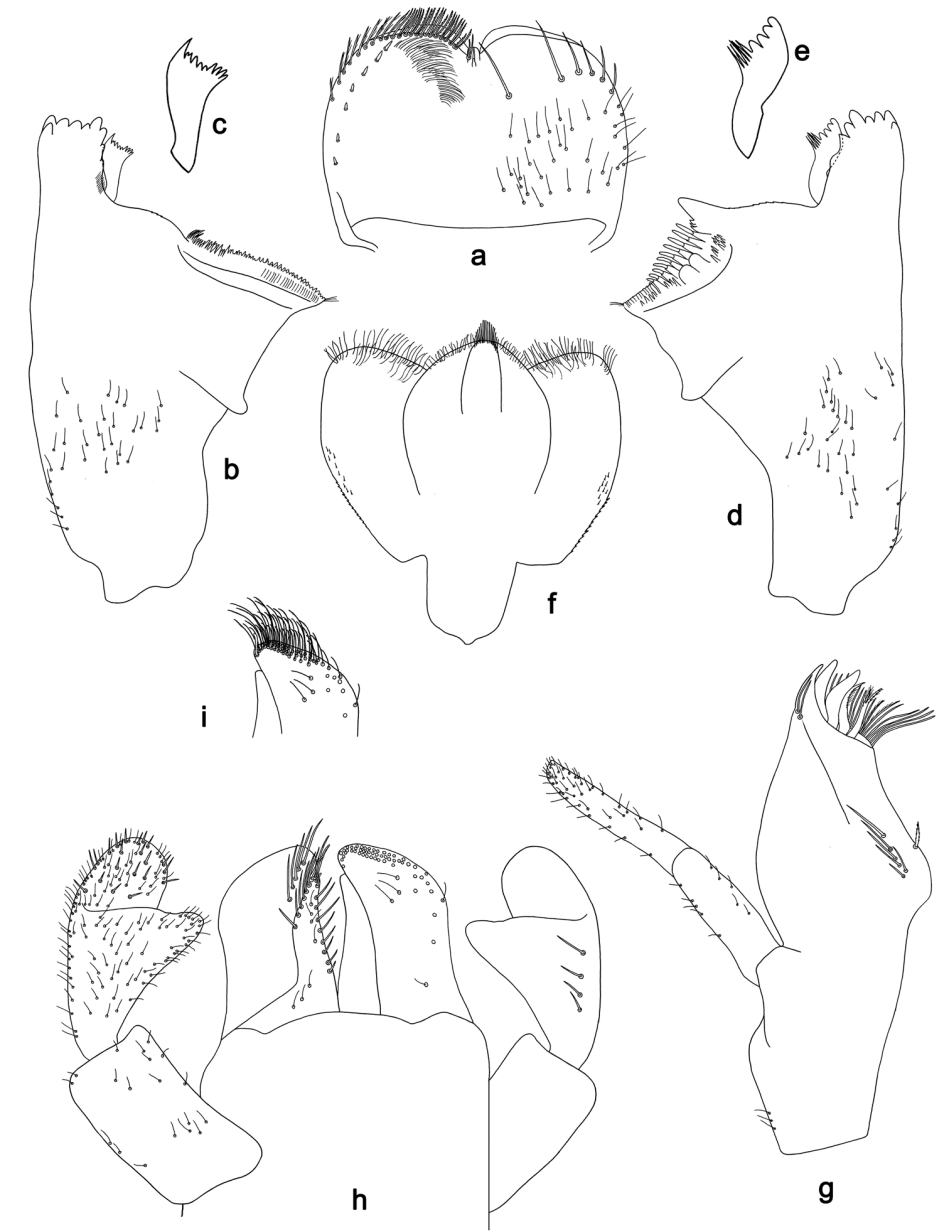
*Labiobaetis wahai* sp. nov., larva morphology: **a** Labrum **b** Right mandible **c** Right prostheca **d** Left mandible **e** Left prostheca **f**Hypopharynx**g** Maxilla **h** Labium **i** Apex of paraglossa.

**Figure 41.**
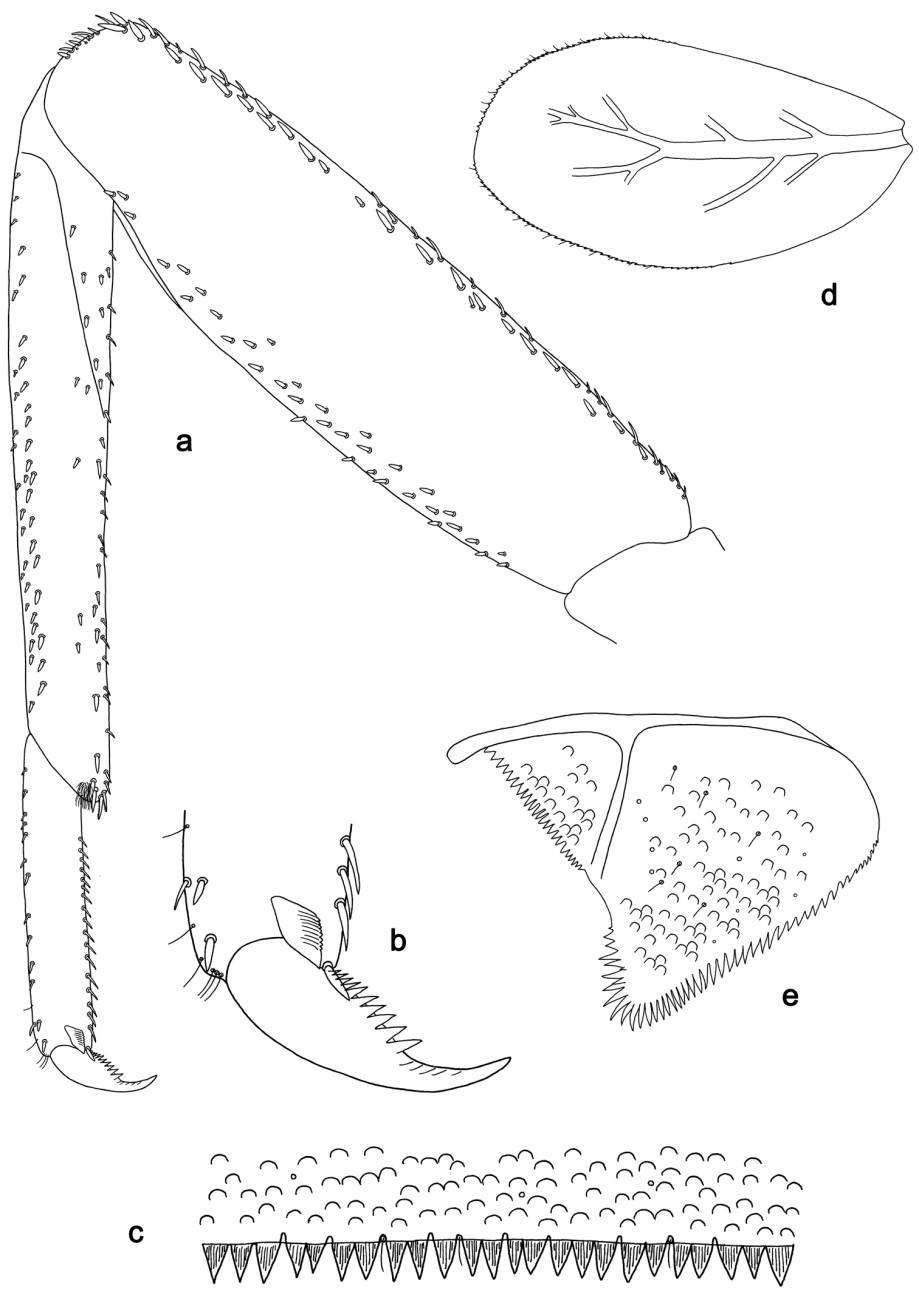
*Labiobaetis wahai* sp. nov., larva morphology: **a** Foreleg **b** Fore claw **c** Tergum IV **d** Gill IV **e** Paraproct.

Figures 40 , 41 , 50d , 54b

##### Diagnosis.

**Larva.** Following combination of characters: A) dorsal surface of labrum with submarginal arc of 1 + 5 long, simple setae; B) labial palp segment II with thumb-like distomedial protuberance; C) maxillary palp approx. as long as length of galea-lacinia; D) fore femur rather slender, length 3.6 × maximum width, dorsal margin with a row of ca. 22 curved, spine-like setae, an additional row of spine-like setae along margin; E) posterior margin of tergum IV with triangular spines, longer than wide; F) paraproct distally expanded, with ca. 47 stout marginal spines.

##### Description.

**Larva** (Figs 40, 41, 50d). Body length 5.5 mm.

*Colouration*. Head, thorax, and abdomen dorsally brown, head and thorax with bright median, dorsal suture. Head, thorax, and abdomen ventrally light brown, legs light brown with distomedial, brown streak on femur, caudal filaments light brown.

*Antenna* with scape and pedicel subcylindrical, without distolateral process at scape; flagellum with broad, lanceolate spines and fine, simple setae on apex of each segment.

*Labrum* (Fig. 40a). Rectangular, length 0.7× maximum width. Distal margin with medial emargination and a small process. Dorsally with medium, fine, simple setae scattered over surface; submarginal arc of setae composed of 1 + 5 long, simple setae. Ventrally with marginal row of setae composed of lateral and anterolateral long, feathered setae and medial long, bifid, pectinate setae; ventral surface with seven short, spine-like setae near lateral and anterolateral margin.

*Right mandible* (Fig. 40b, c). Incisors fused. Outer and inner sets of denticles with 4 + 4 denticles and one small intermediate denticle. Inner margin of innermost denticle with a row of thin setae. Prostheca robust, apically denticulate. Margin between prostheca and mola slightly convex, with minute denticles. Tuft of setae at apex of mola present.

*Left mandible* (Fig. 40d, e). Incisors fused. Outer and inner sets of denticles with 4 + 3 denticles. Prostheca robust, apically with small denticles and comb-shaped structure. Margin between prostheca and mola slightly convex, with minute denticles toward subtriangular process. Subtriangular process long and slender, above level of area between prostheca and mola. Denticles of mola apically constricted. Tuft of setae at apex of mola present.

Both mandibles with lateral margins almost straight. Basal half with fine, simple setae scattered over dorsal surface.

*Hypopharynx* (Fig. 40f). Lingua approx. as long as superlingua. Lingua approx. as broad as long; medial tuft of stout setae well developed, short; distal half not expanded. Superlingua rounded; lateral margin rounded; fine, long, simple setae along distal margin.

*Maxilla* (Fig. 40g). Galea-lacinia with two simple, robust apical setae under crown. Inner dorsal row of setae with three denti-setae, distal denti-seta tooth-like, middle and proximal denti-setae slender, bifid and pectinate. Medially with one bipectinate, spine-like seta and six long, simple setae. Maxillary palp approx. as long as length of galea-lacinia; two segmented; palp segment II 1.2× length of segment I; setae on maxillary palp fine, simple, scattered over surface of segments I and II; apex of last segment slightly pointed, without excavation at inner distolateral margin.

*Labium* (Fig. 40h, i). Glossa basally broad, narrowing toward apex; shorter than paraglossa; inner margin with eight spine-like setae increasing in length distally; apex with two long and one medium, robust, pectinate setae and one short, robust seta; outer margin with 6–8 spine-like setae; ventral surface with fine, simple, scattered setae. Paraglossa sub-rectangular, curved inward; apex rounded; with three rows of long, robust, distally pectinate setae in apical area and three medium, simple setae in anteromedial area and one fine, simple seta in proximomedial area; dorsally with a row of five long, spine-like setae near inner margin. Labial palp with segment I 0.8× length of segments II and III combined. Segment I ventrally with short and medium, fine, simple setae. Segment II with thumb-like distomedial protuberance; distomedial protuberance 0.6× width of base of segment III; inner and outer margin with short, fine, simple setae; dorsally with a row of four medium, spine-like setae near outer margin. Segment III approx. semi-circular; apex rounded; length 0.9× width; ventrally covered with short, spine-like, simple setae and short, fine, simple setae.

*Hind wing pads* absent.

*Foreleg* (Fig. 41a, b). Ratio of foreleg segments 1.1:1.0:0.4:0.2. *Femur*. Length ca. 4× maximum width. Dorsal margin with Dorsal margin with a row of ca. 22 curved, spine-like setae and a row of spine-like setae along margin; length of setae 0.11× maximum width of femur. Apex rounded; with one pair of spine-like setae and some shorter, stout setae. Many stout, lanceolate setae scattered along the ventral margin; femoral patch absent. *Tibia*. Dorsal margin with a row of stout, lanceolate setae and many stout, lanceolate setae scattered along the margin. Ventral margin with a row of curved, spine-like setae, on apex some stout, spine-like, partly bipectinate setae and a tuft of fine, simple setae. Anterior surface scattered with stout, lanceolate setae. Patellotibial suture present on basal 1/3. *Tarsus*. Dorsal margin with a row of short, spine-like setae. Ventral margin with a row of curved, spine-like setae. Tarsal claw with one row of ten denticles; distally pointed; with five stripes; subapical setae absent.

*Tergum* (Fig. 41c). Surface with irregular rows of U-shaped scale bases and scattered micropores. Posterior margin of tergum IV with triangular spines, longer than wide.

*Gills* (Fig. 41d). Present on segments II–VII. Margin with small denticles intercalating fine simple setae. Tracheae extending from main trunk to inner and outer margins. Gill VII as long as length of segments VIII and 1/3 IX combined.

*Paraproct* (Fig. 41e). Distally expanded, with > 40 stout marginal spines. Surface scattered with U-shaped scale bases, fine, simple setae and micropores. Cercotractor with numerous small marginal spines.

##### Etymology.

Dedicated to the indigenous Wahai people from Seram.

##### Distribution.

Indonesia: Seram.

##### Biological aspects.

The specimens were collected at an altitude of 600 m.

##### Type-material.

**Holotype.** Larva (on slide, GBIFCH 00592208), Indonesia, Maluku, Seram, Kanikeh, 607 m, 07.IV.2012, 03°06.52'S, 129°28.80'E, M. Balke leg. (AMB07). Temporary deposited in MZL before definitely housed in MZB. **Paratype.** Larva (on slide, GBIFCH 00592209, deposited in MZL), same data as holotype.

### Not assigned to a group

#### 24. Labiobaetis borneoensis

Taxon classificationAnimaliaEphemeropteraBaetidae

(Müller-Liebenau, 1984)

21CFCFE9-18F8-576D-AF5F-F6BA3FA739C5

Figures 42 , 53b

##### Diagnosis.

**Larva.** Following combination of characters: A) dorsal surface of labrum with submarginal arc of nine or ten feathered setae; B) labial palp segment II with a large, lobed distomedial protuberance, segment III oblong, apically slightly pointed; C) fore femur rather slender, length 3.6× maximum width, dorsal margin with a row of 11–13 curved, spine-like setae; D) seven pairs of gills; E) hindwing pads present, small; F) distolateral process at scape well developed.

##### Examined material.

1 larva (on slide, GBIFCH00465236), Indonesia, East Kalimantan, Bas. Malinau, River Rian, loc. Langap South (1997-bloc 6), trib. Belakau, 03°04.07'N, 116°30.43'E, 07.VII.2000, P. Derleth leg.; 1 larva (on slide, GBIFCH00465237), Indonesia, East Kalimantan, Bas. Malinau, River Rian, loc. Seturan (1998-block 32-33), tributary, 03°00.95'N, 116°32.27'E, 30.III.2001, P. Derleth leg.

**Figure 42.**
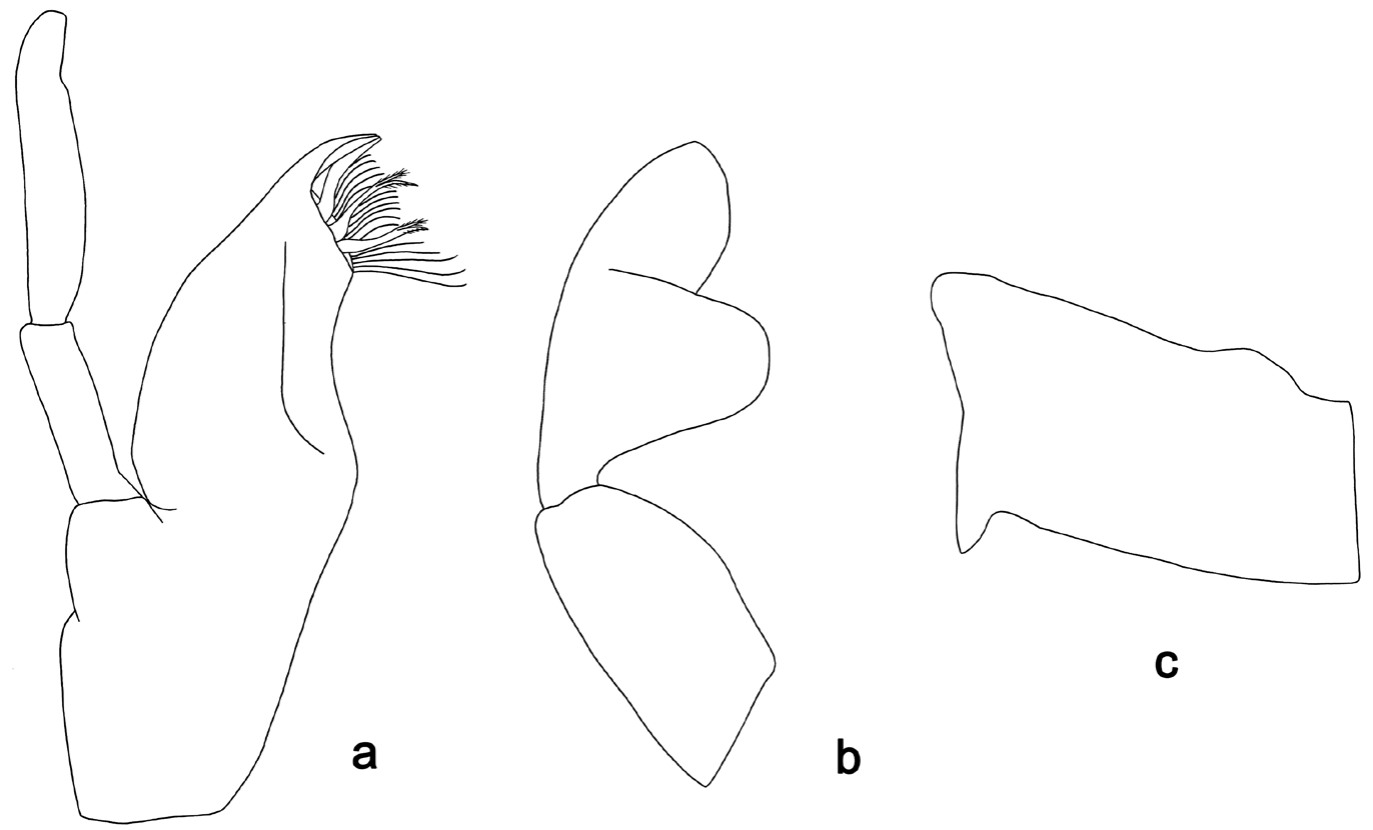
*Labiobaetis borneoensis*, larva morphology: **a** Maxilla **b** Labial palp **c** Metanotum.

#### 25. Labiobaetis moriharai

Taxon classificationAnimaliaEphemeropteraBaetidae

(Müller-Liebenau, 1984)

C1DB481D-0E93-5CAD-BEFF-512DD42D6190

Figures 43 , 53b

##### Diagnosis.

**Larva.** Following combination of characters: A) dorsal surface of labrum with submarginal arc of 1 + 8–10 simple setae, the first three after the central seta are longer than the others and decreasing in length; B) labial palp segment II with a large, lobed distomedial protuberance, segment III conical, apically slightly truncate; C) fore femur rather broad, length 3.4× maximum width, dorsal margin with a row of ca. ten curved, spine-like setae; D) six pairs of gills; E) hindwing pads present, minute; F) distolateral process at scape well developed; G) paraproct distally not expanded, with ca. 12 stout marginal spines.

##### Examined material.

**Paratype.** 1 larva (on slide, no. 41), W. Malaysia, Trib. of Gombak River, 16 ½ miles N of Kuala Lumpur, 14.XI.[19]68, Bishop leg.

**Figure 43.**
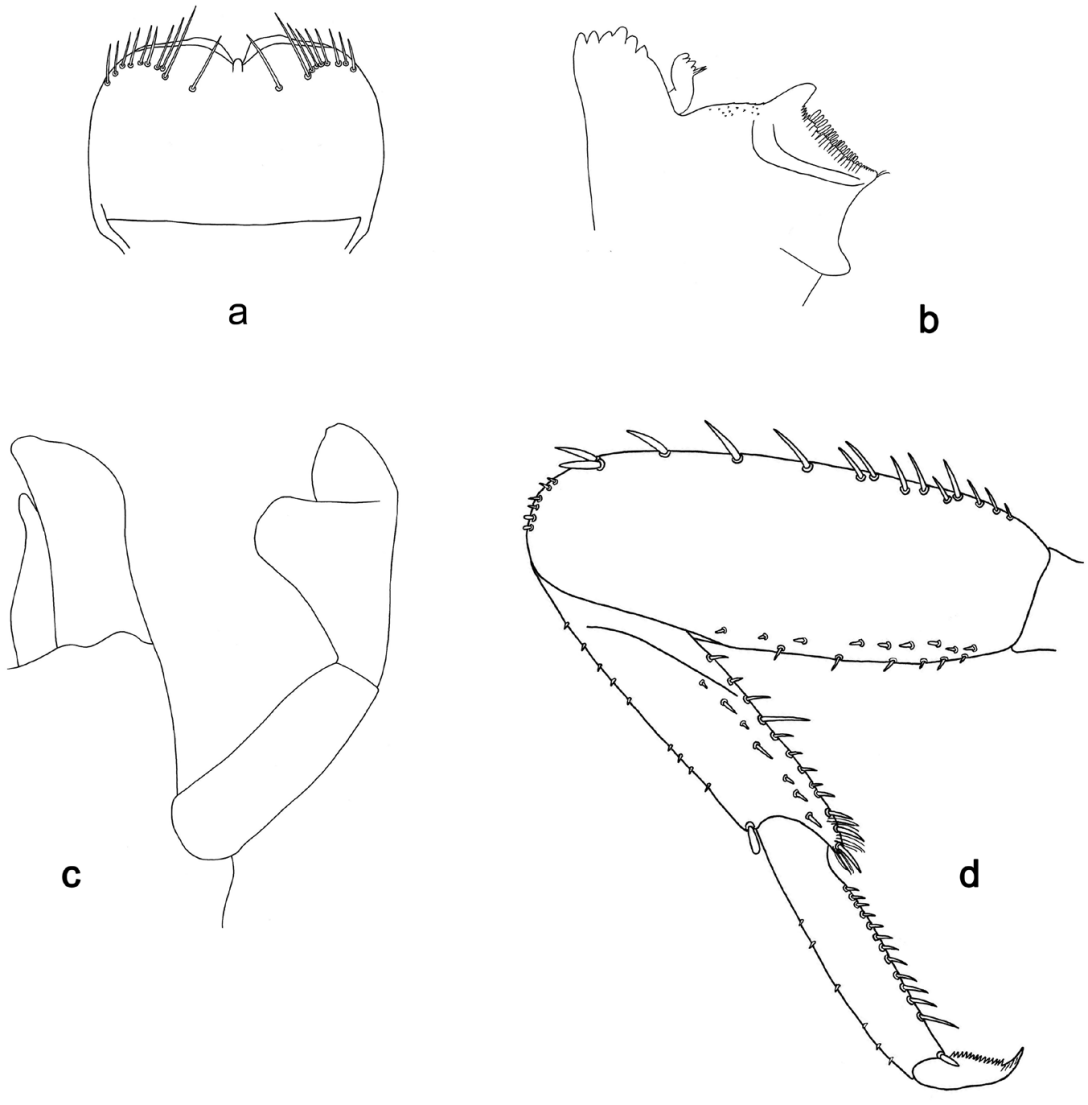
*Labiobaetis moriharai*, larva morphology: **a** Labrum **b** Left mandible **c** Labium **d** Foreleg.

#### 26. Labiobaetis multus

Taxon classificationAnimaliaEphemeropteraBaetidae

(Müller-Liebenau, 1984)

1318C5A7-3FF2-5A57-A23E-4DEE8C81020E

**Figure 44.**
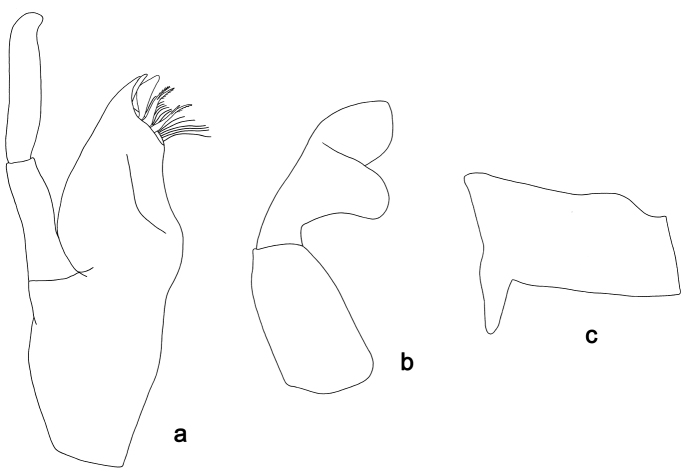
*Labiobaetis multus*, larva morphology: **a** Maxilla **b** Labial palp **c** Metanotum.

Figures 44 , 51a , 53b , 55b

##### Diagnosis.

**Larva.** Following combination of characters: A) dorsal surface of labrum with submarginal arc of 1 + 2–3 simple setae; B) labial palp segment II with a thumb-like distomedial protuberance, segment III slightly pentagonal, apically slightly pointed; C) fore femur rather broad, length ca. 3× maximum width, dorsal margin with a row of 8–10 curved, spine-like setae; D) seven pairs of gills; E) hindwing pads well developed; F) distolateral process at scape well developed; G) paraproct distally not expanded, with ca. 13 stout marginal spines.

##### Examined material.

**Paratypes.** 1 larva (on slide, no. 13), W. Malaysia, Gombak River, 4½ miles N of Kuala Lumpur, 12.XII.[19]68, Bishop leg.; 1 larva (on slide, no. 28), W. Malaysia, Gombak Riv., 9 miles N of Kuala Lumpur, 14.I.[19]68, Bishop leg. **Other material.** 50 larvae (4 on slides, GBIFCH00465238, GBIFCH00465239, GBIFCH00465240, GBIFCH00465241; 46 in alcohol, GBIFCH00515339), Indonesia, Sumatra Barat, Harau Canyon, stream near Ikbal's cottage, 520 m, 23.VI.2012, 00°06.44'S, 100°40.37'E, M. Balke leg. (UN11); 2 larvae (1 on slide, GBIFCH00465242; 1 in alcohol, GBIFCH00235847), Indonesia, Sumatra Barat, Talawi, Ombilin River, 277 m, 08.XI.2011, 00°34.15'S, 100°43.54'E, M. Balke leg. (UN4). Deposited in MZL.

#### 27. Labiobaetis jonasi sp. nov.

Taxon classificationAnimaliaEphemeropteraBaetidae

C3D39B0A-F293-5C9E-A192-96D064D66BF7

http://zoobank.org/32A9DD5B-2B58-44ED-ACED-33DF13509BD4

**Figure 45.**
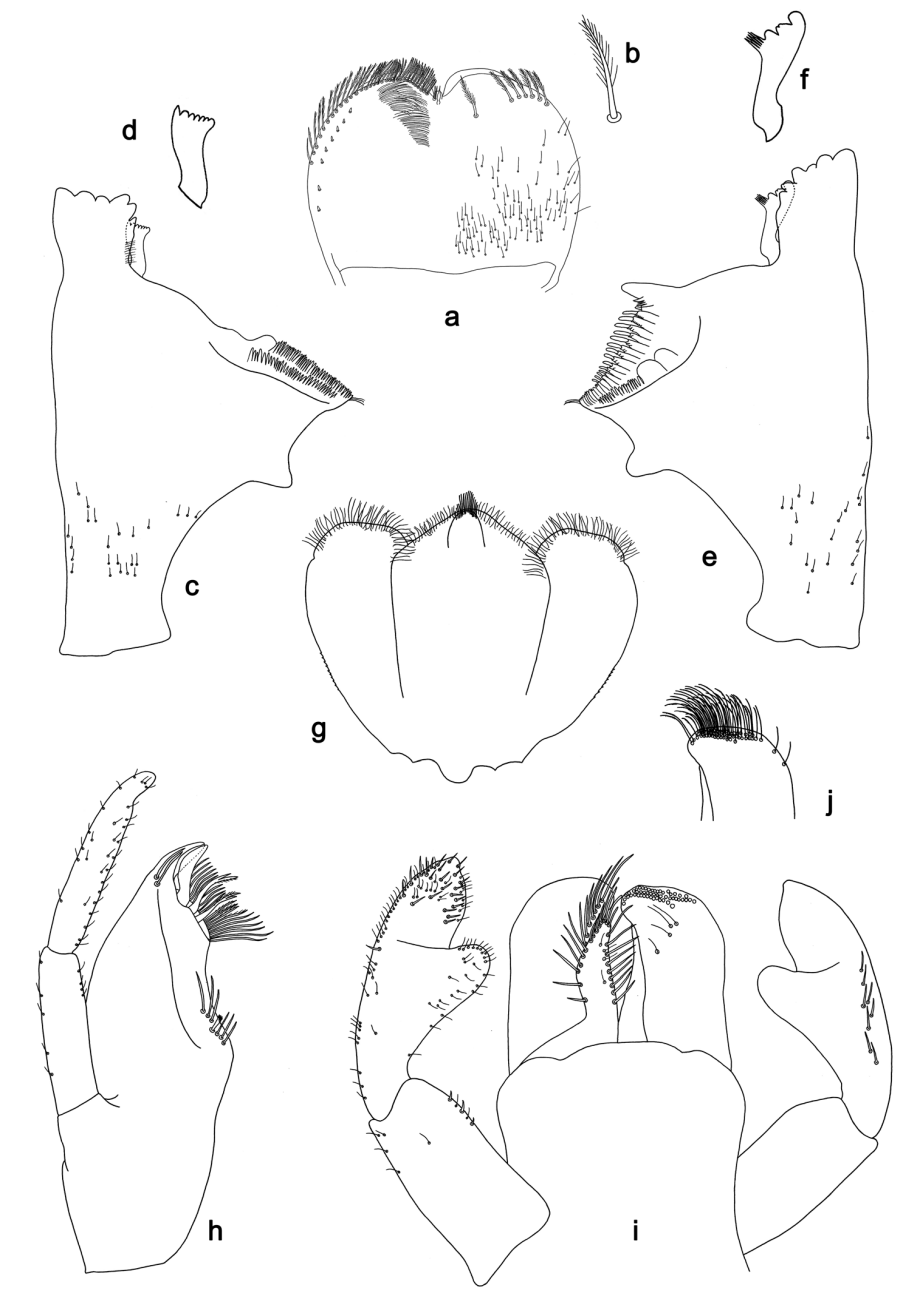
*Labiobaetis jonasi* sp. nov., larva morphology: **a** Labrum **b** Seta of the submarginal arc on the dorsal surface of the labrum **c** Right mandible **d** Right prostheca **e** Left mandible **f** Left prostheca **g**Hypopharynx**h** Maxilla **i** Labium **j** Apex of paraglossa.

**Figure 46.**
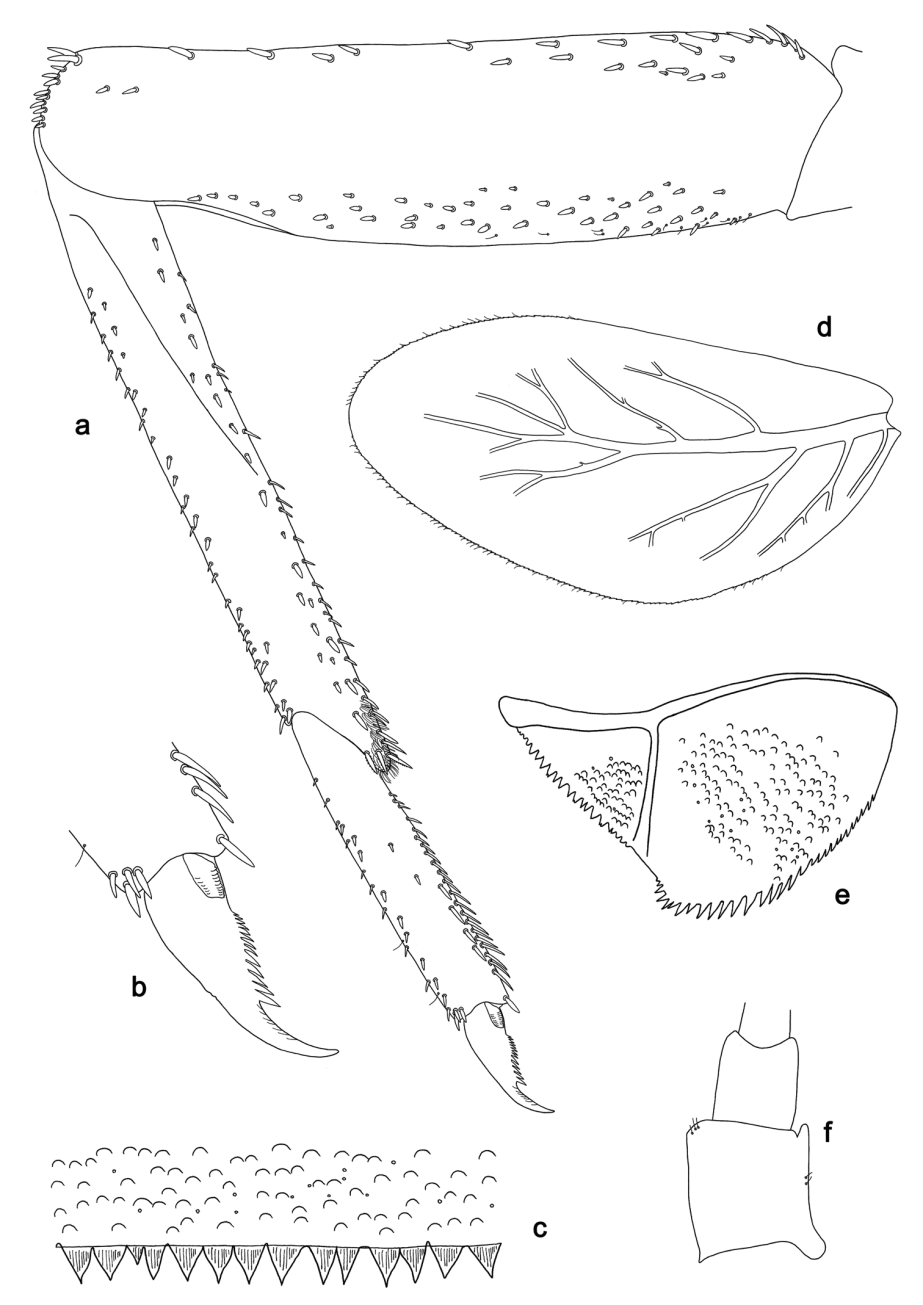
*Labiobaetis jonasi* sp. nov., larva morphology: **a** Foreleg **b** Fore claw **c** Tergum IV **d** Gill IV **e** Paraproct **f** Scape.

Figures 45 , 46 , 51b, c , 52f , 55a

##### Diagnosis.

**Larva.** Following combination of characters: A) dorsal surface of labrum with submarginal arc of 1 + 6 long, feathered setae; B) labial palp segment II with a thumb-like distomedial protuberance, segment III oblong and pointed; C) fore femur rather slender, length ca. 4× maximum width, dorsal margin with a row of ca. 13 lanceolate, spine-like setae and some submarginal setae; D) mouthparts including maxillary palp and labial palp sclerotized, dark brown; E) paraproct distally not expanded, with 30–36 stout marginal spines.

##### Description.

**Larva** (Figs 45, 46, 51b, c, 52f). Body length 6.8–7.6 mm; antenna approximately 3× as long as head length.

*Colouration*. Head, thorax, and abdomen dorsally brown to dark brown, head and thorax with bright median, dorsal suture; thorax and abdomen with bright pattern as in Fig. 51b, c; forewing pads with dark striation and mature larva additionally with bright striation close to margin. Head, thorax, and abdomen ventrally light brown to dark brown, legs with yellowish and brown pattern as in Fig. 52f, mouthparts including maxillary and labial palp brown. Caudal filaments brown with a darker section in the middle part.

*Antenna* with scape and pedicel subcylindrical, with well-developed distolateral process at scape; flagellum with lanceolate spines and fine, simple setae on apex of each segment.

*Labrum* (Fig. 45a, b). Rectangular, length 0.8× maximum width. Distal margin with medial emargination and a small process. Dorsally with medium, fine, simple setae scattered over surface; submarginal arc of setae composed of 1 + 6 long, feathered setae. Ventrally with marginal row of setae composed of lateral and anterolateral long, feathered setae and medial long, bifid, pectinate setae; ventral surface with eight short, spine-like setae near lateral and anterolateral margin.

*Right mandible* (Fig. 45c, d). Incisors fused. Outer and inner sets of denticles with 3 + 3 denticles. Inner margin of innermost denticle with a row of thin setae. Prostheca robust, apically denticulate. Margin between prostheca and mola straight. Tuft of setae at apex of mola present.

*Left mandible* (Fig. 45e, f). Incisors fused. Outer and inner sets of denticles with 3 + 3 denticles. Prostheca robust, apically with small denticles and comb-shaped structure. Margin between prostheca and mola straight. Subtriangular process long and slender, above level of area between prostheca and mola. Denticles of mola apically constricted. Tuft of setae at apex of mola present.

Both mandibles with lateral margins almost straight. Basal half with fine, simple setae scattered over dorsal surface.

*Hypopharynx* (Fig. 45g). Lingua longer than superlingua. Lingua longer than broad; medial tuft of stout setae well developed, short; distal half laterally expanded. Superlingua straight; lateral margin rounded; fine, long, simple setae along distal margin.

*Maxilla* (Fig. 45h). Galea-lacinia with two simple, robust apical setae under crown. Inner dorsal row of setae with three denti-setae, distal denti-seta tooth-like, middle and proximal denti-setae slender, bifid and pectinate. Medially with one bipectinate, spine-like seta and six long, simple setae. Maxillary palp 1.4× as long as length of galea-lacinia; two segmented; palp segment II 1.3× length of segment I; setae on maxillary palp fine, simple, scattered over surface of segments I and II; apex of last segment rounded, with excavation at inner distolateral margin.

*Labium* (Fig. 45i, j). Glossa basally broad, narrowing toward apex; shorter than paraglossa; inner margin with 9–11 spine-like setae increasing in length distally; apex with two long and one medium, robust setae; outer margin with nine or ten long, spine-like setae increasing in length distally; ventral surface with short, fine, simple setae. Paraglossa sub-rectangular, curved inward; apex rounded; with three rows of long, robust, distally pectinate setae in apical area and a row of four medium, simple setae in anteromedial area; dorsally with a row of six or seven long, spine-like setae near inner margin. Labial palp with segment I 0.8× length of segments II and III combined. Segment I ventrally with short, fine, simple setae and some short, stout, simple setae at inner margin. Segment II with thumb-like distomedial protuberance; distomedial protuberance 0.6× width of base of segment III; inner and outer margin with short, fine, simple setae; dorsally with a row of eight medium, spine-like, simple setae near outer margin. Segment III oblong; apex slightly pointed; length 1.3× width; ventrally covered with short, spine-like, simple setae and short, fine, simple setae.

*Hind wing pads* absent.

*Foreleg* (Fig. 46a, b). Ratio of foreleg segments 1.2:1.0:0.5:0.2, or 1.1:1.0:0.4:0.1. *Femur*. Length ca. 4× maximum width. Dorsal margin with a row of ca. 13 spine-like setae and some spine-like setae near margin; length of setae 0.16× maximum width of femur. Apex rounded; with one pair of spine-like setae and some short, stout setae. Many stout, lanceolate setae scattered along the ventral margin; femoral patch reduced to a few setae. *Tibia*. Dorsal margin with a row of stout, lanceolate setae and additional stout, lanceolate setae near margin. Ventral margin with a row of curved, spine-like setae, on apex several spine-like, bipectinate setae and a tuft of fine, simple setae. Anterior surface scattered with stout, lanceolate setae. Patellotibial suture present on basal 1/2. *Tarsus*. Dorsal margin with a row of short, spine-like setae and additional stout, lanceolate setae near margin. Ventral margin with a row of curved, spine-like setae and some spine-like setae near margin. Tarsal claw with one row of 13 denticles; distally pointed; with seven or eight stripes; subapical setae absent.

*Tergum* (Fig. 46c). Surface with irregular rows of U-shaped scale bases and scattered micropores. Posterior margin of tergum IV with triangular spines, longer than wide.

*Gills* (Fig. 46d). Present on segments I–VII. Margin with small denticles intercalating fine simple setae. Tracheae extending from main trunk to inner and outer margins. Gill I as long as length of segment II. Gill IV as long as length of segments V, VI, and 1/4 VII combined. Gill VII as long as length of segments VIII and IX combined.

*Paraproct* (Fig. 46e). Distally not expanded, with 30–36 stout marginal spines. Surface scattered with U-shaped scale bases and micropores. Cercotractor with numerous small marginal spines.

##### Etymology.

Dedicated to Jonas Gattolliat, son of one of the authors (JLG).

##### Distribution.

Indonesia: Sumba.

##### Biological aspects.

The specimens were collected at an altitude of 400 m.

##### Type-material.

**Holotype.** Larva (on slide, GBIFCH 00592231), Indonesia, Sumba, Waikelo stream, 400 m, 27.IX.2011, 09°35.74'S, 119°20.41'E, M. Balke leg. (SUA04). Temporary deposited in MZL before definitely housed in MZB. **Paratypes.** 30 larvae (2 on slides, GBIFCH 00592233, GBIFCH 00592232, 17 in alcohol, GBIFCH 00515352, GBIFCH 00654966, GBIFCH 00654967, deposited in MZL; 11 in alcohol, GBIFCH 00515353, deposited in ZSM), same data as holotype.

**Figure 47.**
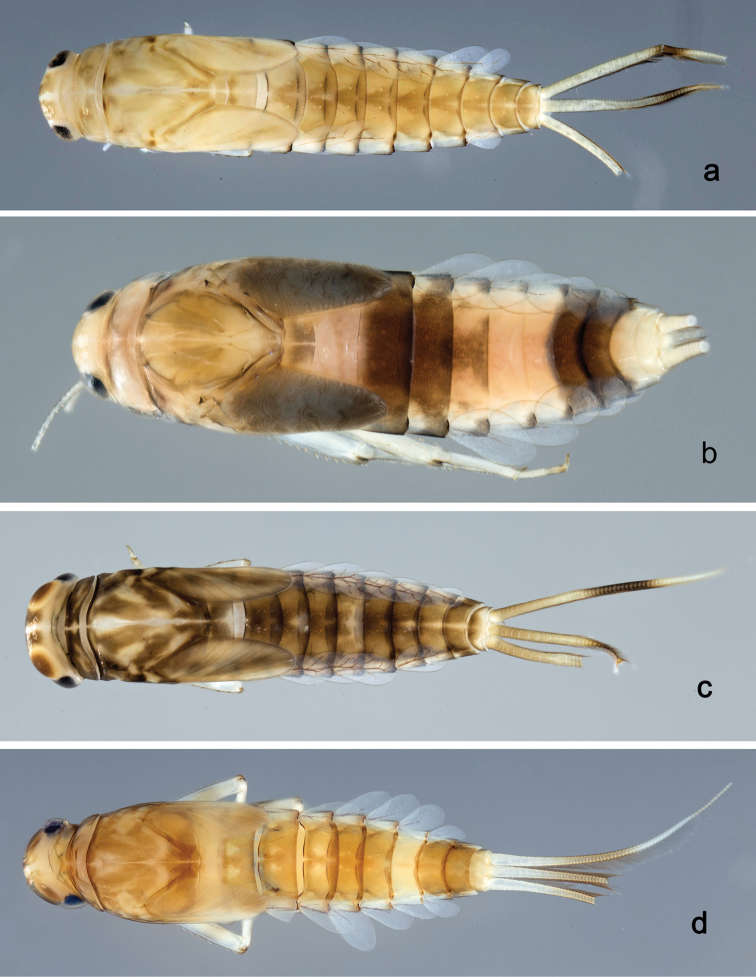
Habitus, larvae, dorsal view: **a***Labiobaetis batakorum* sp. nov. **b***Labiobaetis sulawesiensis* sp. nov. **c***Labiobaetis sumbensis* sp. nov. **d***Labiobaetis roulade* sp. nov.

**Figure 48.**
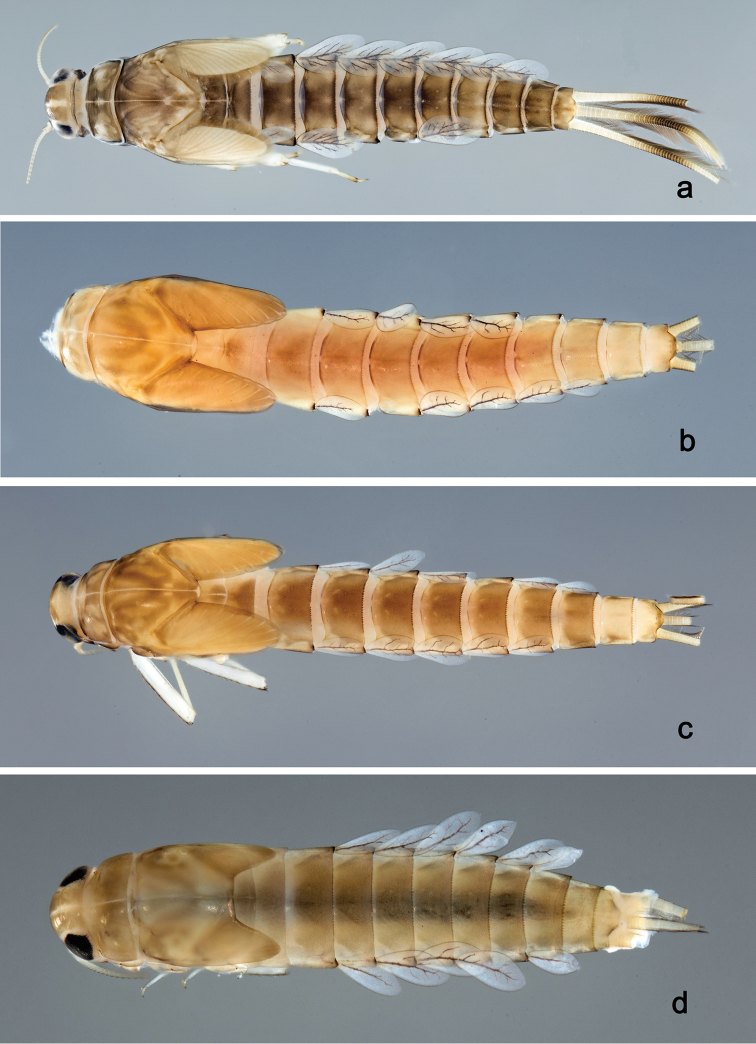
Habitus, larvae, dorsal view: **a***Labiobaetis weifangae* sp. nov. **b***Labiobaetis itineris* sp. nov., without head **c***Labiobaetis lubu* sp. nov. **d***Labiobaetis pakpak* sp. nov.

### Key to the *Labiobaetis* species of Indonesia and adjacent countries (larvae)

**Table:** 

1	Dorsal surface of labrum with submarginal arc of simple setae (Fig. 1a)	**2**
–	Dorsal surface of labrum with submarginal arc of clavate setae (Fig. 1c)	**11**
–	Dorsal surface of labrum with submarginal arc of feathered setae (Fig. 1b)	**17**
2	Spines at posterior margin of abdominal terga mostly rounded; maxillary palp with two segments	**3**
–	Spines at posterior margin of abdominal terga mostly triangular, pointed; maxillary palp with two segments	**5**
–	Spines at posterior margin of abdominal terga mostly triangular, pointed, with medial enhancement (Fig. 27f); maxillary palp with three segments	**10**
3	Right mandible without a hump between prostheca and mola; antennal scape with well-developed distolateral process; labial palp segment II enlargement large, lobed; maxillary palp longer (1.1–1.4×) than length of galea-lacinia	***L. moriharai***
–	Right mandible with a pronounced hump between prostheca and mola; antennal scape without distolateral process; labial palp segment II enlargement thumb-like; maxillary palp much longer (> 1.4×) than length of galea-lacinia	**4**
4	Hypopharynx with medial tuft of stout setae poorly developed; maxillary palp segment II without distolateral excavation; paraproct with 16–19 marginal spines and some submarginal spines; dorsal margin of femur with pectinate setae (difficult to see)	***L. paranumeratus* sp. nov.**
–	Hypopharynx with medial tuft of stout setae well developed; maxillary palp segment II with poorly developed distolateral excavation; paraproct with 12–14 marginal spines; dorsal margin of femur without pectinate setae	***L. numeratus***
5	Hindwing pads absent (Fig. 1v)	**6**
–	Hindwing pads present, minute (Fig. 1w); left mandible with pronounced hump between prostheca and mola (Fig. 33b)	***L. pilosus* sp. nov.**
–	Hindwing pads present, well developed (Fig. 1y); left mandible without hump between prostheca and mola	***L. multus***
6	Seven pairs of gills; left mandible without tuft of setae at apex of mola; antennal scape with well-developed distolateral process; maxillary palp longer (1.1–1.4×) than length of galea-lacinia	**7**
–	Six pairs of gills; left mandible with tuft of setae at apex of mola; antennal scape without distolateral process; maxillary palp approx. as long as length of galea-lacinia	**9**
7	Labial palp segment II enlargement thumb-like; labial palp segment III conical	**8**
–	Labial palp segment II enlargement large, lobed; labial palp segment III oblong	***L. sumbensis* sp. nov.**
8	Labial palp segment III distal margin slightly pointed; maxillary palp segment II without distolateral excavation; paraproct with ca. 21 marginal spines; spines at posterior margin of abdominal terga longer than wide	***L. sulawesiensis* sp. nov.**
–	Labial palp segment III distal margin rounded; maxillary palp last segment with poorly developed distolateral excavation; paraproct with 11–18 marginal spines; spines at posterior margin of abdominal terga wider than long	***L. batakorum* sp. nov.**
9	Paraproct distally not expanded; hypopharynx with medial tuft of stout setae well developed, long (Fig. 1r); labial palp segment II enlargement slender, thumb-like (Fig. 1i); maxillary palp segment II with poorly developed distolateral excavation	***L. seramensis* sp. nov.**
–	Paraproct distally expanded; hypopharynx with medial tuft of stout setae well developed, short (Fig. 1t); labial palp segment II enlargement thumb-like; maxillary palp segment II without distolateral excavation	***L. wahai* sp. nov.**
10	Setae at dorsal margin of femur apically pectinate; labial palp segment III conical; maxillary palp segment II approx. as long as segment I; fore femur ca. 2–3× as long as wide	***L. gueuningi* sp. nov.**
–	Setae at dorsal margin of femur not pectinate; labial palp segment III oblong; maxillary palp segment II longer than segment I; fore femur ca. 4–5× as long as wide	***L. minang* sp. nov.**
11	Paraproct distally not expanded	**12**
–	Paraproct distally expanded	**14**
12	Antennal scape with poorly developed distolateral process; labial palp segment II enlargement large, lobed; maxillary palp longer (1.1–1.4×) than length of galea-lacinia	**13**
–	Antennal scape without distolateral process; labial palp segment II enlargement thumb-like; maxillary palp much longer (> 1.4×) than length of galea-lacinia	***L. rimba* sp. nov.**
13	Labial palp segment III distal margin slightly pointed; maxillary palp segment II with well-developed distolateral excavation; paraproct with ca. 35 marginal spines; tarsus at ventral margin with pectinate setae	***L. diffundus***
–	Labial palp segment III distal margin slightly truncate; maxillary palp segment II with poorly developed distolateral excavation; paraproct with 39-45 marginal spines; tarsus at ventral margin without pectinate setae	***L. molawinensis***
14	Labial palp segment II enlargement thumb-like (Fig. 1h)	**15**
–	Labial palp segment II enlargement large, lobed (Fig. 1g)	**16**
–	Labial palp segment II enlargement hook-like (Fig. 1j)	***L. sumigarensis***
15	Labial palp segment III slightly pentagonal; number of femur dorsal setae on margin ca. 16; labial palp segment III distal margin slightly pointed	***L. paradiffundus* sp. nov.**
–	Labial palp segment III oblong; number of femur dorsal setae on margin 9–12; labial palp segment III distal margin rounded	***L. itineris* sp. nov.**
16	Antennal scape without distolateral process; maxillary palp segment II longer than segment I; maxillary palp segment II with poorly developed distolateral excavation	***L. pakpak* sp. nov.**
–	Antennal scape with poorly developed distolateral process; maxillary palp segment II approx. as long as segment I; maxillary palp segment II with well-developed distolateral excavation	***L. lubu* sp. nov.**
17	Hindwing pads absent (Fig. 1v)	**18**
–	Hindwing pads present, small (Fig. 1×)	***L. borneoensis***
–	Hindwing pads present, well developed (Fig. 1y)	**21**
18	Hypopharynx with medial tuft of stout setae poorly developed (Fig. 1u); labial palp segment III conical	**19**
–	Hypopharynx with medial tuft of stout setae well developed, short (Fig. 1t); labial palp segment III oblong	**20**
19	Antennal scape with poorly developed distolateral process; maxillary palp longer (1.1–1.4×) than length of galea-lacinia; tarsal claw with 11-13 denticles; fore femur ca. 2–3× as long as wide	***L. weifangae* sp. nov.**
–	Antennal scape with well-developed distolateral process; maxillary palp almost as long as length of galea-lacinia; tarsal claw with 15 denticles; fore femur ca. 4–5× as long as wide	***L. roulade* sp. nov.**
20	Maxillary palp approx. as long as length of galea-lacinia; number of setae on dorsal margin of femur ca. 10; labial palp segment III distal margin slightly truncate; fore femur ca. 2–3× as long as wide	***L. difficilis***
–	Maxillary palp longer (1.1–1.4×) than length of galea-lacinia; number of setae on dorsal margin of femur ca. 13; labial palp segment III distal margin slightly pointed; fore femur ca. 4–5× as long as wide	***L. jonasi* sp. nov.**
21	Hypopharynx with medial tuft of stout setae poorly developed; labial palp segment III oblong; number of setae on dorsal margin of femur 11–18; labial palp segment III distal margin rounded	***L. paraoperosus* sp. nov.**
–	Hypopharynx with medial tuft of stout setae well developed; labial palp segment III slightly pentagonal; number of setae on dorsal margin of femur ca. 10; labial palp segment III distal margin slightly truncate	***L. operosus***

### Distribution

The distribution of all species known from Southeast Asia (except southern China, see Shi and Tong 2014) is shown in Figures 53b, 54, and 55; species known only at imaginal stage are not included. The data for the nine species already described are taken from Müller-Liebenau (1982, 1984a, b) and Soldan (1991). *Labiobaetis diffundus* and *L. numeratus* were also reported from China (Shi and Tong 2014) and *L. molawinensis* from Taiwan (Kang et al. 1994). However, based on the illustrations of Kang et al. (1994: fig. 15), this is, in our opinion, neither *L. molawinensis*, as already stated by Shi and Tong (2014), nor *L. diffundus*, as there seems to be no antennal scape process at all. The recent material treated in this study was collected in 50 locations across Indonesia (Fig. 53a). There are still many regions in Indonesia and Southeast Asia in general where no sampling of mayflies has yet been done and many species are known from a single population only. This implies that the current diversity and distribution must be considered as very preliminary. In terms of altitude, the *Labiobaetis* species of Indonesia were found from sea level to mountain areas up to 2,000 m. The GPS coordinates of the locations in Indonesia and adjacent countries are given in Table 2.

**Figure 49.**
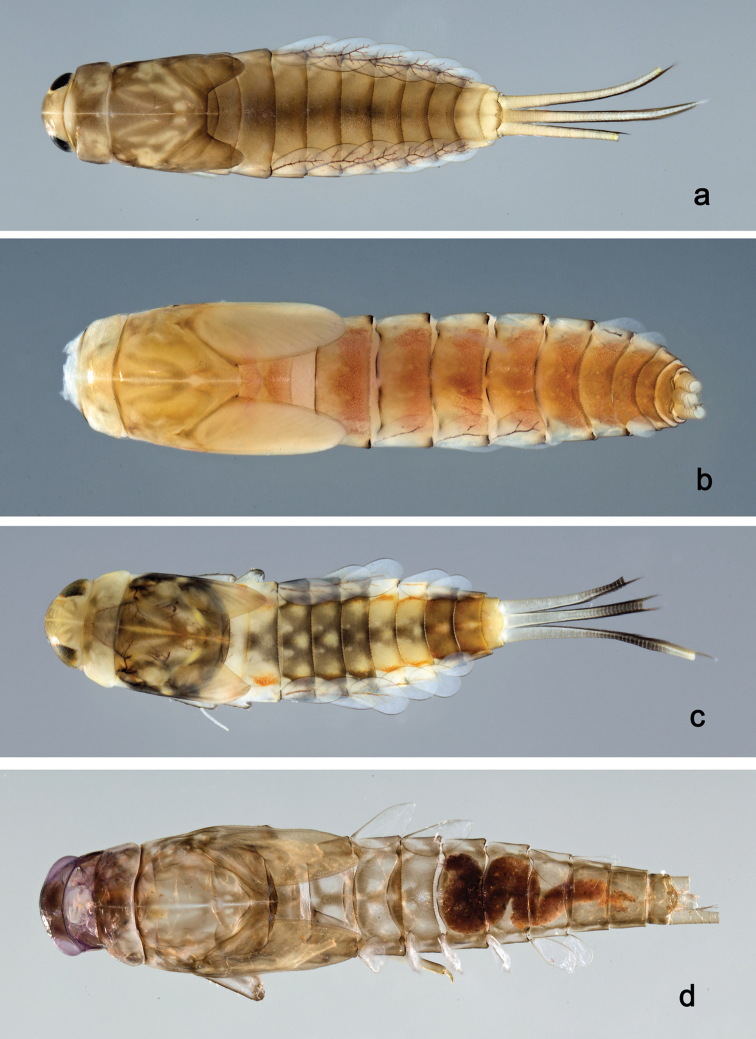
Habitus, larvae, dorsal view: **a***Labiobaetis paradiffundus* sp. nov. **b***Labiobaetis rimba* sp. nov., without head **c***Labiobaetis gueuningi* sp. nov. **d***Labiobaetis minang* sp. nov., after DNA extraction.

**Figure 50.**
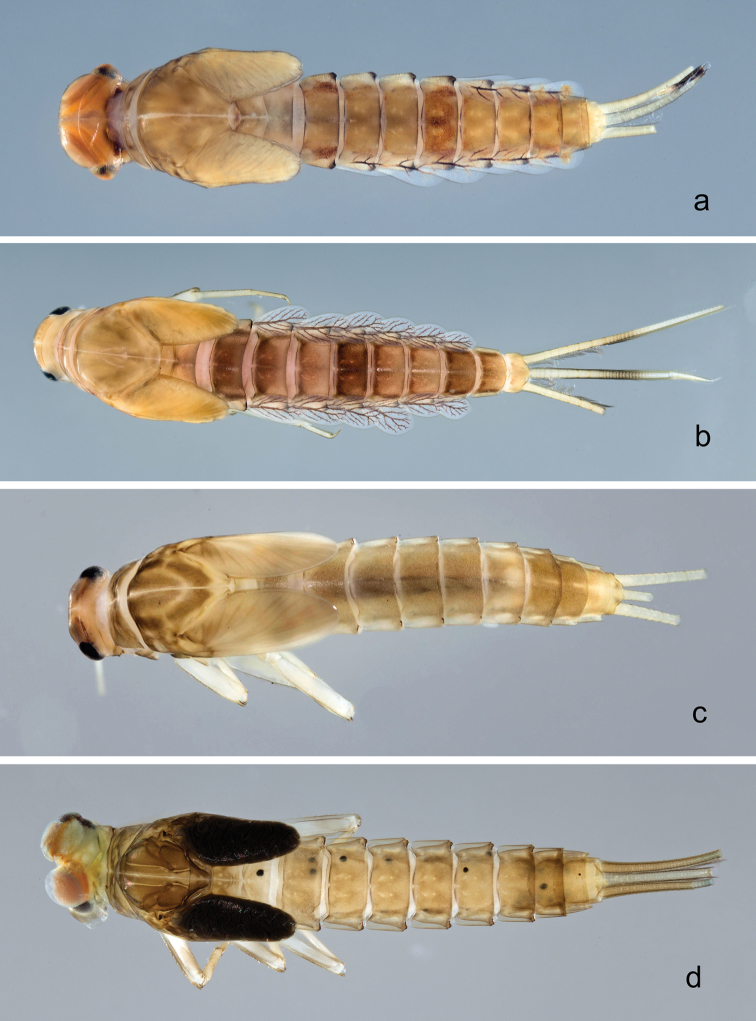
Habitus, larvae, dorsal view: **a***Labiobaetis paranumeratus* sp. nov. **b***Labiobaetis pilosus* sp. nov. **c***Labiobaetis seramensis* sp. nov. **d***Labiobaetis wahai* sp. nov.

**Figure 51.**
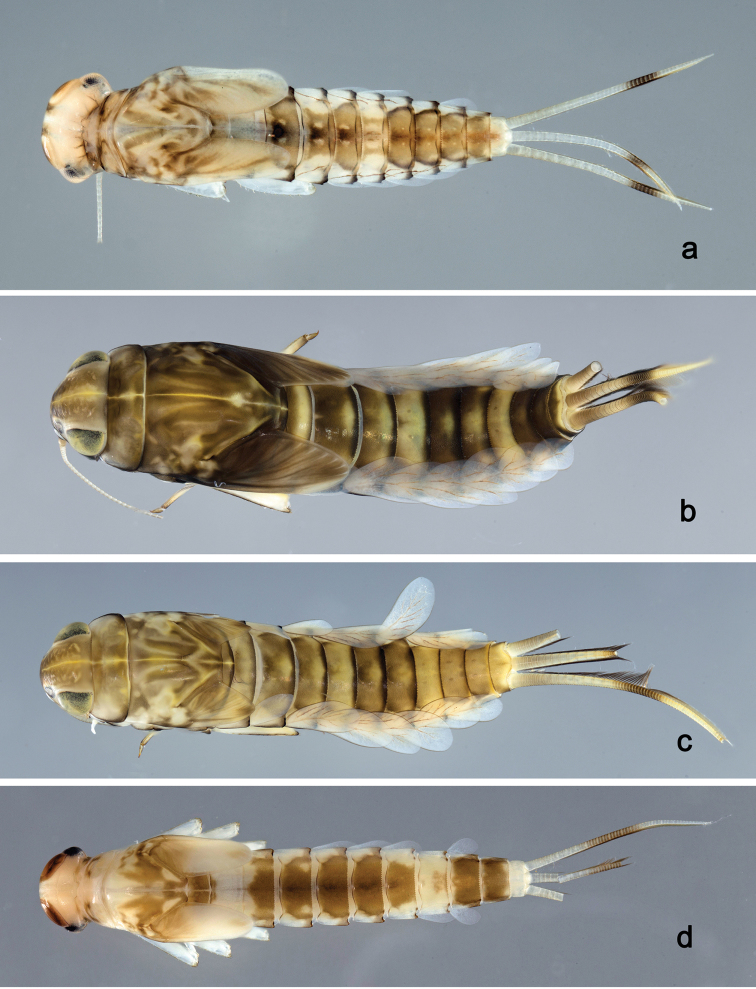
Habitus, larvae, dorsal view: **a***Labiobaetis multus***b***Labiobaetis jonasi* sp. nov. **c***Labiobaetis jonasi* sp. nov. **d***Labiobaetis paraoperosus* sp. nov.

**Table 2.** GPS coordinates of locations of examined specimens.

**Species**	**Locality**	**GPS coordinates**
*L. batakorum* sp. nov.	Sumatra	00°41.33'S, 100°46.72'E
		01°06.03'S, 100°29.12'E
		01°13.62'S, 100°29.83'E
*L. sulawesiensis* sp. nov.	Sulawesi	01°11.75'S, 120°10.20'E
*L. sumbensis* sp. nov.	Sumba	09°38.62'S, 119°40.93'E
		09°26.06'S, 119°18.53'E
		09°35.75'S, 119°20.42'E
*L. difficilis* (Müller-Liebenau)	Malaysia	03°13.07'N, 101°42.75'E
*L. roulade* sp. nov.	Sumatra	00°16.55'S, 100°36.28'E
*L. weifangae* sp. nov.	Sumbawa	08°37.72'S, 118°29.62'E
	Sumba	09°26.06'S, 119°18.53'E
*L. diffundus* (Müller-Liebenau)	Malaysia	03°13.07'N, 101°42.75'E
*L. molawinensis* (Müller-Liebenau)	Philippines	14°08.43'N, 121°28.15'E
*L. sumigarensis* (Müller-Liebenau)	Philippines	17°03.97'N, 121°02.00'E
*L. itineris* sp. nov.	Sumbawa	08°35.87'S, 117°16.68'E
	Bali	08°29.98'S, 115°14.59'E
*L. lubu* sp. nov.	Sumatra	00°56.74'S, 100°32.73'E
*L. pakpak* sp. nov.	Sumatra	00°40.10'S, 101°07.26'E
*L. paradiffundus* sp. nov.	Sumatra	00°23.65'S, 100°25.48'E
		00°18.93'S, 100°40.73'E
		00°18.02'S, 100°40.13'E
		00°24.20'S, 100°22.72'E
		00°23.53'S, 100°21.45'E
		00°22.93'S, 100°22.70'E
		00°23.05'S, 100°21.40'E
		00°19.95'S, 100°19.32'E
		00°05.35'N, 99°58.10'E
*L. rimba* sp. nov.	Sumatra	00°41.33'S, 100°46.72'E
		01°06.03S, 100°29.12'E
*L. gueuningi* sp. nov.	Sumatra	00°18.02'S, 100°40.13'E
		00°22.93'S, 100°22.70'E
		00°19.95'S, 100°19.32'E
		00°24.12'S, 100°16.73'E
		00°23.55'S, 100°16.57'E
		00°22.85'S, 100°17.65'E
*L. minang* sp. nov.	Sumatra	00°23.53'S, 100°21.45'E
		00°23.05'S, 100°21.40'E
*L. numeratus* (Müller-Liebenau)	Malaysia	03°13.07'N, 101°42.75'E
*L. paranumeratus* sp. nov.	Sumatra	00°06.44'S, 100°40.37'E
*L. pilosus* sp. nov.	Sulawesi	01°11.74'S, 120°10.20'E
		01°19.58'S, 120°18.67'E
*L. operosus* (Müller-Liebenau)	Malaysia	03°13.07'N, 101°42.75'E
*L. paraoperosus* sp. nov.	Sumatra	00°41.33'S, 100°46.72'E
		00°21.97'S, 100°33.30'E
		01°13.62'S, 100°29.83'E
		03°40.73'N, 97°39.37'E
*L. seramensis* sp. nov.	Seram	03°10.01'S, 129°28.93'E
*L. wahai* sp. nov.	Seram	03°06.52'S, 129°28.80'E
*L. borneoensis* (Müller-Liebenau)	Borneo	05°54.85'N, 116°06.47'E
*L. moriharai* (Müller-Liebenau)	Malaysia	03°13.07'N, 101°42.75'E
	Vietnam	11°25.40'N, 107°25.72'E
*L. multus* (Müller-Liebenau)	Malaysia	03°13.07'N, 101°42.75'E
	Sumatra	00°06.44'S, 100°40.37'E
		00°34.15'S, 100°43.54'E
*L. jonasi* sp. nov.	Sumba	09°35.74'S, 119°20.41'E

### Genetics

COI sequences were obtained from 13 of the new species (Table 1). The genetic distances (K2P) between these species lie between 11% and 24%, all much higher than 3.5%, which is generally considered as a likely maximal value for intraspecific divergence (Hebert et al. 2003, Ball et al. 2005, Zhou et al. 2010) (Table 3). The exception is *L. gueuningi* sp. nov. with a genetic distance between 1% and 4% only (avg. 2.4%) to *L. minang* sp. nov., despite clear morphological differences. Very limited genetic distances (between 0% and 3%) were found between specimens of the same species, as in *L. sumbensis* sp. nov., *L. weifangae* sp. nov., *L. itineris* sp. nov., *L. pakpak* sp. nov., *L. paradiffundus* sp. nov., *L. gueuningi* sp. nov., *L. minang* sp. nov., *L. paranumeratus* sp. nov. and *L. jonasi* sp. nov. The exceptions are *L. pilosus* sp. nov., and *L. batakorum* sp. nov. Three of the four sequenced specimens of *L. pilosus* sp. nov. have distances from 0% to 1%, but the fourth specimen collected in another location has a distance of 3% to 4% compared to the others. *Labiobaetis batakorum* sp. nov. presents an intraspecific distance of 0% to 4% (avg. 2.5%) between the four sequenced specimens from three different locations.

**Figure 52.**
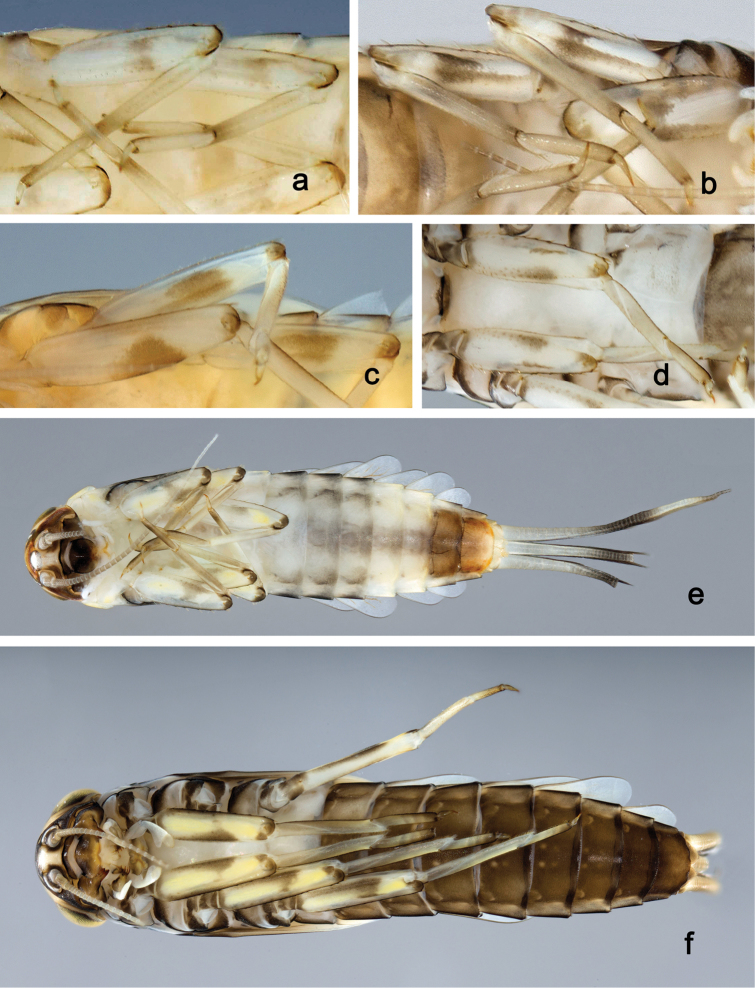
Larvae: **a***Labiobaetis batakorum* sp. nov., ventral view, legs **b***Labiobaetis sumbensis* sp. nov., ventral view, legs **c***Labiobaetis roulade* sp. nov., ventral view, legs **d***Labiobaetis weifangae* sp. nov., ventral view, legs **e***Labiobaetis gueuningi* sp. nov., ventral view **f***Labiobaetis jonasi* sp. nov., ventral view.

**Figure 53.**
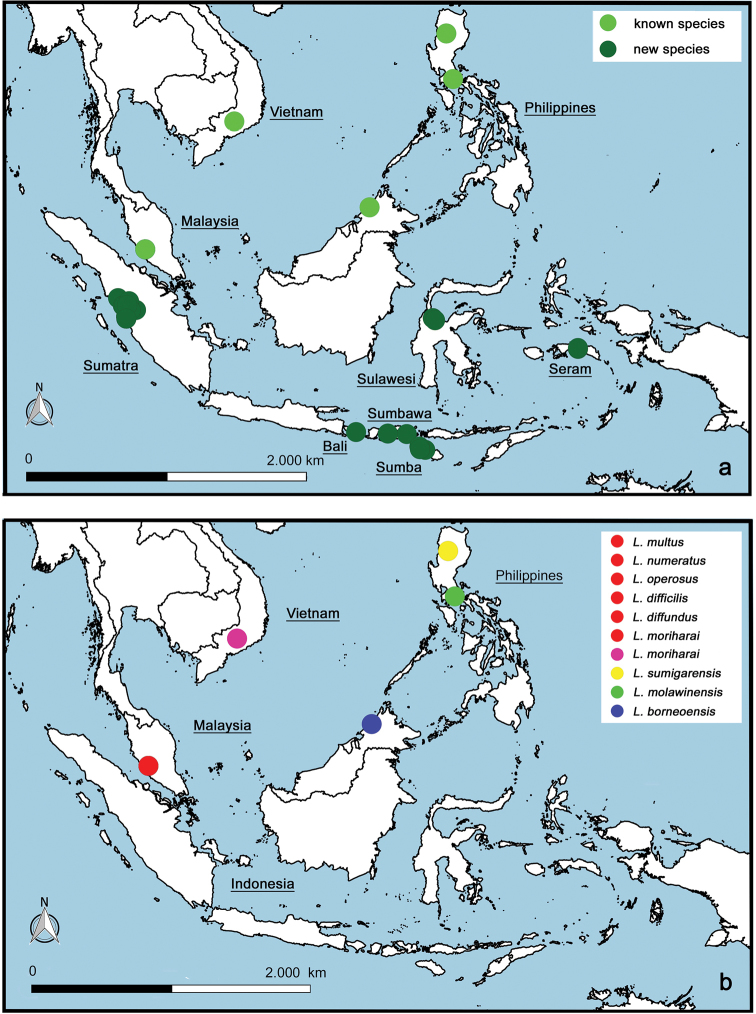
Distribution of *Labiobaetis* in Southeast Asia: **a** localities of all *Labiobaetis* species **b***Labiobaetis* species presently known.

**Table 3.** Genetic distances (COI) between sequenced specimens, using the Kimura 2-parameter.

		**1**	**2**	**3**	**4**	**5**	**6**	**7**	**8**	**9**	**10**	**11**	**12**	**13**	**14**	**15**	**16**	**17**	**18**	**19**	**20**	**21**	**22**	**23**	**24**	**25**	**26**	**27**	**28**	**29**	**30**	**31**	**32**	**33**	**34**	**35**	**36**	**37**	**38**	**39**	**40**
**1**	*L. batakorum* sp. nov.																																								
**2**	*L. batakorum* sp. nov.	0.00																																							
**3**	*L. batakorum* sp. nov.	0.04	0.04																																						
**4**	*L. batakorum* sp. nov.	0.03	0.03	0.01																																					
**5**	*L. sulawesiensis* sp. nov.	0.11	0.11	0.11	0.11																																				
**6**	*L. sumbensis* sp. nov.	0.16	0.16	0.17	0.16	0.16																																			
**7**	*L. sumbensis* sp. nov.	0.16	0.16	0.17	0.16	0.16	0.00																																		
**8**	*L. sumbensis* sp. nov.	0.15	0.16	0.16	0.16	0.16	0.01	0.01																																	
**9**	*L. weifangae* sp. nov.	0.19	0.19	0.19	0.19	0.19	0.23	0.23	0.22																																
**10**	*L. weifangae* sp. nov.	0.19	0.19	0.19	0.19	0.19	0.23	0.23	0.22	0.00																															
**11**	*L. weifangae* sp. nov.	0.19	0.19	0.19	0.19	0.19	0.23	0.23	0.22	0.00	0.00																														
**12**	*L. weifangae* sp. nov.	0.20	0.19	0.20	0.20	0.20	0.23	0.23	0.22	0.00	0.00	0.00																													
**13**	*L. itineris* sp. nov.	0.14	0.14	0.15	0.14	0.13	0.17	0.17	0.17	0.19	0.19	0.19	0.20																												
**14**	*L. itineris* sp. nov.	0.14	0.14	0.15	0.14	0.13	0.17	0.17	0.16	0.19	0.19	0.19	0.20	0.01																											
**15**	*L. lubu* sp. nov.	0.14	0.14	0.14	0.14	0.13	0.15	0.15	0.15	0.20	0.20	0.20	0.21	0.13	0.13																										
**16**	*L. pakpak* sp. nov.	0.19	0.20	0.22	0.21	0.18	0.19	0.19	0.18	0.23	0.23	0.23	0.23	0.19	0.18	0.19																									
**17**	*L. pakpak* sp. nov.	0.19	0.20	0.22	0.22	0.18	0.18	0.18	0.17	0.24	0.24	0.24	0.23	0.19	0.18	0.19	0.01																								
**18**	*L. paradiffundus* sp. nov.	0.19	0.20	0.20	0.20	0.20	0.21	0.21	0.21	0.22	0.22	0.22	0.23	0.21	0.21	0.21	0.20	0.21																							
**19**	*L. paradiffundus* sp. nov.	0.19	0.20	0.20	0.19	0.20	0.21	0.21	0.21	0.22	0.22	0.22	0.23	0.21	0.21	0.21	0.21	0.21	0.00																						
**20**	*L. gueuningi* sp. nov.	0.19	0.19	0.19	0.19	0.18	0.21	0.21	0.20	0.23	0.23	0.23	0.22	0.19	0.19	0.21	0.22	0.22	0.17	0.17																					
**21**	*L. gueuningi* sp. nov.	0.19	0.19	0.19	0.19	0.17	0.22	0.22	0.20	0.23	0.23	0.23	0.22	0.18	0.18	0.20	0.21	0.21	0.20	0.20	0.02																				
**22**	*L. gueuningi* sp. nov.	0.19	0.19	0.19	0.19	0.17	0.22	0.22	0.20	0.23	0.23	0.23	0.23	0.19	0.19	0.20	0.22	0.22	0.21	0.21	0.03	0.01																			
**23**	*L. gueuningi* sp. nov.	0.19	0.19	0.19	0.19	0.17	0.22	0.22	0.20	0.23	0.23	0.23	0.23	0.19	0.19	0.20	0.22	0.22	0.21	0.21	0.03	0.01	0.00																		
**24**	*L. gueuningi* sp. nov.	0.19	0.19	0.19	0.19	0.17	0.22	0.22	0.20	0.23	0.23	0.23	0.23	0.19	0.19	0.20	0.22	0.22	0.21	0.21	0.03	0.01	0.00	0.00																	
**25**	*L. gueuningi* sp. nov.	0.19	0.19	0.19	0.19	0.17	0.22	0.22	0.20	0.23	0.23	0.23	0.22	0.18	0.18	0.20	0.21	0.21	0.20	0.20	0.02	0.00	0.01	0.01	0.01																
**26**	*L. gueuningi* sp. nov.	0.19	0.19	0.19	0.19	0.17	0.22	0.22	0.20	0.23	0.23	0.23	0.23	0.19	0.19	0.20	0.22	0.22	0.21	0.21	0.03	0.01	0.00	0.00	0.00	0.01															
**27**	*L. gueuningi* sp. nov.	0.19	0.19	0.19	0.19	0.17	0.22	0.22	0.20	0.23	0.23	0.23	0.23	0.19	0.19	0.20	0.22	0.22	0.21	0.21	0.03	0.01	0.00	0.00	0.00	0.01	0.00														
**28**	*L. gueuningi* sp. nov.	0.19	0.19	0.19	0.19	0.17	0.22	0.22	0.20	0.23	0.23	0.23	0.23	0.19	0.19	0.20	0.22	0.22	0.21	0.21	0.03	0.01	0.00	0.00	0.00	0.01	0.00	0.00													
**29**	*L. minang* sp. nov.	0.18	0.19	0.19	0.19	0.17	0.22	0.22	0.22	0.24	0.24	0.24	0.24	0.18	0.18	0.20	0.22	0.22	0.20	0.20	0.04	0.02	0.01	0.01	0.01	0.02	0.01	0.01	0.01												
**30**	*L. minang* sp. nov.	0.18	0.19	0.19	0.19	0.17	0.22	0.22	0.22	0.24	0.24	0.24	0.24	0.18	0.18	0.20	0.22	0.22	0.20	0.20	0.04	0.02	0.01	0.01	0.01	0.02	0.01	0.01	0.01	0.00											
**31**	*L. minang* sp. nov.	0.18	0.19	0.19	0.19	0.17	0.22	0.22	0.22	0.24	0.24	0.24	0.24	0.18	0.18	0.20	0.22	0.22	0.20	0.20	0.04	0.02	0.01	0.01	0.01	0.02	0.01	0.01	0.01	0.00	0.00										
**32**	*L. minang* sp. nov.	0.18	0.19	0.19	0.19	0.17	0.22	0.22	0.22	0.24	0.24	0.24	0.24	0.18	0.18	0.20	0.22	0.22	0.20	0.20	0.04	0.02	0.01	0.01	0.01	0.02	0.01	0.01	0.01	0.00	0.00	0.00									
**33**	*L. paranumeratus* sp. nov.	0.15	0.16	0.17	0.17	0.17	0.20	0.20	0.19	0.21	0.21	0.21	0.21	0.17	0.18	0.17	0.20	0.20	0.20	0.20	0.22	0.22	0.22	0.22	0.22	0.22	0.22	0.22	0.22	0.22	0.22	0.22	0.22								
**34**	*L. paranumeratus* sp. nov.	0.15	0.15	0.17	0.17	0.17	0.19	0.19	0.19	0.21	0.21	0.21	0.21	0.18	0.18	0.17	0.19	0.19	0.21	0.21	0.23	0.22	0.22	0.22	0.22	0.22	0.22	0.22	0.22	0.23	0.23	0.23	0.23	0.00							
**35**	*L. pilosus* sp. nov.	0.17	0.17	0.18	0.17	0.17	0.19	0.19	0.19	0.17	0.17	0.17	0.18	0.18	0.18	0.16	0.23	0.22	0.21	0.21	0.20	0.21	0.21	0.21	0.21	0.21	0.21	0.21	0.21	0.21	0.21	0.21	0.21	0.16	0.16						
**36**	*L. pilosus* sp. nov.	0.16	0.16	0.17	0.16	0.17	0.17	0.17	0.17	0.18	0.18	0.18	0.19	0.17	0.18	0.16	0.22	0.21	0.20	0.19	0.18	0.19	0.19	0.19	0.19	0.19	0.19	0.19	0.19	0.19	0.19	0.19	0.19	0.18	0.17	0.03					
**37**	*L. pilosus* sp. nov.	0.17	0.17	0.18	0.17	0.17	0.19	0.19	0.19	0.18	0.18	0.18	0.18	0.18	0.18	0.16	0.23	0.22	0.22	0.21	0.20	0.21	0.21	0.21	0.21	0.21	0.21	0.21	0.21	0.21	0.21	0.21	0.21	0.16	0.16	0.01	0.04				
**38**	*L. pilosus* sp. nov.	0.17	0.17	0.18	0.17	0.17	0.19	0.19	0.19	0.17	0.17	0.17	0.18	0.18	0.18	0.16	0.23	0.22	0.21	0.21	0.20	0.21	0.21	0.21	0.21	0.21	0.21	0.21	0.21	0.21	0.21	0.21	0.21	0.16	0.16	0.00	0.03	0.01			
**39**	*L. multus* (Müller-Liebenau)	0.13	0.13	0.13	0.13	0.13	0.15	0.15	0.15	0.22	0.22	0.22	0.22	0.15	0.14	0.16	0.19	0.19	0.19	0.19	0.17	0.16	0.16	0.16	0.16	0.16	0.16	0.16	0.16	0.16	0.16	0.16	0.16	0.16	0.16	0.17	0.16	0.17	0.17		
**40**	*L. jonasi* sp. nov.	0.19	0.19	0.19	0.19	0.21	0.22	0.22	0.22	0.17	0.17	0.17	0.17	0.19	0.20	0.20	0.23	0.23	0.21	0.21	0.20	0.21	0.22	0.22	0.22	0.21	0.22	0.22	0.22	0.22	0.22	0.22	0.22	0.20	0.20	0.18	0.18	0.19	0.18	0.21	
**41**	*L. jonasi* sp. nov.	0.19	0.19	0.19	0.19	0.21	0.22	0.22	0.22	0.17	0.17	0.17	0.17	0.19	0.20	0.20	0.23	0.23	0.21	0.21	0.20	0.21	0.22	0.22	0.22	0.21	0.22	0.22	0.22	0.22	0.22	0.22	0.22	0.20	0.20	0.18	0.18	0.19	0.18	0.21	0.00

## Discussion

For the assignment of the new species to *Labiobaetis* we refer to Kluge and Novikova (2014), Müller-Liebenau (1984a), and McCafferty and Waltz (1995). *Labiobaetis* is characterized by a number of derived characters, some of which are not found in other taxa (Kluge and Novikova 2014): antennal scape sometimes with a distolateral process (Fig. 1d–f); maxillary palp two-segmented with an excavation at inner distolateral margin of segment II, excavation may be poorly developed or absent (Figs 1 o–q); labium with paraglossae widened and glossae diminished; and labial palp segment II with distomedial protuberance (Figs 1 g–j). All these characters vary and may be secondarily lost (Kluge and Novikova 2014). Two of the species described in this study (*L. gueuningi* sp. nov. and *L. minang* sp. nov.) have a maxillary palp with three segments, which was also described from *Labiobaetis boussoulius* (Gillies) from Guinea (Gillies 1993). The concept of *Labiobaetis* is also based on additional characters (Müller-Liebenau 1984a, McCafferty and Waltz 1995, Lugo-Ortiz and McCafferty 1997, Lugo-Ortiz et al. 1999, Kaltenbach and Gattolliat 2018), slightly amended based on the discovery of the 18 new species: dorsal surface of labrum with submarginal setae arranged in one arc, the setae may belong to a simple, pointed type, a feathered type, a dendritic type, a spatulate/clavate type (apically pectinate or smooth) or a lanceolate type (apically pectinate or not pectinate) (Kaltenbach and Gattolliat 2018: fig. 1a–f; mandibles with fused incisors, right prostheca apically denticulate, left prostheca apically denticulate and with a comb-shaped structure; hypopharynx with medial tuft of stout setae at apex of median lobe, the tuft may be well developed and of different lengths or poorly developed (Fig. 1r–u); paraglossae sub-rectangular, slightly curved inward; hindwing pads well developed, small, minute, vestigial or absent (Fig. 1v–y); femoral patch well developed, rudimentary or absent; tibia at apical margin with a tuft of fine, simple setae; tarsal claw distally pointed with one row of denticles, striation present, subapical setae absent; abdominal terga with irregular rows of numerous U-shaped or rarely W-shaped scale bases, posterior margin with regular, triangular, pentagonal or rounded spines; gills on abdominal segment I present or absent; paraproct with ten to more than 40 marginal spines, lateral ones always smaller and distally expanded, slightly expanded or not expanded at all (Fig. 1z1, z2; Kaltenbach and Gattolliat 2018: fig. 1q–s). Three species from Indonesia (*L. gueuningi* sp. nov., *L. paranumeratus* sp. nov., and *L. roulade* sp. nov.) have apically pectinate setae dorsally at the femur margin and *L. paranumeratus* sp. nov. as well as dorsally on the tibia, but not bipectinate setae like species of *Indocloeon* Müller-Liebenau, 1982 (Kluge 2012, Kaltenbach and Gattolliat 2017). Three other species from Indonesia (*L. itineris* sp. nov., *L. paradiffundus* sp. nov., and *L. rimba* sp. nov.) have bipectinate setae ventrally on the tarsus margin like species of *Indocloeon* (Kluge 2012, Kaltenbach and Gattolliat 2017), but no pectinate setae elsewhere on the legs.

The seven species groups proposed in this paper are based on the combination of the types of setae composing the submarginal arc of setae on the dorsal surface of the labrum and the shapes of the distomedial protuberance of labial palp segment II, together with other characters. These morphological groups within *Labiobaetis* are primarily a working tool but could also serve as a basis for future studies on the generic delimitation and phylogeny of this probably polyphyletic genus. The inclusion of nuclear gene sequences may prove that some may be natural groups. So far there was no overlap of any species distribution found between New Guinea and Southeast Asia of *Labiobaetis*, nor any shared species groups. The *sumigarensis* group was proposed by Müller-Liebenau and Hubbard (1985) as a subgroup of the Oriental *Baetis molawinensis* group, based on the concave outer margin of the large, thumb-like or lobed protuberance of labial palp segment II. They included *L. sumigarensis*, *L. diffundus*, *L. geminatus* (Müller-Liebenau and Hubbard), and an undescribed species from Madagascar in that subgroup. Later, the *molawinensis* group formed the basis of *Labiobaetis* together with the European *Baetis atrebatinus* group and the North American *Baetis propinquus* group (Novikova and Kluge 1987, McCafferty and Waltz 1995). In our study on *Labiobaetis* from New Guinea (Kaltenbach and Gattolliat 2018) we placed *L. molawinensis* (Müller-Liebenau) in the *balkei* group of species from New Guinea. However, examination of a paratype of *L. molawinensis* revealed that it should be placed in the *sumigarensis* group. All characters of the *sumigarensis* group as described in this paper are present in *L. molawinensis*; the setae of the submarginal arc on the dorsal surface of the labrum are apically smooth and do not show an apical pectination as in the *balkei* group, which can be assumed from the drawing in Müller-Liebenau (1982: fig. 4A). Furthermore, *L. molawinensis* has no setae at the apex of the mola of the left mandible, in line with the *sumigarensis* group and contrary to the *balkei* group, a character which is not shown in the drawing of Müller-Liebenau (1982: fig. 4G). Apart from these two characters, both groups are in fact rather similar.

The *seraminensis* group is also very similar to the *claudiae* group from New Guinea (shape of the labial palp segment II; the setae of the submarginal arc on the dorsal surface of the labrum are also simple; no hindwing pads, six pairs of gills, no scape process). However, the *seramensis* group has the usual fine setae of uniform length at the gills margin and no femoral patch and the *claudiae* group has both shorter and longer fine setae at the gills margin and a well-developed femoral patch.

*Labiobaetis borneoensis* (Müller-Liebenau) has a lobed distomedial protuberance at labial palp segment II combined with feathered setae of the submarginal arc on the dorsal surface of the labrum as seen in the two species of the *orientis* group from New Guinea and was, therefore, placed in this group (Kaltenbach and Gattolliat 2018). However, the differences between *L. borneoensis* and the *orientis* group are too important to uphold this assignment with the knowledge of the new groups in Indonesia: *L. borneoensis* has seven pairs of gills, an antennal scape process, and hindwing pads, none of which are present in the *orientis* group. We therefore do not propose an assignment for *borneoensis* to any group at the moment.

All other species groups from New Guinea (*petersorum* group, *tuberpalpus* group, *vitilis* group, *vultuosus* group) significantly differ from any other group or species from Indonesia. The *numeratus* group is mainly characterized by a remarkable trait, a pronounced hump between prostheca and mola of the right mandible (Figs 31b, 33b; see also Müller-Liebenau 1984a: fig. 11e; Shi and Tong 2014: fig. 24). A similar hump is present in *Offadens soror* (Ulmer) and *Offadens sobrinus* Lugo-Ortiz and McCafferty, but less pronounced. However, other characters such as the stick-like right prostheca and the missing tuft on the medial lobe of the hypopharynx differentiate them from the *numeratus* group and from *Labiobaetis* (Suter 1986, Fig. 21; Lugo-Ortiz and McCafferty 1998, Webb and Suter 2011, Shi and Tong 2014).

Among the other Oriental species outside Indonesia and adjacent countries, *L. mustus* (Kang and Yang) from Taiwan cannot be assigned to any species group. It is mainly characterized by a notch at segment II of the maxillary palp. The setae of the submarginal arc on the dorsal surface of the labrum are of the same type as in the species of the *balkei* group from New Guinea (spatulate with pronounced apical pectination), but the labial palp segment II is thumb-like and not lobed as in the *balkei* group and it has a well-developed antennal scape process (Kang and Yang 1996, Kaltenbach and Gattolliat 2018). *Labiobaetis ancoralis* Shi and Tong from China is also not similar to any of the species groups or described species. It shares the type of setae of the submarginal arc on the dorsal surface of the labrum with *L. mustus* (spatulate with apical pectination) and the *balkei* group from New Guinea, but has, amongst other differences, well developed hindwing pads (Shi and Tong 2014).

Concerning the species from India and Sri Lanka, *L. ordinatus* (Müller-Liebenau and Hubbard) shares the pronounced hump between prostheca and mola of the right mandible and all other characters of the *numeratus* group (setae of the submarginal arc on the dorsal surface of the labrum simple, thumb-like protuberance of labial palp segment II, absence of scape process, six pairs of gills, minute hindwing pads) and, therefore, probably belongs to this group. The species is easily differentiated from the other species of that group by the distinctive spines (triangular, some very long, sharply pointed) at the posterior margin of the terga and other characters (Figs 31–34; Müller-Liebenau and Hubbard 1985: figs 7, 24, Shi and Tong 2014). *Labiobaetis geminatus* (Müller-Liebenau and Hubbard) is part of the *sumigarensis* group. The species is differentiated from the other species of this group by its combination of characters: the shape of the protuberance at labial palp segment II, the reduced number of setae forming the submarginal arc on the dorsal surface of the labrum (ca. 12), the shape of maxillary palp segment II, tarsus ventrally without pectinate setae (Figs 16–25; Müller-Liebenau and Hubbard 1985: figs 5, 22, Shi and Tong 2014).

*Labiobaetis jacobusi* Kubendran and Balasubramanian has all characters of the *sumigarensis* group and most certainly belongs to it. The differences to the other species of the group are based on several characters, especially the number of setae forming the submarginal arc on the dorsal surface of the labrum (only ca. 12 in *L. jacobusi*), the tuft of stout setae at the medial lobe of the hypopharynx (long in *L. jacobusi*), the number of setae at the dorsal margin of the femur (only ca. seven in *L. jacobusi*), the shape of the paraproct and the shape of the labial palp (Figs 16–25; Kubendran et al. 2015: figs 42–53). *Labiobaetis pulchellus* (Müller-Liebenau and Hubbard) cannot be assigned to any of the groups based on its combination of characters. However, the species presents morphological similarities with *L. multus*. Differences between both are the shape of the labial palps, the dorsal setation of the legs, and the setae at the apex of the mola of the left mandible (present in *L. multus*, absent in *L. pulchellus*) (Müller-Liebenau 1984a: fig. 9; Müller-Liebenau and Hubbard 1985: fig. 6).

*Labiobaetis soldani* Kubendran et al. from India cannot be assigned to a species group because of its combination of characters and is clearly differentiated from all other species (Kubendran et al. 2014).

As a whole, the new species confirm the remarkable morphological differences between the species from Southeast Asia and New Guinea. In New Guinea there are no species with an antennal scape process, all but one species have only six pairs of gills, and there are no species with hindwing pads. In Southeast Asia as well as in other regions, these character states are more evenly distributed and there are at least several species with or without an antennal scape process, with six or seven pairs of gills, and with or without hindwing pads. The main types of setae of the submarginal arc on the dorsal surface of the labrum (simple, feathered, clavate) are almost equally represented in the Oriental realm, whereas the simple type is largely dominant in New Guinea and the feathered type prevailing in the Afrotropical region (Lugo-Ortiz and McCafferty 1997, Gattolliat 2001, Kaltenbach and Gattolliat 2018). Additionally, the species from Southeast Asia have tendentially a limited number of setae at the dorsal margin of the femur (usually fewer than 20 and often fewer than 12 setae), whereas the species from New Guinea have tendentially a high number of these setae (the majority of species have more than 20 setae, sometimes even more than 40, but only in one case fewer than 12). Interestingly, *L. seramensis* sp. nov. and *L. wahai* sp. nov. from Seram (both forming the *seramensis* group) have a closer morphological similarity with the species from New Guinea, especially with the species of the *claudiae* group, than with the other species from Indonesia. They have simple setae forming the submarginal arc on the dorsal surface of the labrum, no antennal scape process, six pairs of gills and no hindwing pads like the vast majority of species from New Guinea. Additionally, they both have more than 20 setae at the dorsal margin of the femur like the majority of species from New Guinea. These characters together with the geographical proximity of Seram to New Guinea points to an eventual colonization of Seram from New Guinea.

From the 16 species of *Labiobaetis* (or previously assigned to *Pseudocloeon*) only known at the imaginal stage, four were described from Indonesia (*P. fulmeki* Ulmer, *P. necopinatum* Müller-Liebenau, *P. ulmeri* Müller-Liebenau from Sumatra, and *P. obscurum* Ulmer from Java and Sumatra) (Ulmer 1913, 1939, Müller-Liebenau 1981). As the identification of the imaginal stage of *Labiobaetis* is generally very difficult, we consider it unrealistic to safely associate the larval stage with old type material at the imaginal stage. In this case, rearing material will provide little help. Furthermore, the generic assignment of these species remains questionable in most of the cases. We, therefore, did not take these species into account in our study and wait for an eventual clarification of their status in the future by using ancient DNA methods.

In general, the genetic distances between the different species of *Labiobaetis* are rather high in Indonesia, between 11% and 24% (K2P, Table 3), which is in line with the genetic distances found in New Guinea (avg. 22%; Kaltenbach and Gattolliat 2018). Ball et al. (2005) reported a mean interspecific, congeneric distance of 18% for mayflies from the United States and Canada. There is an exception, *L. gueuningi* sp. nov. and *L. minang* sp. nov. have a very low interspecific distance between 1% and 4% (avg. 2.4%). Both species were collected in the same area, but in different altitudes, *L. gueuningi* sp. nov. between 840 m and 1,300 m and *L. minang* between 1,640 m and 1,790 m. Despite their clear morphological differences (Figs 26–29), they are morphologically similar to each other and easily distinguishable from other *Labiobaetis* species by the 3-segmented maxillary palp (Figs 26g, 28g). Their small genetic distance and morphological similarities may reflect a recent speciation event (Ball et al. 2005). Very young species pairs might be difficult to identify using COI, especially if the species have ancestrally polymorphic mitochondrial haplotypes that do not sort according to subsequent speciation events (Funk and Omland 2003, Ball et al 2005). The small interspecific genetic distance could be also the consequence of lower substitution rates in these taxa, the involvement of hybrids or mitochondrial introgression (Ball et al 2005, Dussex et al. 2015, Gattolliat et al. 2016). A very small interspecific distance (K2P 0.8%) was also reported in Gattolliat et al. (2016) for Swiss stoneflies and another case (K2P 2.77%) was reported in Zhou et al. (2010) for a Baetidae species (USA). The collection of both species on different elevation levels points to the direction that elevation could be a driver in their speciation. In the same area (volcano Singgalang in Sumatra), elevation was the only factor found to be driving within-species genetic structuring of two Baetidae species and an important factor for two others (Gueuning et al. 2017).

The intraspecific distances are very low as expected, ranging from 0 % to 2% (K2P). This result is certainly biased as it is based on a limited number of sequenced specimens per species, which were mostly from a single population. But there are two exceptions, *L. pilosus* sp. nov., where one specimen from another location has an intraspecific distance of 3% to 4%, and *L. batakorum* sp. nov. with distances of 0% to 4% (avg. 2.5%) between 4 specimens from 3 different locations. Compared to the usual distances between different *Labiobaetis* species in that region this distance can be still considered as intraspecific. Ball et al. (2005) also reported a case with 6% intraspecific distance in a mayfly in North America and intraspecific K2P distances of more than 3.5% are also not uncommon within Plecoptera (Gill et al. 2015, Gattolliat et al. 2016).

In addition to the 18 new species described in this paper, we obtained two additional COI sequences with clearly interspecific genetic distance to other specimens with similar morphology. In one case, one specimen from Sumbawa cannot be morphologically separated from *L. lubu* sp. nov. from Sumatra, but the two haplotypes present a K2P distance of 20%. In the other case, one specimen from Sumatra is morphologically identical to *L. itineris* sp. nov. from Sumbawa and Bali, but with a K2P distance of 12%. Therefore, these have to remain species hypotheses for now without further treatment in this paper. Additional material will be necessary to confirm their status in the future. We also have specimens of five additional undescribed species, which have significant morphological differences to the closest species. Unfortunately, the material is insufficient or partly damaged and we could not extract DNA. We therefore refrain from describing them.

**Figure 54.**
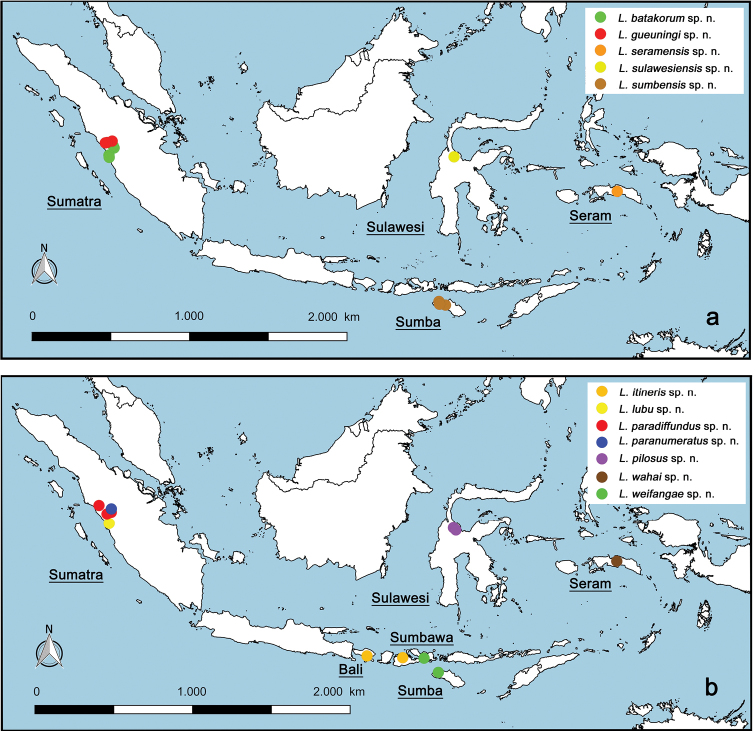
Distribution of *Labiobaetis* in Indonesia.

**Figure 55.**
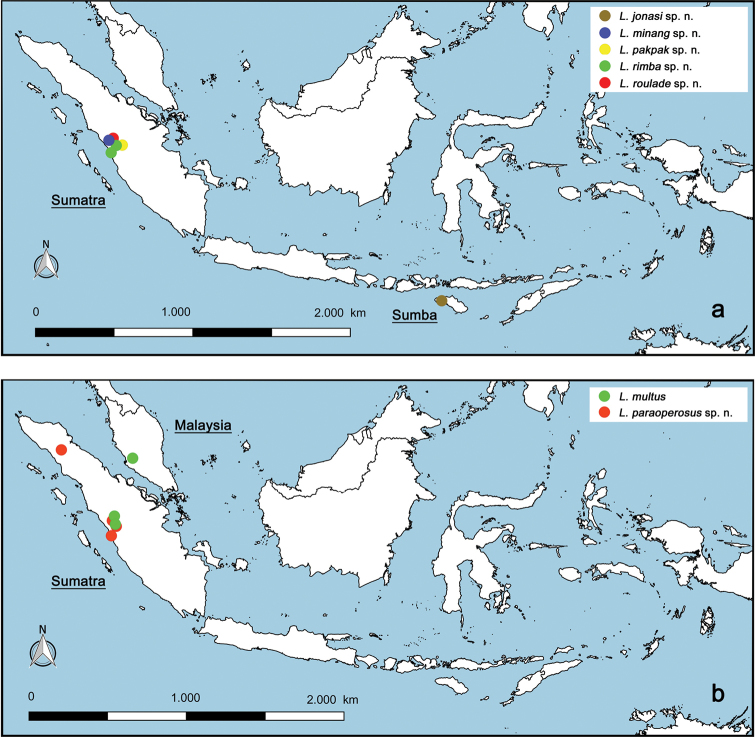
Distribution of *Labiobaetis* in Indonesia.

In the majority of cases, the species distribution of *Labiobaetis* in Indonesia seems to be restricted to a single island (Figs 54, 55), which is fully in line with other Baetidae genera having an insular endemicity close to 100% in the Australasian realm (Gattolliat and Nieto 2009). Exceptions are *L. itineris* sp. nov. from Bali and Sumbawa, *L. weifangae* sp. nov. from Sumbawa and Sumba and *L. multus* from Sumatra and Malaysia, but all these locations are not far away from each other. We consider the distribution of the different species still extremely preliminary, and new sampling may substantially increase the species distribution as it was the case for African and Malagasy representatives of *Labiobaetis* (Lugo-Ortiz and McCafferty 1997, Gattolliat 2001, Kaltenbach and Gattolliat 2018). Interestingly, for four of the six species described from the Gombak River in Malaysia by Müller-Liebenau (1984a), we describe morphologically similar species from Sumatra: *L. roulade* sp. nov. (similar to *L. difficilis*), *L. paradiffundus* sp. nov. (similar to *L. diffundus*), *L. paranumeratus* sp. nov. (similar to *L. numeratus*), and *L. paraoperosus* sp. nov. (similar to *L. operosus*). In the case of *L. multus*, we were unable to find morphological differences to separate populations from Malaysia and Sumatra. At the opposite, the Afrotropical species *L. glaucus* (Agnew) presents a widespread distribution reaching from South Africa to Saudi Arabia, even including the Comoros Islands (Lugo-Ortiz and McCafferty 1997, Gattolliat et al. 2018). The species in China have a more widespread distribution as well, and in the case of *L. mustus* it includes the provinces Hainan and Guangdong, Hong-Kong and even Taiwan (Shi and Tong 2014: fig. 28).

This high level of micro- to meso-endemism restricted to single islands in Indonesia (Figs 54, 55) confirms the situation of the genus in New Guinea, where micro-endemism restricted to smaller areas was found as well (Kaltenbach and Gattolliat 2018), and indicates that allopatry could be a major driver of diversity within this genus. The main difference between Indonesia and New Guinea is that Indonesia consists of numerous small to big islands spread over a large area and New Guinea is one large main island with smaller adjacent islands. For New Guinea, large studies on the highly diversified diving beetle genus *Exocelina* Balke, 1998 (Coleoptera, Dytiscidae) demonstrated allopatry to be the main mechanism of diversification and found strong evidence that recent environmental change in the extremely structured central highlands of New Guinea, with its ongoing formation of rich aquatic resources and remote valleys and mountain blocks, was the primary driver of diversification in that area (Toussaint et al. 2013, 2014). There is also evidence that species in running waters are weaker dispersers than species living in standing water, which has been suggested to promote allopatric speciation and micro-endemism in the first group and dispersal in the second group (Ribera et al. 2001, Monaghan et al. 2005). *Labiobaetis* species mainly live in running waters, but there are a few exceptions. However, their dispersal ability seems to be high enough to have reached remote islands like Vanuatu (Gattolliat and Staniczek 2011) and Fiji (Flowers 1990) in the past and bidirectional transoceanic dispersal between Madagascar and Africa has been shown as well (Monaghan et al. 2005). Additionally, parthenogenesis has been assumed in the genus, which may favour successful dispersal events (Sivaramakrishnan et al. 1991, Gattolliat and Staniczek 2011).

Despite covering an important part of Indonesia, the sampling effort and the number of localities and different habitats is still extremely limited and there are large areas without any collection activities so far (Fig. 53a). In addition, we have seven species hypotheses based on genetics only or based on morphological differences without genetics, which may be confirmed as separate species in the future. Therefore, we may assume that the number of *Labiobaetis* species in Indonesia will continue to increase substantially with further collections in the future.

## Supplementary Material

XML Treatment for Labiobaetis batakorum

XML Treatment for Labiobaetis sulawesiensis

XML Treatment for Labiobaetis sumbensis

XML Treatment for Labiobaetis difficilis

XML Treatment for Labiobaetis roulade

XML Treatment for Labiobaetis weifangae

XML Treatment for Labiobaetis diffundus

XML Treatment for Labiobaetis molawinensis

XML Treatment for Labiobaetis sumigarensis

XML Treatment for Labiobaetis itineris

XML Treatment for Labiobaetis lubu

XML Treatment for Labiobaetis pakpak

XML Treatment for Labiobaetis paradiffundus

XML Treatment for Labiobaetis rimba

XML Treatment for Labiobaetis gueuningi

XML Treatment for Labiobaetis minang

XML Treatment for Labiobaetis numeratus

XML Treatment for Labiobaetis paranumeratus

XML Treatment for Labiobaetis pilosus

XML Treatment for Labiobaetis operosus

XML Treatment for Labiobaetis paraoperosus

XML Treatment for Labiobaetis seramensis

XML Treatment for Labiobaetis wahai

XML Treatment for Labiobaetis borneoensis

XML Treatment for Labiobaetis moriharai

XML Treatment for Labiobaetis multus

XML Treatment for Labiobaetis jonasi
